# Molecular markers in keratins from Mysticeti whales for species identification of baleen in museum and archaeological collections

**DOI:** 10.1371/journal.pone.0183053

**Published:** 2017-08-30

**Authors:** Caroline Solazzo, William Fitzhugh, Susan Kaplan, Charles Potter, Jolon M. Dyer

**Affiliations:** 1 Museum Conservation Institute, Museum Support Center, Smithsonian Institution, Suitland, Maryland, United States of America; 2 Arctic Studies Center, National Museum of Natural History, Department of Anthropology MRC 112, Smithsonian Institution, Washington D.C., United States of America; 3 The Peary-MacMillan Arctic Museum and Arctic Studies Center, Bowdoin College, Brunswick, Maine, United States of America; 4 Department of Vertebrate Zoology, Division of Mammals, National Museum of Natural History, MRC 108, Smithsonian Institution, Washington DC, United States of America; 5 Food & Bio-Based Products, AgResearch, Lincoln Research Centre, Christchurch, New Zealand; Seoul National University College of Medicine, REPUBLIC OF KOREA

## Abstract

Baleen has been harvested by indigenous people for thousands of years, as well as collected by whalers as an additional product of commercial whaling in modern times. Baleen refers to the food-filtering system of Mysticeti whales; a full baleen rack consists of dozens of plates of a tough and flexible keratinous material that terminate in bristles. Due to its properties, baleen was a valuable raw material used in a wide range of artefacts, from implements to clothing. Baleen is not widely used today, however, analyses of this biomolecular tissue have the potential to contribute to conservation efforts, studies of genetic diversity and a better understanding of the exploitation and use of Mysticeti whales in past and recent times. Fortunately, baleen is present in abundance in museum natural history collections. However, it is often difficult or impossible to make a species identification of manufactured or old baleen. Here, we propose a new tool for biomolecular identification of baleen based on its main structural component alpha-keratin (the same protein that makes up hair and fingernails). With the exception of minke whales, alpha-keratin sequences are not yet known for baleen whales. We therefore used peptide mass fingerprinting to determine peptidic profiles in well documented baleen and evaluated the possibility of using this technique to differentiate species in baleen samples that are not adequately identified or are unidentified. We examined baleen from ten different species of whales and determined molecular markers for each species, including species-specific markers. In the case of the Bryde’s whales, differences between specimens suggest distinct species or sub-species, consistent with the complex phylogeny of the species. Finally, the methodology was applied to 29 fragments of baleen excavated from archaeological sites in Labrador, Canada (representing 1500 years of whale use by prehistoric people), demonstrating a dominance of bowhead whale (*Balaena mysticetus*) in the archaeological assemblage and the successful application of the peptide mass fingerprinting technique to identify the species of whale in unidentified and partially degraded samples.

## Introduction

Beginning in the 1990s, museum collections and in particular natural history specimens have become important resources to address questions of evolution, lineages and population genetics, as well as issues linked to ecology, responses to climate change, conservation, loss of genetic diversity and population declines [1–6]. Advances in genetics (e.g., high-throughput sequencing) have allowed for the recovery of maximum amounts of genetic data from minimal sample sizes, thus helping address issues of DNA degradation and contamination in ancient or damaged specimens [1, 3, 4, 6–8]. Genetic material has been retrieved from a variety of tissues from museum specimens: bones and teeth, plant tissues, insects, feathers and skins [1]. DNA has also been successfully recovered from baleen plates, some over a hundred years old [9, 10], and from historical [11] and archaeological [12, 13] artefacts fashioned from baleen. This work has shown that analyses of baleen can contribute to studies of population genetics, conservation and exploitation and use of Mysticeti whales in past and recent times. However, studies have also suggested a significant degradation of DNA (in particular nuclear DNA) in historical baleen compared to fresh specimens [8, 14].

**Fig 1:**
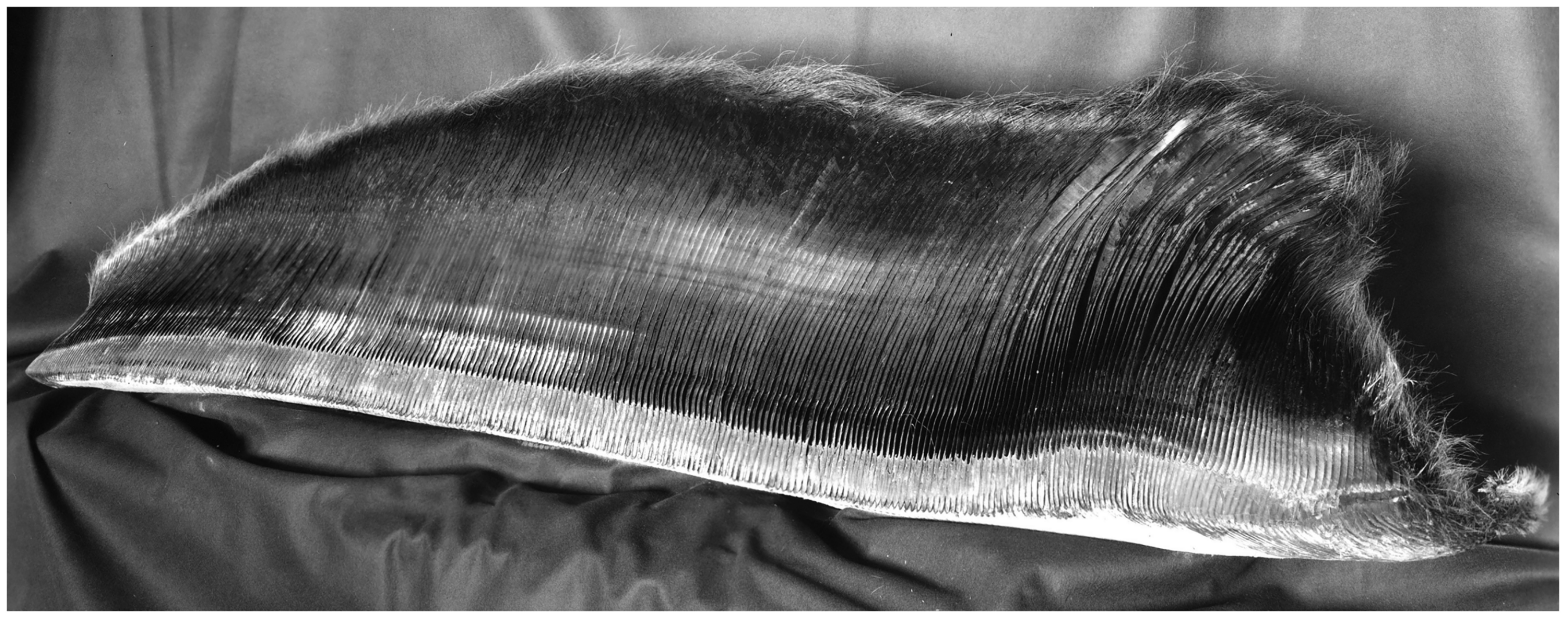
Baleen rack of specimen USNM 267999, an 11 m humpback whale (*Megaptera novaeangliae*) collected in Western Australia, 1938.

Baleen refers to the food-filtering apparatus of Mysticeti whales [15] (taxonomy in S1 File): a full baleen rack consists of dozens of plates (thin sheets packed with longitudinal tubules and covered with a layer of horn-like material) made of a tough and flexible keratinous material [15–17]. The plates are terminated by bristles, resulting when the horn cover is worn out and the tubules exposed (Fig 1). Recently, interesting venues of research have been developed using the growth pattern of baleen plates. Baleen is formed continually and is worn out at the tip over years or decades; for species with particularly long plates such as right and bowhead whales, the length of a plate can represent up to 25 years of the life of an animal. This characteristic is being used to study stress-related factors affecting whales and their reproductive hormone levels over time, for example to document pregnancies [18, 19]. Thus there is the possibility that physiological stresses due to climate change and changing environmental conditions could be assessed in modern whale populations and compared to ancient specimens whose plates are stored in museum collections [19]. In combination with isotope readings along the plate, whale migration patterns could be determined, as could diet and the location of feeding grounds [20].

### Historical and cultural importance of baleen whales

#### Baleen in material culture

Mysticeti whales are ubiquitous around the world; there are currently 14 species of listed baleen whales (Table A in S1 File). Some species (sei, blue, fin, minke and humpback) have nearly worldwide distributions while others are adapted to specific ecosystems; for instance the Bryde’s whale inhabits the tropical and subtropical latitudes and the bowhead whale the northern circumpolar regions. While evidence of prehistoric whaling has been found around the globe [21, 22] (for example in Indonesia [23], Japan [24], Norway, Scotland and Iceland [25]), indigenous whale hunting has been of particular importance for the Arctic and North Pacific where traditional hunting of large whales has been practiced for over 4000 years and stranded whales have likely been harvested for thousands of years [26–29]. Prehistorically, baleen was collected along with the bones, meat and blubber of a whale. A number of Arctic cultures fashioned baleen into fishing and hunting implements (buckets, ice scoops, bows, sled runners, fish lines, lashings, nets, snares), clothing (boot insulation) and other artefacts [30–32], and today baleen is incorporated into Alaskan Iñupiat and Yup’ik artwork and fashioned into baskets. In Japan some baleen artefacts date back to the 8^th^ c. [33], and more recently baleen has been used to make tea trays, decorative elements such as the wrapping of swords and puppet springs [31].

Elsewhere, there is little known about traditions of baleen use, even though whale remains and artefacts made of whale bones have been found in many archaeological sites (for instance in Northern Europe [34–37], South America [38] and New Zealand [39, 40]). In the United Kingdom for example, only two archaeological finds of baleen are known [41]. This is likely due to the poorer preservation of keratinous tissues in warm and temperate climates compared to bone. Bone is tightly packed with collagenous fibrils and is highly mineralized (50–70%). The inorganic component of baleen is much lower than in bone; in sei whales, for example, the hydroxyapatite content has been estimated at only 4.5% [42]. Without the protective mineral component, baleen, like other keratinous tissues, would be susceptible to biodegradation [41].

Baleen becomes more conspicuous in material culture associated with commercial whaling by Europeans and later by Americans. Due to its tough and flexible properties, the material was used for a wide range of objects and acquired a high commercial value. As early as the 13^th^ c. A.D., baleen was used in Europe in the construction of armor and tourney equipment [41]. During the 18^th^ and 19^th^ c. A.D., when exploitation of baleen was at its maximum, the plastic material was made into sheets, strips or rods and used in items of clothing (stays, corsets and hoop skirts) [43], and in the production of objects such as umbrella stays, eyeglass frames, combs, boxes, etc. [31, 41, 43].

#### Exploitation of baleen whales

Most archaeological sites containing whale bones are found in the northern hemisphere and right and bowhead whales have been considered to have been the most targeted species. It is usually associated with prehistoric and historic Iñupiat, Thule and Inuit sites [44–46]; for instance bowhead whale bones are found in abundance in Alaska and central Canada, where prehistoric Inuit used them as house supports [44, 47]. It was hunted in the Eastern Arctic, and together with the humpback whale, was taken in Greenland by early Greenlandic whalers [21]. Archaeological sites along the Pacific Northwest Coast (where whale use has been demonstrated for at least 4,000 years [48, 49]) have yielded only a small proportion of right whale bones, while gray whale and humpback whale remains constitute almost the entirety of the bone assemblages of archaeological sites such as the Toquaht sites on Vancouver Island [48], the Ozette site in Washington State [50] and the Par-Tee site on the Oregon Coast [51]. The gray whale, whose range extends to the Chukchi Sea and Wrangel Island, was also hunted by Chukchi and Inuit in Russia [21] and in Japan [33]. With the invention of net whaling in the second half of the 17^th^ c. in Japan, catching of the right whale was made easier and whaling intensified and extended to humpback, Bryde’s, minke, fin and blue whales [33].

In pre-modern whaling, right, bowhead and gray whales were favored by European and American whalers as they were slower swimmers than other whales, produced large amounts of oil and could be killed with hand-thrown harpoons and lances ([52], p600). For example, the right whale was named so because it inhabits coastal waters, is a slower swimmer than other mysticetes and floats when dead—i.e., it was the “right” whale to catch ([52], p77). When Basque whaling started in the 11^th^ c. (French Basque country in the Bay of Biscay) and the 12^th^ c. (Spanish Basque country) the North Atlantic right whale was mainly hunted during its winter southern migration [53]. Later on whaling by westerners moved to the North Atlantic, starting with the English Channel in the 14^th^ c., Iceland in the 15^th^ c., Norway and Svalbard in the 16^th^ c and Greenland in the 17^th^ c. At that point large numbers of bowhead whales were caught in addition to the right whale [43]. European whaling expanded to the coasts of Newfoundland and Labrador in the 16^th^ c. [54, 55] where recent genetic studies on 16^th^ and 17^th^ c. bones have shown that the Basques caught mostly bowhead and a few humpback whales and not the right whale as initially thought [56, 57].

**Fig 2:**
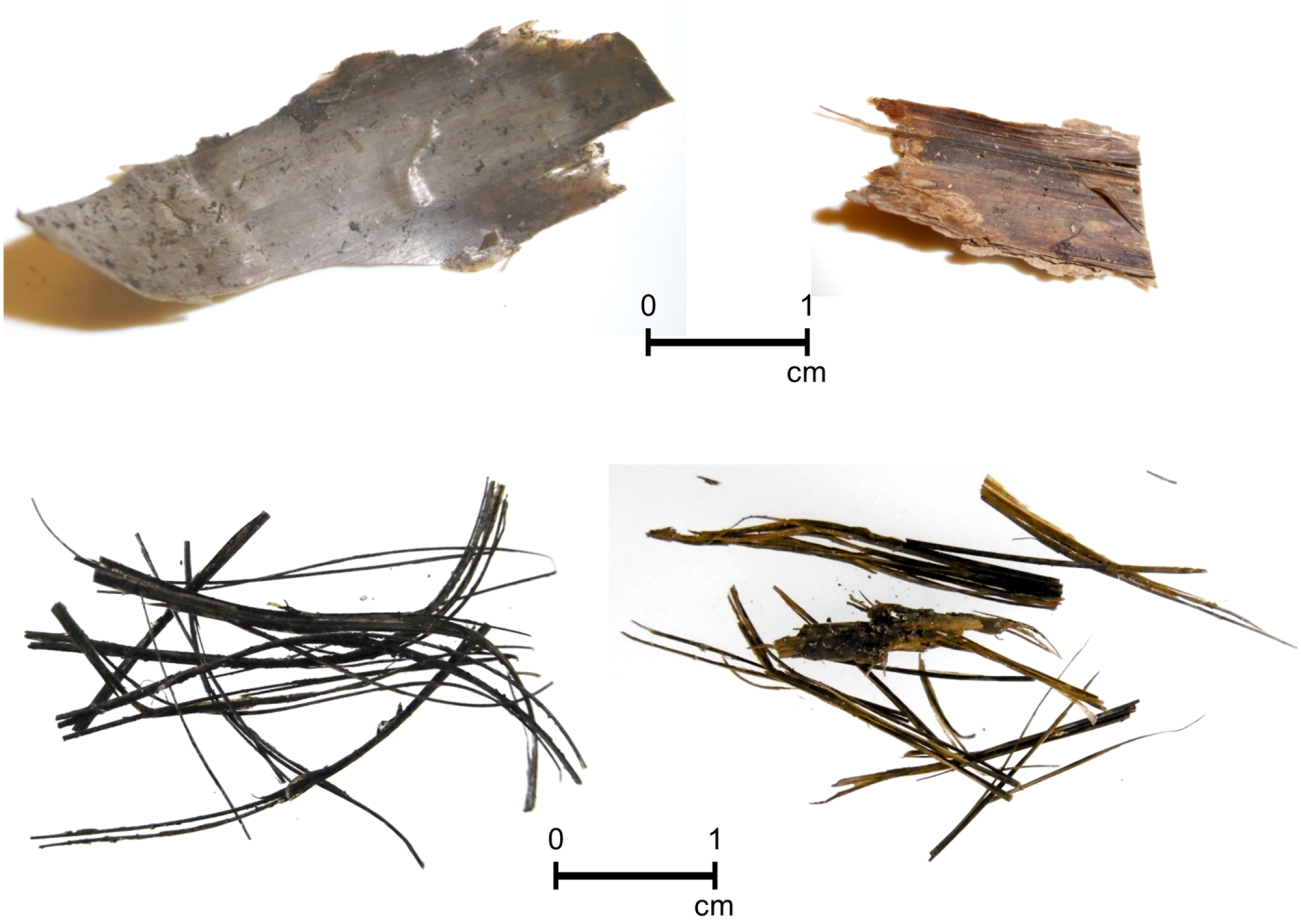
Archaeological baleen from Labrador. Left: Strip and bristles from Avayalik-1 (JaDb-10, 49A). Right: Strip and bristles from Johannes Point (IbCq-1).

It was not until the advent of power boats and harpoon guns ([52], p416) that the fast rorquals (*Balaenoptera* sp) were caught as well. The intensive whaling activity that ensued in the 19^th^ and 20^th^ c. have caused most species to be severely depleted and there is currently a debate about pre-commercial whaling population sizes. A better appreciation of the species hunted and the extent of whaling in specific geographical areas are needed for conservation purposes. In addition, knowledge about the species used prehistorically for their baleen could contribute to our understanding of various groups’ cultural traditions. Firm identification of the species represented in baleen specimens and baleen artefacts is a step towards these goals. Baleen plates vary in shape, size, thickness and color depending on the species (Table B in S1 File), but these features can be difficult to recognize once the material has been worked, cut or has degraded [31, 41]. Upon drying, baleen shrinks and becomes brittle (Fig 2), and the tubules become detached from the outer layer. Since the morphological characteristics of baleen have been altered in ancient baleen we turned to biomolecular techniques to develop a method of identifying the species of whale from which the ancient baleen was originally harvested.

### Peptide mass fingerprinting (PMF) of baleen

To address problems of species identification of unidentified museum specimens (resulting for example from outdated, inaccurate, absent or erroneous curatorial records), DNA barcoding was developed as a taxonomic tool. This method uses one or multiple short genetic sequences to identify a specimen or part of an animal and if possible connect it to a reference species, and has been used to describe new taxa [58–61]. However concerns have been raised about the accuracy of this method of analysis, such as species delimitation and availability of controlled reference specimens [61–64]. The idea that DNA barcoding can be used to describe new organisms and assess biodiversity based on a single locus has been criticized [65]. The proteomics equivalent to DNA barcoding, peptide mass fingerprinting (PMF), is different in that it targets whole proteins and relies on multiple peptide markers instead of a short DNA sequence. As in DNA barcoding though, it relies on well-characterized specimens for reference materials and has taxonomic limitations (identification up to the genus level for example). But PMF can be used to select ancient specimens for liquid chromatography-tandem mass spectrometry (LC–MS/MS) that will produce sufficient peptide sequences to establish molecular phylogeny (based on one type of protein, e.g., collagen [66]). Peptide mass fingerprinting is a rapid, accurate and efficient method for species identification of ancient artefacts, it requires little material and can provide results on processed and degraded material [41, 67, 68]. It has recently been used to study marine mammal remains, using collagen peptides in bones for species identification and to screen whale remains for biomolecule preservation in ancient deposits [36, 38, 69]. Species belonging to the same genus (*Balaenoptera* sp.) have PMFs distinct enough to identify those species in archaeological bones [36], which raises the interesting possibility that similar results could be obtained on baleen.

**Fig 3:**
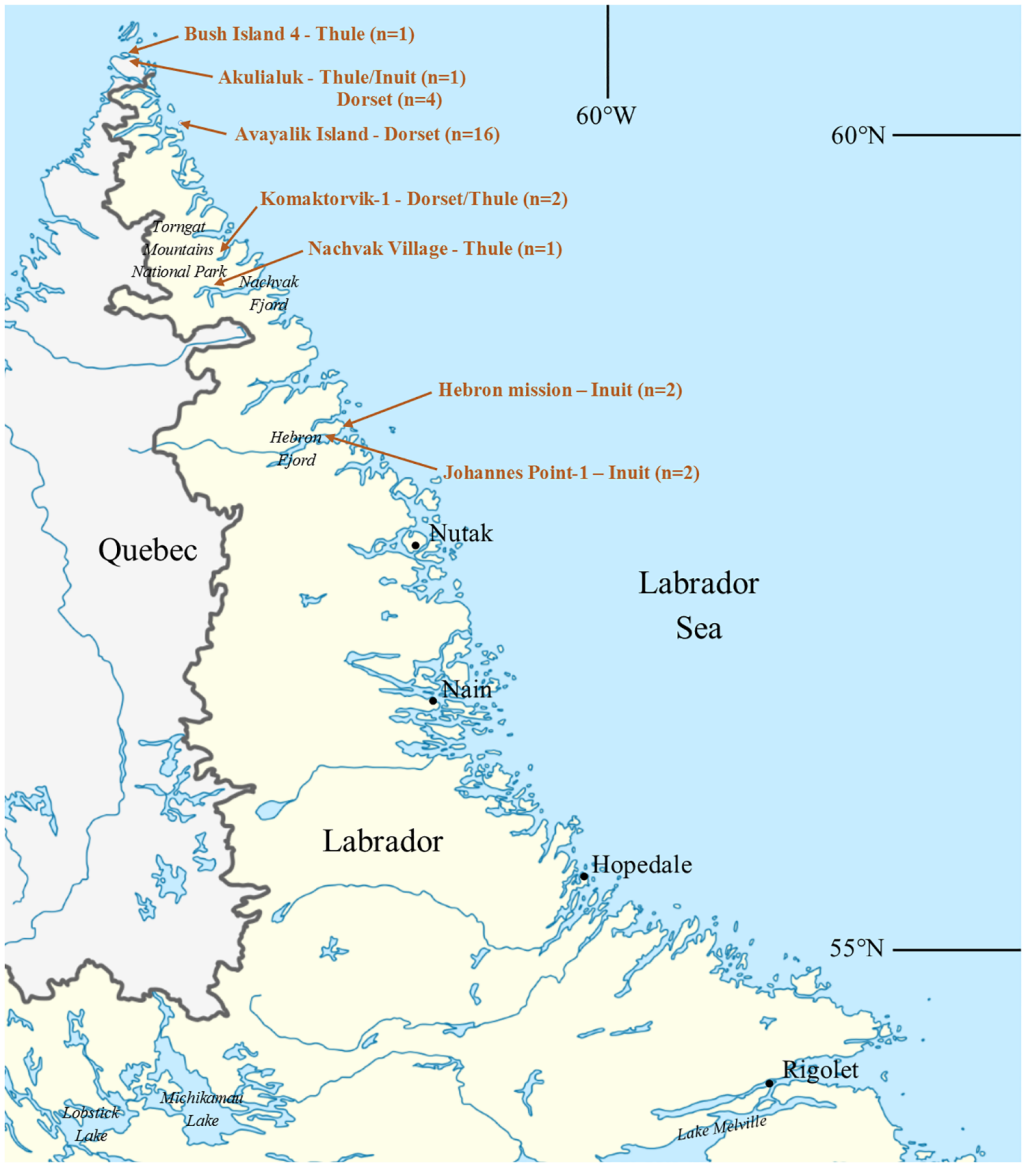
Map of Labrador, Canada, modified from NordNordWest [CC BY-SA 3.0 (http://creativecommons.org/licenses/by-sa/3.0)], via Wikimedia Commons, and showing the location of the archaeological sites where baleen was found, as shown in Fitzhugh, 1980 [71].

Baleen is made of alpha-keratin proteins, the same type found in hair and nail; the tubules are made of flattened keratinised cells packed with intermediate filaments (α-keratins) in the length of the tubule and surrounded by a protein matrix. A preliminary study on baleen [70] found that the typical type I (acidic Ha) and type II (basic Hb) keratin proteins dominated the peptidic profile, and that this profile presented distinct peaks at similar positions to animal fibers. Based on variations in amino acid sequences across taxa, each PMF is however representative of the analyzed genus (and sometimes species). Here we use 27 specimens from the Smithsonian Institution’s Museum of Natural History collection (acquired between 1879 to 1988) to determine markers of identification for 10 species of mysticeti whales (North Pacific right, North Atlantic right, bowhead, Bryde’s, sei, blue, common minke, fin, humpback and gray whales). The Southern right whale (*Eubalaena australis*), the Pigmy right whale (*Caperea marginata*, belonging to its own distinct family), Omura’s whale (*Balaenoptera omurai*) and the Antarctic minke whale (*Balaenoptera bonaerensis*) were not included in this study due to the lack of availability of suitable specimens. Species assignment of each specimen sampled was based on accession records, morphological characteristics of the plate and in some cases the whole rack; multiple specimens were sampled for each species whenever possible. The methodology was applied to 29 archaeological baleen samples obtained from multiple sites in Labrador, Canada (Fig 3) spanning up to 1500 years of aboriginal use of whales, including both stranded and hunted individuals. Taxonomic identifications based on the formerly described morphological characteristics of baleen [41] were impossible due to the fragmentary state of the remains.

## Materials and methods

### Sample collection

Reference materials from *Eubalaena japonica* (one specimen, n = 1), *Eubalaena glacialis* (n = 2), *Balaena mysticetus* (n = 3), *Balaenoptera brydei/edeni* (n = 4), *Balaenoptera borealis* (n = 4), *Balaenoptera musculus* (n = 3), *Balaenoptera acutorostrata* (n = 1), *Balaenoptera physalus* (n = 3), *Megaptera novaeangliae* (n = 3), *Eschrichtius robustus* (n = 3), were obtained from the Marine Mammals department at the Smithsonian Institution (Table 1). A permit for destructive sampling was obtained from the Division of Mammals of the National Museum of Natural History (NMNH). Samples were collected by drilling at the base (the side of the plates embedded in the jaw) where there was maximal thickness. The surfaces of the plates were cleaned with water and the first drilled layer was discarded. The second layer was then collected from one hole (two for thin plates) and placed into plastic vials. Another recent specimen (date and species unknown) was also obtained from AgResearch (Jeff Plowman, Proteins and Biomaterials group), Christchurch, New Zealand (unregistered specimen, no permit required for sampling) by scratching the surface with a razor blade (S2 File).

**Table 1:** Details of specimens sampled and quantity of baleen analysed.

Accession #	Scientific name	Common name	Collection date	Collection location	Mass tested (mg)
Balaenidae family					
USNM 339990	*Eubalaena japonica*	Pacific right whale	22 August 1961	Collected by the Whale Research Institute, south of Kodiak Island, Gulf of Alaska	16
USNM 504257	*Eubalaena glacialis*	Atlantic right whale	11 May 1975	Collected from a beached whale carcass by JG. Mead (SI) on Monomoy Is, Massachusetts	19
USNM 504343	*Eubalaena glacialis*	Atlantic right whale	15 April 1976	Collected from a whale carcass by JG. Mead (SI) off Duck Harbor, Wellfleet, Massachusetts	17
USNM 571336	*Balaena mysticetus*	Bowhead whale	1988	Collected by Richard.Lambertsen, Arctic Ocean, Alaska	11
USNM 571337	*Balaena mysticetus*	Bowhead whale	1988	Collected by Richard.Lambertsen, Arctic Ocean, Alaska	15
USNM 571338	*Balaena mysticetus*	Bowhead whale	May 1987	Collected by Richard.Lambertsen, Barrow, Arctic Ocean, Alaska	9
Balaenopteridae family					
USNM 239307	*Balaenoptera brydei/edeni*	Bryde’s whale	18 March 1923	Collected by AJ Poole & CE. Mirquet from a whale carcass at Walnut point, Virginia	15
USNM 504074	*Balaenoptera brydei/edeni*	Bryde’s whale	30 May 1974	Collected by Barry Peers from a stranding carcass, Tarpon Springs Gulf of Mexico, Florida	16
USNM 504688	*Balaenoptera brydei/edeni*	Bryde’s whale	5 January 1975	Collected by TJ. Mcintyre (SI), during Japanese whaling, East of South Island of New Zealand	14
USNM 504689	*Balaenoptera brydei/edeni*	Bryde’s whale	5 January 1975	Collected by TJ. Mcintyre (SI), during Japanese whaling, East of South Island of New Zealand	23
USNM 486174	*Balaenoptera borealis*	Sei whale	10 December 1972	Collected from a whale carcass by JG. Mead (SI) on north end of Cape Island, Charleston, South Carolina	4
USNM 504244	*Balaenoptera borealis*	Sei whale	16 April 1975	Collected from a whale carcass by JG. Mead (SI) at Corolla, North Carolina	7
USNM 504706	*Balaenoptera borealis*	Sei whale	14 February 1975	Collected by TJ. Mcintyre (SI), during Japanese whaling, in Bransfield Strait, Antarctica, South Atlantic Ocean	11
USNM 504998	*Balaenoptera borealis*	Sei whale	12 July 1974	Collected at La Costa De Buen Hombre, Dominic Republic	18
269541	*Balaenoptera musculus*	Blue whale	No record		12
USNM 302977	*Balaenoptera musculus*	Blue whale	NA	Gift from Russian whaling ship	14
USNM 504996	*Balaenoptera musculus*	Blue whale	1970s	Collected by ED. Mitchell ‘s team from a stranded whale, West coast of Newfoundland	11
USNM 239305	*Balaenoptera acutorostrata*	Minke whale	July 1922	Collected by EP. Walker (SI), Pearl Island, Pribilof Islands, Alaska	17
USNM 275769	*Balaenoptera physalus*	Fin whale	30 August 1947	Collected by RM. Gilmore at Eureka, Del Norte, California	4
USNM 504258	*Balaenoptera physalus*	Fin whale	27 May 1975	Collected from a whale carcass by JG. Mead (SI) in Brigantine, New Jersey	9
USNM 504712	*Balaenoptera physalus*	Fin whale	13 March 1975	Collected by TJ. Mcintyre (SI), during Japanese whaling, Antarctica, South Pacific Ocean	6
USNM 16252	*Megaptera novaeangliae*	Humpback whale	12 April 1879	Collected by EE. Small & Capt. NE. Atwood (SI) from a stranded whale in Provincetown, Massachusetts	10
USNM 267999	*Megaptera novaeangliae*	Humpback whale	24 August 1938	Baleen collected from a seized whale from the whaling ship Frango by Lt. TR Midtlyng in Western Australia	16
USNM 504216	*Megaptera novaeangliae*	Humpback whale	29 February 1975	Collected from a whale carcass by JG. Mead (SI) at Virginia Beach, Virginia	16
Eschrichtiidae family					
USNM 504999	*Eschrichtius robustus*	Gray whale	15 January 1968	Collected by RL. Delong at Del Monte whaling station at Richmond, California	11
USNM 572613	*Eschrichtius robustus*	Gray whale	3 January 1967	Collected by FM. Greenwell (SI) from the Del Monte Fishing Company, San Francisco, California	8
USNM 572614	*Eschrichtius robustus*	Gray whale	Jan. 1967	Collected by FM. Greenwell (SI) possibly from Point Reyes, California	12
Unknown					
339	Unknown		No record	Likely collected in New Zealand	10

Archaeological baleen was sampled from plate strips (five samples) and bristles (24 samples) (details in Table A in S3 File), recovered from houses and middens excavated in 1977–1978 from sites attributed to different cultures (Fig 3): Dorset, ca. 1500 B.P. to 600 B.P. [71] (20 samples), Dorset/Thule (three samples), Thule, ca. A.D. 1500 [72, 73] (two samples), Thule/Inuit (one sample), and historic period Labrador Inuit, post A.D. 1800 (four samples). Since cataloguing in 1978 the samples have been stored in sealed plastic vials at the Arctic Studies Center, NMNH (no permits were required for the described study).

### Protein extraction

The fresh and archaeological samples of baleen were washed with water twice, (archaeological samples were sonicated for 10 seconds to remove dirt) followed by ethanol and left to dry. The samples were then cut into small pieces with a scissor cleaned with ethanol. Proteins were extracted by overnight shaking in a 0.5 ml solution of 8M urea, 50 mM Tris and 50 mM TCEP at pH 8.4. An aliquot of 100 μL supernatant was alkylated for 45 min in the dark with 400 mM of iodoacetamide for a final concentration of 40 mM. The samples were dialysed overnight with 100 mM ammonium bicarbonate in 2 kDa molecular weight cut-off (MWCO) dialysis units (Slide-A-Lyzer^™^ MINI Dialysis Devices by Thermo Scientist). The samples were then digested with 0.5 μg of trypsin, for 18 h at 37°C, dried down in the morning and resuspended in 100 μL of 0.1% trifluoroacetic acid (TFA) before solid phase extraction with 3M Empore^™^ Octadecyl C18 extraction disk (Supelco, Bellefonte PA, USA), cut into 2 x 2 mm pieces. The proteins were extracted by shaking for 3 hours, the Empore cuts washed with 0.1% formic acid and proteins eluted with a solution of 75%/25% acetonitrile/formic acid, dried down and resuspended in 10 μL of 0.1% trifluoroacetic acid. The samples were spotted on AnchorChip^™^ target (Bruker) as previously described [70].

### Peptide mass fingerprinting by MALDI-TOF-MS

Analyses were carried out with an Ultraflex^™^ III mass spectrometer (Bruker), in positive reflector mode using a Nd:YAG laser operating at 355 nm. Spectra were acquired using flexControl 3.0 (Bruker) on a mass range of 700–3,500 Da with an accumulation of 500 shots on the standards and 1000 shots on the samples. The calibration standard (Bruker) was prepared according to the manufacturer’s instructions for instrument calibration and consisted of angiotensin I, ACTH clip(1–17), ACTH clip (18–39) and ACTH clip(7–38) peptides.

### Data analysis

The spectra were processed with mMass 5.5.0 (http://www.mmass.org/) after conversion of the raw files with flexanalysis 3.3 (Bruker). Spectra were smoothed with Gaussian filter (width 0.3 *m/z*) and internally recalibrated using the peptides identified in Table 2, for an error after calibration of ≤ 0.02 Da.

**Table 2:** Sequences used for recalibration, with calculated *m/z* (C indicates Carbamidomethylation of the cysteine) and matched species: (i) Mysticeti species where peaks are observed with ≤ 0.02 Da error; and (ii) species matched by sequence homology using Blast (https://blast.ncbi.nlm.nih.gov/).

Sequence	*m/z*	Protein references^a^	Species (i)	Other matches (ii)
GITGGFGSR	851.44	Hb1, Hb6	All	shrew, mole, squirrel^c^
WQFYQNR	1041.49	Hb1, Hb3, Hb5, Hb6	All	> 10 species
DVEEWFTR	1081.50	Ha1, Ha3	All	goat, sheep, antelope^d^
LGLDIEIATYR	1263.70	Hb1, Hb5, Hb6	All	> 10 species
LNVEVDAAPTEDLNR	1655.82	Ha1, Ha3, Ha6	All	-
TVNALEIELQAQHSMR	1839.94	Ha6	All	> 10 species
TVHALEVELQAQHNLR	1857.99	Ha1, Ha3	All but bowhead whale	Yangtze dolphin^e^
SDLEANSEALIQEIDFLR	2063.03	Hb1, Hb3, Hb6	All	> 10 species
SQQQDPLVCPNYQSYFR	2129.97	Ha1, Ha6	All	sperm whale^f^
VPYISSVPCAPAPQLSTQIR^b^	2184.15	Ha6	Balaenidae, minke and gray whales	-
VEAQLAEIR	1028.57	Ha1, Ha3	Balaenopteridae and gray whale	> 10 species
YSSQLAQIQGLIGNVEAQLSEIR^b^	2517.33	Ha6	Balaenopteridae and gray whale	-
YSSQLAQIQGLISNVEAQLSEIR^b^	2547.34	De novo	Bryde’s whales	-
YTSQLAQIQCLISNVEAQLSEIR^b^	2664.37	De novo	Balaenidae	-
APYISSVPCAPAPQLSTQIR^b^	2156.12	De novo	Bryde’s and sei whales	-

^a^Accession numbers in NCBI are: XP_007177676.1 keratin, type I cuticular Ha1-like [*B*. *acutorostrata scammoni*], XP_007177675.1 keratin, type I cuticular Ha3-I [*B*. *acutorostrata scammoni*], XP_007177677.1 keratin, type I cuticular Ha6 [*B*. *acutorostrata scammoni*], XP_007179861.1 keratin, type II microfibrillar, component 7C-like (Hb1) [*B*. *acutorostrata scammoni*], XP_007174324.1 keratin, type II cuticular Hb3-like, partial [*B*. *acutorostrata scammoni*], XP_007179462.1 keratin, type II cuticular Hb5 [*B*. *acutorostrata scammoni*], XP_007179463.1 keratin, type II cuticular Hb6 [*B*. *acutorostrata scammoni*].

^b^Sequences verified by MALDI-TOF-MS/MS analysis (spectra shown in S4 File).

^c^thirteen-lined ground squirrel (*Ictidomys tridecemlineatus*), common or Eurasian shrew (*Sorex araneus*), star-nosed mole (*Condylura cristata*);

^d^domestic goat (*Capra hircus*), domestic sheep (*Ovis aries*), Tibetan antelope or chiru (*Pantholops hodgsonii*);

^e^Yangtze dolphin (*Lipotes vexillifer*);

^f^sperm whale (*Physeter catodon*).

## Results

### Markers of identification in fresh baleen

**Fig 4:**
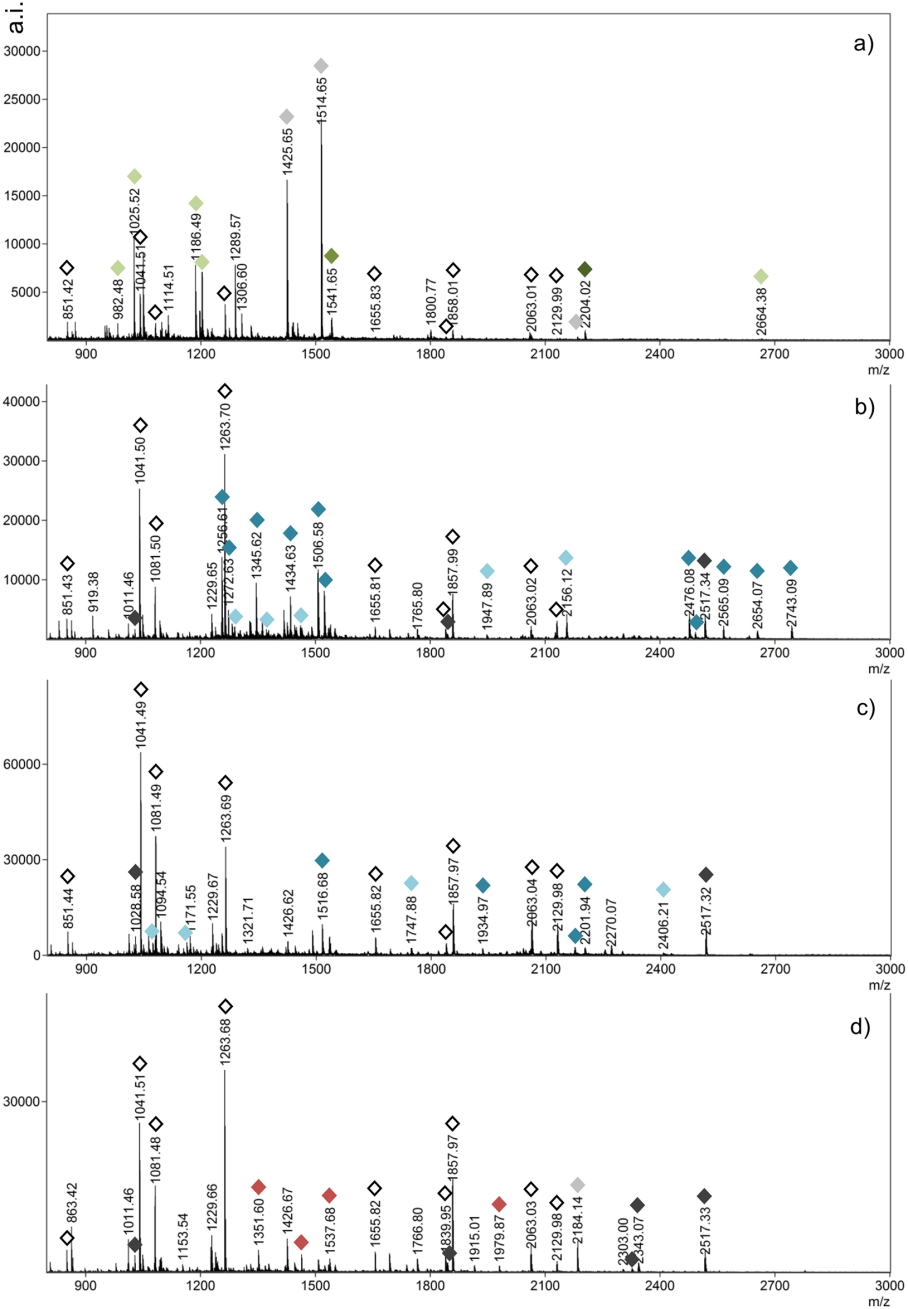
Mass spectra of four baleen specimens with main markers indicated, see Table 3 for colour references a) North Atlantic right whale (*E*. *glacialis*) 504257; b) sei whale (*B*. *borealis*) 504998; c) humpback whale (*M*. *novaeangliae*) 267999 and d) gray whale (*E*. *robustus*) 572613.

The species analyzed here have common peptides (Table 2 and in Fig 4 indicated with white-filled diamonds). The sequences of these peptides were previously identified [70] and have matches in the few publically available keratin sequences from the minke whale (Table 2). In particular, the peptide LNVEVDAAPTEDLNR appears to be specific to all Mysticeti whales only (Blast search against all organisms, timestamp December 2016). A few additional sequences were determined manually from MALDI-TOF-MS/MS spectra (Table 2 and S4 File). These sequences were used to re-calibrate internally the spectra for an error of less than 0.02 Da after calibration. Peaks that fell within this error tolerance are indicated in Table 3.

**Table 3:** Main diagnostic markers identified by peptide mass fingerprinting in Balaenidae (North Pacific (NP) right, North Atlantic (NA) right and bowhead whales), Balaenopteridae (Bryde’s, sei, blue, minke, fin and humpback whales), and Eschrichtiidae (gray whale). In gray and black are peaks found across families (in gray: all families and in black: Balaenopteridae and Eschrichtiidae), in green are balaenidae-only peaks (darker shade of green represents the right whales, and darkest green is for species-specific peaks), in blue are balaenopteridae-only peaks (darker shade of blue represents species-specific peaks), and in red are the eschrichtiidae-specific peaks. The—sign indicates the presence of a peak with relative intensity < 1%.

*m/z*	NP Right*	NA Right	Bowhead	NA Bryde	SP Bryde (504689)	SP Bryde (504688)	Sei	Blue	Minke*	Fin	Humpback	Gray
2184.15												
1425.65												
1514.64												
1028.57												
2517.33												
1844.73						-						
2326.04						-						-
2343.07				-		-						
982.49	-											
1025.52												
1186.49/1203.50												
2664.37		-										
1541.64												
1843.95												
2204.02												
1282.63/1298.64												
1371.62												
1460.63												
1947.90					-							
2156.12												
1532.58/1549.62												
2547.34												
1256.61/1272.63												
1345.62												
1434.63												
1506.58/1523.62												
2476.08/2492.07												
2565.09												
2654.07												
2743.09												
1779.82												
1818.89												
1950.99												
1073.52												
1162.52												
1747.88												
2406.22											-**	
1516.68												
1934.97												
2174.90												
2201.95												
1351.60												
1463.71												
1537.68												
1979.87												

*Species for which only one specimen was available.

**Observed in the specimen 267999 from Western Australia (might indicate a local form of humpback whale) and in the unidentified New Zealand sample B339 (S2 File) as a low intensity peak

Balaenidae (North Atlantic and North Pacific right whales, and bowhead whale) have common peaks that differentiate them from the other baleen species (in light green Table 3). In addition, the right whales are characterized by a peak at *m/z* 1541.64 while the presence in the bowhead whale of a peak at *m/z* 1843.95 (and absence of *m/z* 1857.99, Table 2) allows distinction from the right whales. A peak at *m/z* 2204.02 was found in the North Atlantic right whale only (Fig 4a).

The Balaenopteridae (Bryde’s, sei, blue, minke, fin and humpback whales) and Eschrichtiidae (gray whale) species are characterized by one common peak, at *m/z* 1028.57 (sequence in Table 2). The peak at *m/z* 2517.33 (sequence in Table 2) also indicates a species from these families, but was absent in two of the Bryde’s whale specimens tested (see below). Bryde’s and sei whales, two species genetically close, have a few common peaks (light blue Table 3), but sei whale has the highest occurrence of specific peaks of all species (dark blue Table 3 and Fig 4b). Blue and minke whales have one common peak at *m/z* 1779.82 but can be differentiated by the presence of the peaks at *m/z* 1950.99 and *m/z* 2184.15 (sequence in Table 2) in the minke whale, and *m/z* 1818.89 in the blue whale. Fin and humpback whales have four common peaks (light blue Table 3), but are differentiated from each other by the peak at *m/z* 1425.65 in the fin whale, and four specific peaks in the humpback whale (dark blue in Table 3 and Fig 4c). Finally gray whale can also easily be differentiated by the presence of four specific peaks (in red Table 3 and Fig 4d). Reference mass spectra are given in S4 File for each species (raw data in S1 Data).

### Identification of the Bryde’s whales

**Fig 5:**
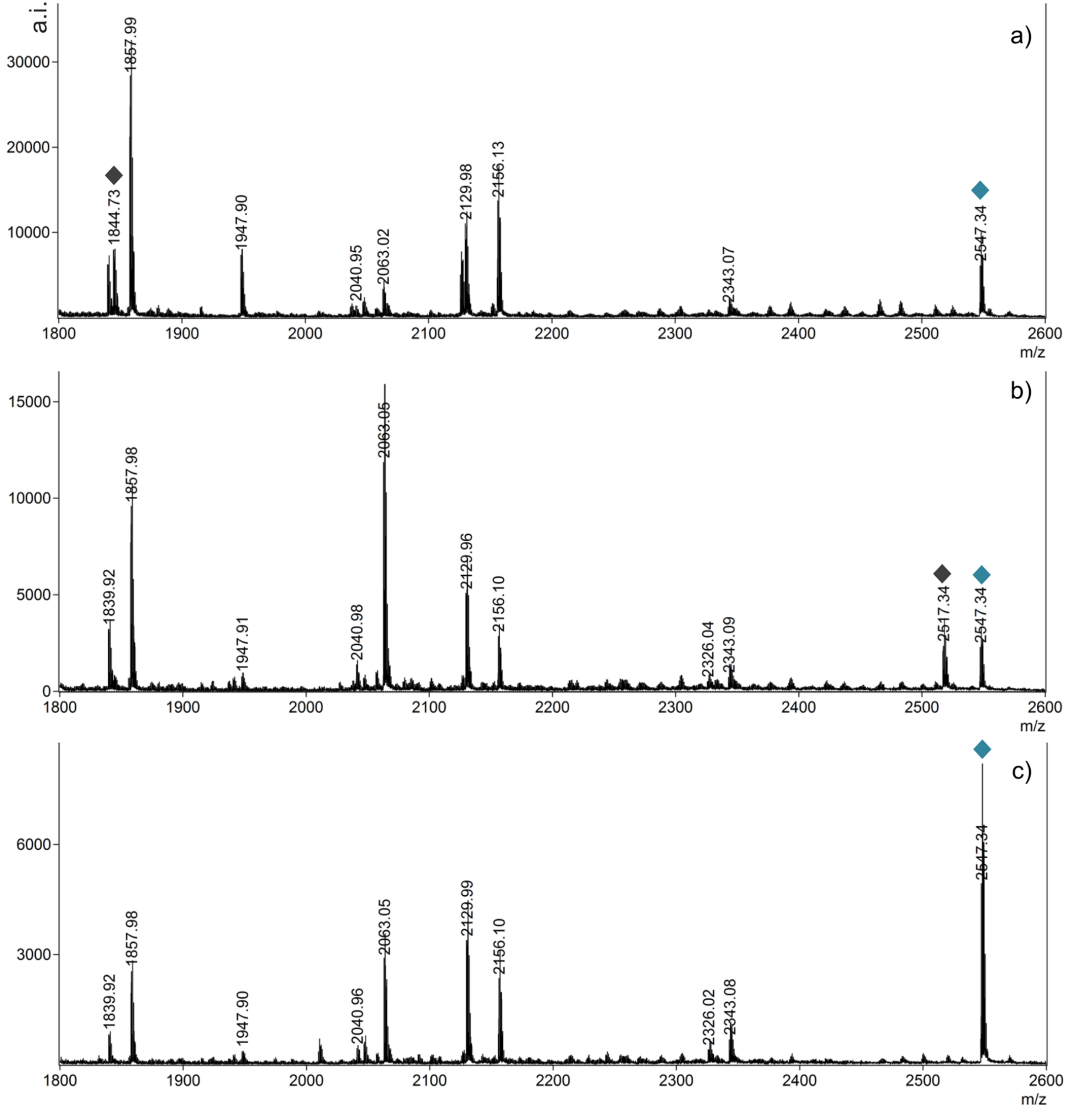
Mass spectra between *m/z* 1800–2600 of the Bryde’s whales (*B*. *brydei*) showing subspecies markers a) North Atlantic specimen 504074; b) South Pacific specimen 504688; c) South Pacific specimen 504689.

The Bryde’s whale samples analyzed here come from two different geographical areas, the North Atlantic Ocean for specimens 239307 and 504074 and the South Pacific Ocean for specimens 504688 and 504689. They are characterized by the specific peaks at *m/z* 1532.58, 1549.62 and 2547.34 (sequence in Table 2). However, the North Atlantic specimens (Fig 5a) present a peak at *m/z* 1844.73 (not to be confounded with the bowhead peak at *m/z* 1843.95), which is visible in very low intensity in the South Pacific specimen 504688 (Fig 5b) and totally absent from the South Pacific specimen 504689 (Fig 5c). In addition, peptide *m/z* 2517.33 is present in specimen 504688 but not in 504689 or 504074.

### Archaeological samples

**Fig 6:**
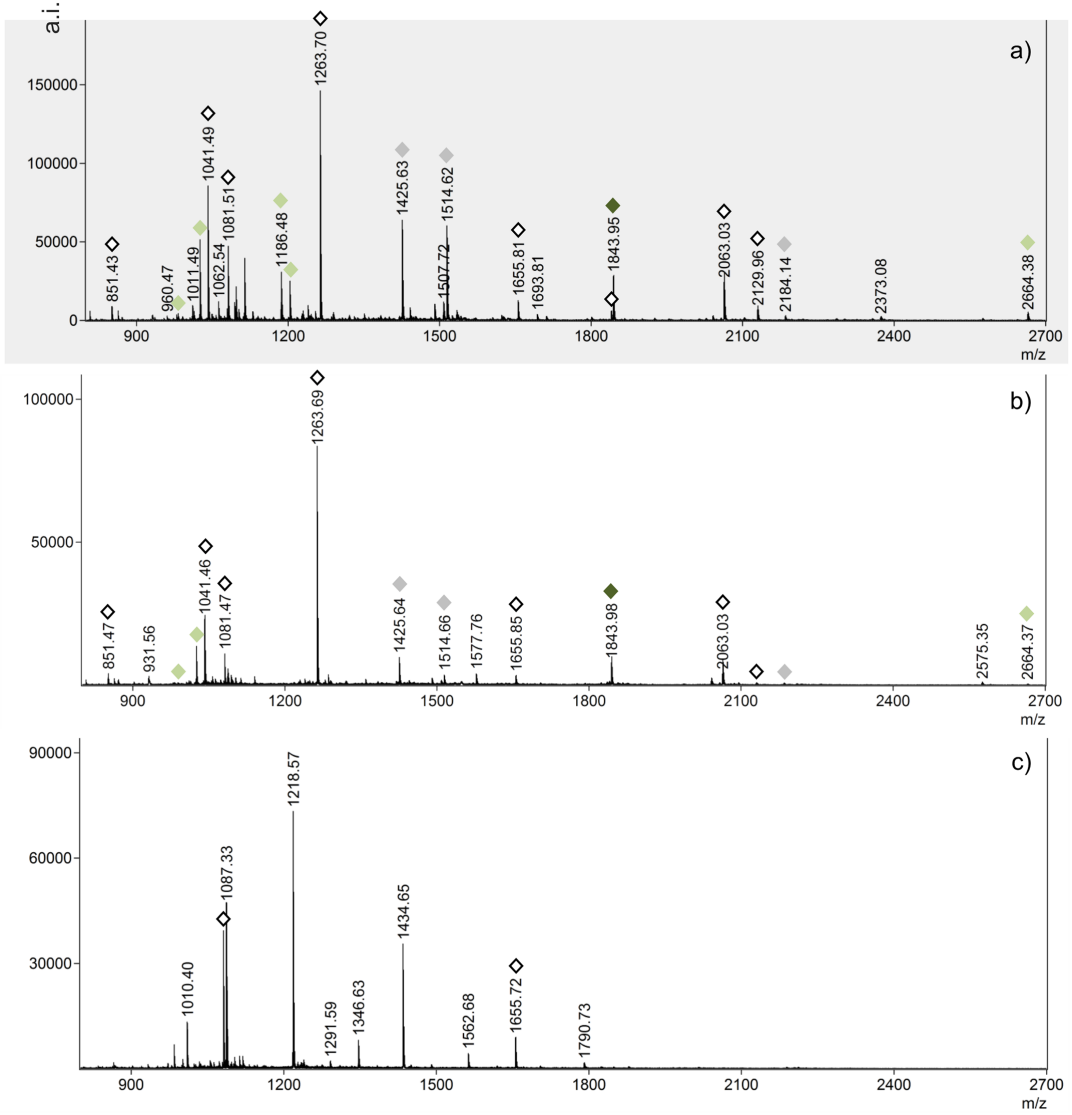
PMF of a) bowhead whale sample 571338; b) archaeological baleen, Dorset (Ava18); c) degraded archaeological baleen, Inuit (Joh49).

All but two archaeological samples were matched to the bowhead whale reference PMF (Fig 6a) based on the presence of peaks at *m/z* 1025.52, 1425.65, 1514.64, 2664.37 (Balaenidae) and *m/z* 1843.95 (species-specific); a sample from Avayalik-1 is shown in Fig 6b (Ava18) and all other samples in S3 File (Table A and Figures A1 to A29). The samples from the Inuit site Johannes Point in Hebron cannot be matched to any species (Fig 6c). The profiles show patterns of degradation with loss of common peptides and new peaks likely originating from modifications to the polypeptide chains. While peaks at 1081 and 1655 (Fig 6c) are indicative of baleen, diagnostic peptides are absent, instead showing peaks at *m/z* 1218, 1791 and 1434. The same pattern of degradation seen at Johannes Point is reproduced in the Dorset Avayalik-1 samples 16 and 33 (Figures A4 and A14 in S3 File), in these samples however, the presence of the *m/z* 1025.5 peptide points to either the right or bowhead whale, while the presence of a *m/z* 1844.1 peak, albeit at very low intensity, indicates a likely match to bowhead whale.

## Discussion

### Phylogeny of baleen whales

The divergence date of the Balaenidae from the other families has been estimated at 28 Ma [74, 75]. The phylogeny of the Balaenidae is well established with *B*. *mysticetus* (bowhead) split estimated between 5 and 10 Ma [75, 76] and much more recent divergence time for the Eubaleana (right whales) genus (less than a million year [75]). Our results are consistent with the ancient split of the Balaenidae family as right and bowhead whales have PMFs distinctive of the other baleen whales. While analyses of bone collagen have not been able to differentiate between bowhead and right whales using the ZooMS method [69], the analysis of baleen demonstrates a specific peak for the bowhead whale at *m/z* 1844, as well as distinct peaks for the right whale. These results allow distinction of bowhead and right whale in baleen, as well as indicating a possible separation of North Pacific and North Atlantic right whales. However, as only one specimen of the North Pacific right whale was available for testing, the absence of the peak at *m/z* 2204 in that species will have to be confirmed. These unexpected results also indicate the need to test baleen from the Southern Hemisphere right whale (*E*. *australis*) that has been accepted as a distinct species phylogenetically closer to the North Pacific right whale [77]. In modern times, whalers depleted right whales in the Northern Hemisphere causing the industry to pursue the right whale in the Southern Hemisphere. The possibility of distinguishing among the different species of right whales could be of great use to provenance baleen in historic objects. The pigmy right whale (*C*. *marginata*), so-called because of morphological similitudes with the right whale, was not hunted commercially [78]; molecular data have now placed it with balaenopterids and eschrichtiids rather than with balaenids [79].

The phylogenetic relationships of the Eschrichtiidae and Balaenopteridae (last common ancestor estimated at 12–13 Ma [74, 75]) have been the subject of many studies as the morphological and molecular evidence have brought up different results [80]. Many studies have found fin and humpback whales to be sister taxa [75, 80–82] (split time 7 Ma [75]), as well as Bryde’s and sei whales [75, 80–83] (split time 3 Ma [75]). The high occurrence of common peptides between Bryde’s and sei whales and between fin and humpback whales are in line with the established relationships. Relationships are less clear for minke and blue whales: a study on Y chromosomes found minke whales to be close to the fin-humpback clade and the blue whale branching from the other Eschrichtiidae/Balaenopteridae species [80]. One study placed the blue whale in a sister group of the fin-humpback clade based on mtDNA but found blue and fin to be sister taxa based on concatenated nuclear genes with minke as the closest relative [74]. Another placed the blue whale in a sister clade of the Bryde’s/sei group and minke whale branching out [82]. In Gatesy et al. (2013) [84], blue whale is placed next to the Bryde’s/sei group as well as in McGowen et al. (2009) [75] which estimates a split date of 9 Ma. Our analyses so far found that minke and blue whales cannot be closely associated to any other species based on keratin markers from PMF; complete sequencing of the keratins would be needed to determine the phylogenetic placement of these species. In addition, only one specimen of minke whale was successfully tested. A second specimen of the common minke whale yielded no useable data (very degraded profile) and no Antarctic minke whale were tested here.

McGowen et al. [75] finds Eschrichtiidae (gray whale) to be most closely related to the fin-humpback clade with a divergence time from within the Balaenopteridae [81] at about 9 Ma [75]. Our PMF data indicate that keratin sequences are quite divergent between these three species, as no common diagnostic peptide markers were found among them.

### Bryde’s whales

The taxonomy of the Bryde’s whales, first described in South Africa in 1913 [85], has not been fully established due to the subsequent recognition of a morphologically similar species. Named *B*. *edeni* the new species, under which name all Bryde’s whales have been classified, was first fully described after a Singapore specimen in 1950 [86]. Recently *B*. *brydei* and *B*. *edeni* have been recognized as separate species based on mtDNA [87, 88]. Sasaki refers to pelagic Bryde’s whales (western North Pacific and eastern Indian Ocean) as belonging to *B*. *brydei* while specimens collected in Hong Kong, Japan and Australia are *B*. *edeni*, morphologically smaller than the *B*. *brydei* specimen. Several forms of *B*. *brydei* have been described, such as the offshore and inshore South African populations [89]. Bryde’s whales also occur in the Pacific (Peru), the Atlantic (Brazil) and in New Zealand (*B*. *brydei* type) [90]. More recently, a smaller type of Bryde’s whale was identified through specimens located in the western Pacific and eastern Indian Oceans. The specimens were first referred as pigmy Bryde’s whales [91], but in 2003 they were recognized as belonging to a separate species named *B*. *omurai* that lies outside the Bryde’s/sei clade [88].

Our analyses are consistent with the identification of two different species (*B*. *brydei* and *B*. *edeni*), or multiple sub-species. The lack of known morphological differentiation between the plates of *B*. *brydei* and *B*. *edeni* makes it difficult to associate any of our samples to a particular species. It is worth noting, however, that the North Atlantic specimens have thinner and denser bristles with a lighter coloration than the South Pacific specimens. In this regard, the three profiles observed for the Bryde’s whales should be considered characteristic of any species or sub-species of Bryde’s whales independent of their geographical origin. More specimens are needed to validate these results, in particular for the Southern Hemisphere samples that both yield different profiles. In addition, specimens of the Omura’s whale should be tested to determine if its baleen profile is indeed different from the Bryde’s and sei whales profiles and fit current genetic data.

### Baleen preservation

The vast majority of the modern baleen sampled from plates yielded good results, with only three specimens (not included here) failing to give useable PMFs. Those results are encouraging for they suggest that is it possible to identify the species of whale represented in historical baleen artefacts; the oldest specimen, a humpback whale from 1879, had a profile consistent with the younger humpback specimens. The archaeological samples also yielded good PMFs, with the exception of the samples from the Johannes Point Inuit site dating to the 17-19^th^ centuries.

As mentioned earlier, keratinous tissues are generally more susceptible to biodegradation in archaeological sites than are bones; such rapid decomposition has been observed in all hard tissues made of alpha-keratins (horn, hoof, nail, claw) and beta-keratins (tortoiseshell) [41]. The protein analysis of degraded samples is often translated by the loss of diagnostic peaks: as the polypeptidic chain is degraded into smaller fragments through hydrolysis, PMFs are characterized by an increase of peptides of smaller molecular weight and decrease or complete loss of peptides at higher *m/z*. Further chemical degradation is usually observed with the deamidation of glutamine and asparagine, a frequent modification in archaeological hair for instance [68] and observed in the archaeological baleen samples to a small degree (Table B in S3 File). Deamidation, however, only results in small measurable shifts in peaks. The observation in the Johannes Point samples of unknown peaks, not observed in other baleen whale species and, to our knowledge not observed in hair samples or human contamination, likely results from other undetermined modifications to the peptides (such as truncated or semi-tryptic peptides).

The cold environment of the Arctic is undoubtedly a crucial factor for the preservation of baleen remains. Johannes Point, the southernmost of the sites sampled, is built on well-drained sands, lacks permafrost and has a protected southern exposure, meaning higher seasonal ambient temperatures and the greatest exposure to environmental degradation of the archaeological series. The samples from this site came from test pits that produced poorly-preserved faunal remains. The advanced chemical degradation of the samples was not obvious through visual examination when compared to baleen bristles with good protein preservation (Fig 2); this indicates that the biomolecular information preserved in baleen and by extension in bones is at risk due to exposure to a warm climate.

### Archaeological significance

In the time period covered by this study, several species of whales would have been found in the Labrador waters: the blue, sei, right, bowhead, humpback, minke, fin and gray whales. The North Atlantic population of gray whale went extinct around the early 1700s [92] and the Northwestern Atlantic population of right whale seems to have already been decimated by the time Basque whaling began in Newfoundland and Labrador in the 16th c. [93]. The identification of bowhead in nearly all samples is not necessarily an indication of the sole use of bowhead for baleen supplies. Several reasons can explain the absence of other species: 1) the modest sample size of the samples (29 samples), 2) the possibility that samples originate from the same animal (there are a few samples that come from the same location, Table A in S3 File), 3) preferential degradation of baleen in some species (the hydroxyapatite content might vary from species to species [42] and baleen with low mineral content would be more susceptible to degradation), and 4) a biased representation of baleen in archaeological sites (baleen is rarely preserved in other sites with more exposure, lacking permafrost deposits or at lower latitudes).

However, the dominance of bowhead whale remains in samples from Saqqaq, Dorset, Thule, and historic Inuit sites is consistent with past studies and probably results from two factors: bowhead accessibility and its highly desirable products. During the Dorset period, seal and walrus were the principal quarry. Dorset people lacked the technology suitable for hunting large whales, including floats and other specialized open-water whaling technology, but their carving of whale bone to make sled runners and tool handles, and use of baleen lashings suggests a consistent pattern of scavenging materials from bowheads that died of natural causes and drifted ashore. Eighteenth century’s Moravian records document extensive use of ‘drift whales’ by Labrador Inuit [94] and we can expect Dorset people made similar use of buoyant bowheads. Furthermore, recent bowhead DNA evidence from Greenland Saqqaq sites dating to 4000 B.P. [29] shows this species was used by the first Paleo-Inuit arrivals from Alaska and the wider North Pacific region, where, we may expect, scavenging and, later, hunting of large whales probably originated during early/mid-Holocene times.

The Thule specialized in subsistence hunting large whales in open water [72]. Their pursuit of bowheads was possible because of their specialized harpoon, float, and boat technology. This slow-swimming species played a central role in their western Arctic-derived whaling adaptation. Whale meat, blubber, and skin provided vital nutrition to their communities and their dog teams. Whale bones were used as architectural elements in dwellings and were carved into tools and weapons, blubber was used in lamps to light house interiors and cook food, and baleen was used to make hunting, fishing, traveling and household implements. The social organization and ideology of a community centered around whale-boat crews and whales. None of the other large whales could be approached and killed as easily as bowheads. Its continued importance in later historic Alaska, Labrador and Greenland Inuit society can be attributed to the same factors: ease of capture and abundant raw materials and food resources. In the 1600s, trade between Europeans and northern Labrador Inuit communities was initiated and intensified in the 1700s [72]: baleen, sealskins, and down were exchanged for European-derived raw materials (hardwoods, metals) and manufactured commodities [73]. After the 1800s, however, large whales became rare in Labrador due to over-exploitation by European and American whalers, and smaller, less predictable catches were reported [72].

## Conclusion

The identification of baleen species used in ancient artefacts can help archaeologists better understand what species prehistoric groups hunted and how the resources they provided were utilized. Historical records about catches are not numerous and are often incomplete. In addition, the reliability of whale identifications based on visual reports can be questionable. For instance, it is unclear from historic sightings whether bowhead or right whale was caught by North Atlantic whalers. In addition, the bowhead was until recently also called Greenland right whale [37, 95], adding to the confusion as to which species was effectively hunted. As a result of these problems, using historical records to make estimates of the size of pre-European whaling whale stock numbers is fraught with problems.

Turning to biomolecular analysis of whale remains is one way to improve our knowledge of past whaling activity and will add important information about the prehistoric and historic availability of the Mysticeti whales. Peptide mass fingerprinting of baleen offers a new analytical tool to identify baleen specimens or baleen-made artefacts: sample sizes can be reduced to a few mg and baleen sourced from artefacts in both prehistoric and historic collections. We demonstrate here that identification is possible at the species and possibly at the sub-species level, with additional well-characterized specimens necessary to look for differences at the sub-species level. The data indicate a higher level of differentiation in baleen PMFs than in mammal hair. In hair for instance, only one peptide can be reliably used to differentiate sheep from goat, two species that belong to distinct genera [70]; here, species from the same genus have distinguishable profiles. The extent of the divergence of the whale keratins will however only be possible once sequence information for all baleen whales is known (number of alpha-keratin proteins, amino-acid sequences, intraspecies variations).

Peptide fingerprinting is an interesting alternative to DNA barcoding to identify taxa, and has many applications, from archaeology to wildlife forensics [61]. There is a wide range of keratinous tissues (hair, horn, feathers, tortoiseshell) and organisms on which the methodology can be applied to provide a fast and easy taxonomic identification that will complement or offer a substitute technique when degradation prevents identification by microscopy or DNA.

## Supporting information

S1 File Baleen whales. (PDF)

S2 File Baleen sample B339. (PDF)

S3 File Archaeological materials from Labrador. (PDF)

S4 File Mass spectra of reference materials. (PDF)

S1 Data MALDI-TOF raw files. (ZIP)
