# Supplementary material for: Molecular markers in keratins from Mysticeti whales for species identification of baleen in museum and archaeological collections
Source: PLoS One. 2017 Aug 30;12(8):e0183053. doi: 10.1371/journal.pone.0183053 (PMC5576650; doi:10.1371/journal.pone.0183053)
Supplement: S1 File — (PDF) [file pone.0183053.s001.pdf]

Supporting Information File S1

Baleen whales

Latest divergence dates for the baleen whales estimate that mysticetes appeared in the late Middle Eocene and diverged from odontocetes around 38.8 Ma [1]. Fossils are known from the late Eocene-early Oligocene (34-33 Ma) in Antarctica [2].

Taxonomy. According to the Society for Marine Mammalogy (Committee on Taxonomy. 2016. List of marine mammal species and subspecies. Society for Marine Mammalogy, www.marinemammalscience.org, consulted on 12th May 2016), there are 14 recognised species of baleen whales (suborder Mysticeti) (Table S1-1). They are grouped into four families [3]: the Balaenidae, the Neobalaenidae, the Eschrichtiidae and the Balaenopteridae (or rorquals). The Balaenidae (four species) include the northern right whales (North Atlantic right whale *Eubaleana glacialis* and North Pacific right whale *Eubaleana japonica*), and the southern right whale (*Eubaleana australis*) that have been recognised as three separate species of right whales [4, 5], and the bowhead whale (*Baleana mysticetus*). The Neobalaenidae and the Eschrichtiidae both have one species, respectively *Caperea marginata* (the pigmy right whale [6]) and *Eschrichtius robustus* (gray whale). Finally the Balaenopteridae (eight species) include *Balaenoptera acutorostrata* (common minke whale), *Balaenoptera bonaerensis* (Antartic minke whale recognised as a separate species since 2000 to differentiate the southern hemisphere minke whales), *Balaenoptera borealis* (sei whale), *Balaenoptera musculus* (blue whale), *Balaenoptera physalus* (fin whale), *Balaenoptera omurai* (Omura's whale, a recently described species [7, 8]), *Balaenoptera edeni* (Bryde's whale, whose taxonomy is uncertain and might be represented by several species and subspecies [9]) and *Megaptera novaeangliae* (humpback whale).

Table A: Current Mysticeti whales. Source: http://www.iucnredlist.org/search

Latin name	Common name	Geographic range
***Eubalaena japonica* [10]**	North Pacific right Whale	From the Sea of Okhotsk, the southeastern Bering Sea, the Aleutian Islands, and the northern Gulf of Alaska to the sea of Japan and California
***Eubalaena glacialis* [11]**	North Atlantic right whale	Western (from Florida to Gulf of St Lawrence) and Eastern North Atlantic (Azores to Iceland and Norway)
***Eubalaena australis* [12]**	Southern right whale	South hemisphere, circumpolar
***Balaena mysticetus* [13]**	bowhead whale	Arctic and Subarctic regions (USA, Canada, Russia, Japan, Greenland)
***Caperea marginata* [14]**	Pygmy Right Whale	South hemisphere, circumpolar, temperate waters
***Balaenoptera brydei* [15]**	Edeni-Bryde's whale	Pacific, Indian and Atlantic oceans, tropical to temperate
***Balaenoptera omurai* [16]**	Omura's whale	From Japan to Indonesia to the Solomon Islands
***Balaenoptera borealis* [17]**	sei whale	North Atlantic, North Pacific and Southern hemisphere, from tropical to subpolar latitudes
***Balaenoptera musculus* [18]**	blue whale	All oceans and seas except the Arctic, Mediterranean, Okhotsk and Bering seas
***Balaenoptera acutorostrata* [19]**	Common minke whale	North Atlantic, North Pacific and Southern hemisphere, and in all latitudes
***Balaenoptera bonaerensis* [20]**	Antarctic minke whale	South hemisphere, circumpolar
***Balaenoptera physalus* [21]**	fin whale	North Atlantic, North Pacific and Southern hemisphere, temperate to subpolar waters
***Megaptera novaeangliae* [22]**	humpback whale	North Atlantic, North Pacific and Southern hemisphere, and in all latitudes
***Eschrichtius robustus* [23]**	gray whale	North Pacific (Arctic sea to Mexico, Russia), and North Atlantic (now extinct)

Table B: Plate morphology for each species

Latin name	Common name	Plate morphology	References
Eubalaena japonica	North Pacific right Whale	No information available. See *E. glacialis*	
Eubalaena glacialis	North Atlantic right whale	The rack can reach up to 3 m in length and has 200 to 270 or 250-390 brown to black laminae (made of major + minor plates) on each side. The plates are very long and narrow, with plate length and width reaching a maximum of 1.8 m to 2.5 m and 25 cm. The bristles (dark brown/black) are very fine and long, and present in high density.	[24, 25]
Eubalaena australis	Southern right whale	No information available. See *E. glacialis*	
Balaena mysticetus	bowhead whale	The rack can reach 5 m in length and has 250 to 350 laminae on each side; the plates are dark brown to black. The plates are very thin, long and narrow; they can reach up to 4 m in length. Bristles are very long and have a similar dark brown (to a reddish-brown or dark orange) or black coloration.	[24-26]
Caperea marginata	Pygmy Right Whale	The rack is under one meter in length with a little over 200 laminae on each side (215-230); the plates are dark yellow. The plates' length and width reach a maximum of 50-70 cm and 7-12 cm. Bristles are dark yellow to dark gray/brown.	[24, 25]
Balaenoptera brydei	Edeni-Bryde's whale	Bryde's whale's racks have 285 to 350 laminae on each side; the plates are dark gray. The plates are short and wide (about 0.5 m in length). Bristles are long and thick, the density is high; their color is white to yellow and orange.	[25, 27]
Balaenoptera omurai	Omura's whale	The racks have about 200 laminae on each side; the colour of the plates varied with their position in the rack with posterior plates black, anterior plates yellowish-white and bi-coloured plates in between. The plates are short but broad (an average of 26 cm x 21 cm, length x width), with grayish-white bristles.	[8]
Balaenoptera borealis	sei whale	The racks have 219-402 laminae on each side; the plates are black, sometimes with a yellow stripe, short and wide (about 0.5 m in length). Bristles are long and thin, the density is high; their color is white.	[25, 27]
Balaenoptera musculus	blue whale	The racks have 270-395 laminae on each side; the plates are black, short and wide (about 0.5 m in length). Bristles are long and thick, the density is high; their color is black.	[25, 27]

Balaenoptera acutorostrata	Common minke whale	The racks are relatively short (about 1 m) but the plate density is high with 230 to 395 laminae on each side; the plates are white, yellow to orange, short and wide (about 0.3 m in length). Bristles are short, the density is low; their color is white.	[25, 27]
Balaenoptera bonaerensis	Antarctic minke whale	See *Balaenoptera acutorostrata*	
Balaenoptera physalus	fin whale	The racks have 350-400 laminae on each side; the plates are dark brown to yellow, short and wide (about 0.7 m in length, 0.3 in width). Bristles are long and thick, the density is low; their color is orange/yellow.	[25, 27]
Megaptera novaeangliae	humpback whale	The racks have an average of 270 to 400 laminae on each side; the plates are gray, brown or orange/yellow, short and wide (about 0.7 m in length). Bristles are long and thick, the density is low; their color is orange/yellow.	[25, 27]
Eschrichtius robustus	gray whale	The racks have 130-180 laminae on each side; the plates are off-white (or yellow), short and wide (up to 0.25 m in length). Bristles are very thick, the density is low; their color is off-white (or yellow).	[25]

REFERENCES

1. Marx FG, Fordyce RE. Baleen boom and bust: a synthesis of mysticete phylogeny, diversity and disparity. R Soc Open Sci. 2015;2(4). doi: 10.1098/rsos.140434.
2. Deméré TA, Berta A, McGowen MR. The Taxonomic and Evolutionary History of Fossil and Modern Balaenopteroid Mysticetes. J Mamm Evol. 2005;12(1):99-143. doi: 10.1007/s10914-005-6944-3.
3. Rychel AL, Reeder TW, Berta A. Phylogeny of mysticete whales based on mitochondrial and nuclear data. Mol Phylogenet Evol. 2004;32(3):892-901. doi: 10.1016/j.ympev.2004.02.020.
4. Gaines CA, Hare MP, Beck SE, Rosenbaum HC. Nuclear markers confirm taxonomic status and relationships among highly endangered and closely related right whale species. Proc R Soc Lond B Biol Sci. 2005;272(1562):533-42. doi: 10.1098/rspb.2004.2895. PubMed Central PMCID: PMCPMC1578701.
5. Rosenbaum HC, Brownell RL, Brown MW, Schaeff C, Portway V, White BN, et al. World-wide genetic differentiation of Eubalaena: questioning the number of right whale species. Mol Ecol. 2000;9(11):1793-802.
6. Fordyce RE, Marx FG. The pygmy right whale Caperea marginata: the last of the cetotheres. Proc R Soc Lond B Biol Sci. 2013;280(1753). doi: 10.1098/rspb.2012.2645. PubMed Central PMCID: PMCPMC3574355
7. Sasaki T, Nikaido M, Wada S, Yamada TK, Cao Y, Hasegawa M, et al. *Balaenoptera omurai* is a newly discovered baleen whale that represents an ancient evolutionary lineage. Mol Phylogenet Evol. 2006;41(1):40-52. doi: 10.1016/j.ympev.2006.03.032.
8. Wada S, Oishi M, Yamada TK. A newly discovered species of living baleen whale. Nature. 2003;426(6964):278-81. doi: 10.1038/nature02103.
9. Luksenburg JA, Henriquez A, Sangster G. Molecular and morphological evidence for the subspecific identity of Bryde's whales in the southern Caribbean. Mar Mamm Sci. 2015;31(4):1568-79. doi: 10.1111/mms.12236.
10. Reilly SB, Bannister JL, Best PB, Brown M, Brownell Jr. RL, Butterworth DS, et al. *Eubalaena japonica*. The IUCN Red List of Threatened Species. 2008. Available from: http://dx.doi.org/10.2305/IUCN.UK.2008.RLTS.T41711A10540463.en.
11. Reilly SB, Bannister JL, Best PB, Brown M, Brownell Jr. RL, Butterworth DS, et al. *Eubalaena glacialis*. The IUCN Red List of Threatened Species 2012. Available from: http://dx.doi.org/10.2305/IUCN.UK.2012.RLTS.T41712A17084065.en.
12. Reilly SB, Bannister JL, Best PB, Brown M, Brownell Jr. RL, Butterworth DS, et al. *Eubalaena australis*. The IUCN Red List of Threatened Species 2013. Available from: http://dx.doi.org/10.2305/IUCN.UK.2013-1.RLTS.T8153A44230386.en.
13. Reilly SB, Bannister JL, Best PB, Brown M, Brownell Jr. RL, Butterworth DS, et al. *Balaena mysticetus*. The IUCN Red List of Threatened Species 2012. Available from: http://dx.doi.org/10.2305/IUCN.UK.2012.RLTS.T2467A17879018.en.
14. Reilly SB, Bannister JL, Best PB, Brown M, Brownell Jr. RL, Butterworth DS, et al. *Caperea marginata*. The IUCN Red List of Threatened Species 2008. Available from: http://dx.doi.org/10.2305/IUCN.UK.2008.RLTS.T3778A10071743.en.
15. Reilly SB, Bannister JL, Best PB, Brown M, Brownell Jr. RL, Butterworth DS, et al. *Balaenoptera edeni*. The IUCN Red List of Threatened Species 2008. Available from: http://dx.doi.org/10.2305/IUCN.UK.2008.RLTS.T2476A9445502.en.

16. Reilly SB, Bannister JL, Best PB, Brown M, Brownell Jr. RL, Butterworth DS, et al. *Balaenoptera omurai*. The IUCN Red List of Threatened Species 2008. Available from: http://dx.doi.org/10.2305/IUCN.UK.2008.RLTS.T136623A4319390.en.
17. Reilly SB, Bannister JL, Best PB, Brown M, Brownell Jr. RL, Butterworth DS, et al. *Balaenoptera borealis*. The IUCN Red List of Threatened Species 2008. Available from: http://dx.doi.org/10.2305/IUCN.UK.2008.RLTS.T2475A9445100.en.
18. Reilly SB, Bannister JL, Best PB, Brown M, Brownell Jr. RL, Butterworth DS, et al. *Balaenoptera musculus*. The IUCN Red List of Threatened Species 2008. Available from: http://dx.doi.org/10.2305/IUCN.UK.2008.RLTS.T2477A9447146.en.
19. Reilly SB, Bannister JL, Best PB, Brown M, Brownell Jr. RL, Butterworth DS, et al. *Balaenoptera acutorostrata*. The IUCN Red List of Threatened Species 2008. Available from: http://dx.doi.org/10.2305/IUCN.UK.2008.RLTS.T2474A9444043.en.
20. Reilly SB, Bannister JL, Best PB, Brown M, Brownell Jr. RL, Butterworth DS, et al. *Balaenoptera bonaerensis*. The IUCN Red List of Threatened Species 2008. Available from: http://dx.doi.org/10.2305/IUCN.UK.2008.RLTS.T2480A9449324.en.
21. Reilly SB, Bannister JL, Best PB, Brown M, Brownell Jr. RL, Butterworth DS, et al. *Balaenoptera physalus*. The IUCN Red List of Threatened Species 2013. Available from: http://dx.doi.org/10.2305/IUCN.UK.2013-1.RLTS.T2478A44210520.en.
22. Reilly SB, Bannister JL, Best PB, Brown M, Brownell Jr. RL, Butterworth DS, et al. *Megaptera novaeangliae*. The IUCN Red List of Threatened Species 2008. Available from: http://dx.doi.org/10.2305/IUCN.UK.2008.RLTS.T13006A3405371.en.
23. Reilly SB, Bannister JL, Best PB, Brown M, Brownell Jr. RL, Butterworth DS, et al. *Eschrichtius robustus*. The IUCN Red List of Threatened Species 2008. Available from: http://dx.doi.org/10.2305/IUCN.UK.2008.RLTS.T8097A12885255.en.
24. Rice DW. Baleen. In: Perrin W, Wursig B, Thewissen JGM, editors. Encyclopedia of Marine Mammals, 2nd Edition. San Diego, CA: Academic Press; 2008. p. 78-80.
25. Young S. The comparative anatomy of baleen: evolutionary and ecological implications: San Diego State University; 2012.
26. Lambertsen RH, Rasmussen KJ, Lancaster WC, Hintz RJ. Functional Morphology of the Mouth of the Bowhead Whale and Its Implications for Conservation. Journal of Mammalogy. 2005;86(2):342-52. doi: 10.1644/ber-123.1.
27. Young S, Deméré TA, Ekdale EG, Berta A, Zellmer N. Morphometrics and Structure of Complete Baleen Racks in Gray Whales (Eschrichtius robustus) From the Eastern North Pacific Ocean. Anat Rec. 2015;298(4):703-19. doi: 10.1002/ar.23108.
