# Supplementary material for: Molecular markers in keratins from Mysticeti whales for species identification of baleen in museum and archaeological collections
Source: PLoS One. 2017 Aug 30;12(8):e0183053. doi: 10.1371/journal.pone.0183053 (PMC5576650; doi:10.1371/journal.pone.0183053)
Supplement: S2 File — (PDF) [file pone.0183053.s002.pdf]

Supporting Information File S2

Baleen sample B339

The baleen sample B339 (collection date and location unknown) was kept at AgResearch in New Zealand.

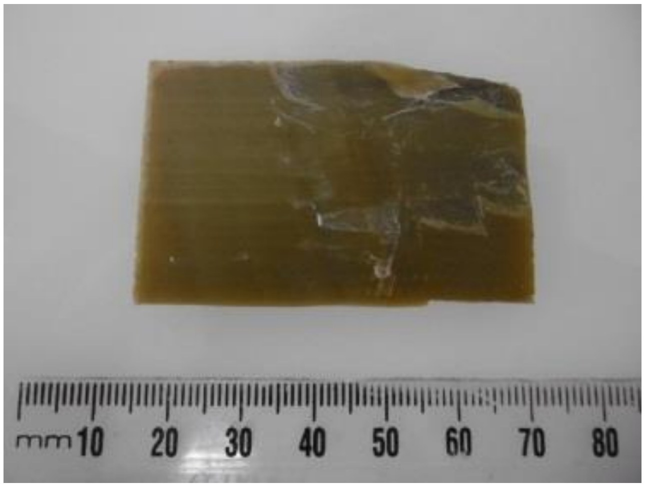

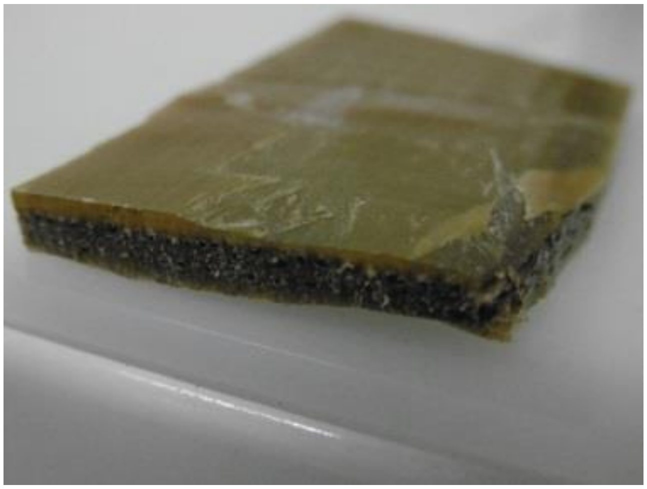

Photos from Caroline Solazzo

Blue, fin, sei, minke, humpback, Bryde's and southern right whales can be encountered in the New Zealand waters with humpback, Bryde's and right whales coming most closely to shores. Based on the presence in our unknown sample of intense peaks at *m/z* 1073.50, 1162.51 and 1747.90, we can readily identify the sample as belonging to the fin/humpback group (Fig S2). Fin whale is excluded based on the absence of the *m/z* 1425.65 while the presence of *m/z* 2174.91 and 2201.96 indicates that humpback whale is the most likely species. The presence of the peak *m/z* 2406.16 reported in the humpback whale (Table 1) was also observed in the humpback reference specimen from Western Australia, and might indicate a local form of humpback whale. However the peak is not intense and would require more specimens of humpback baleen to confirm this.

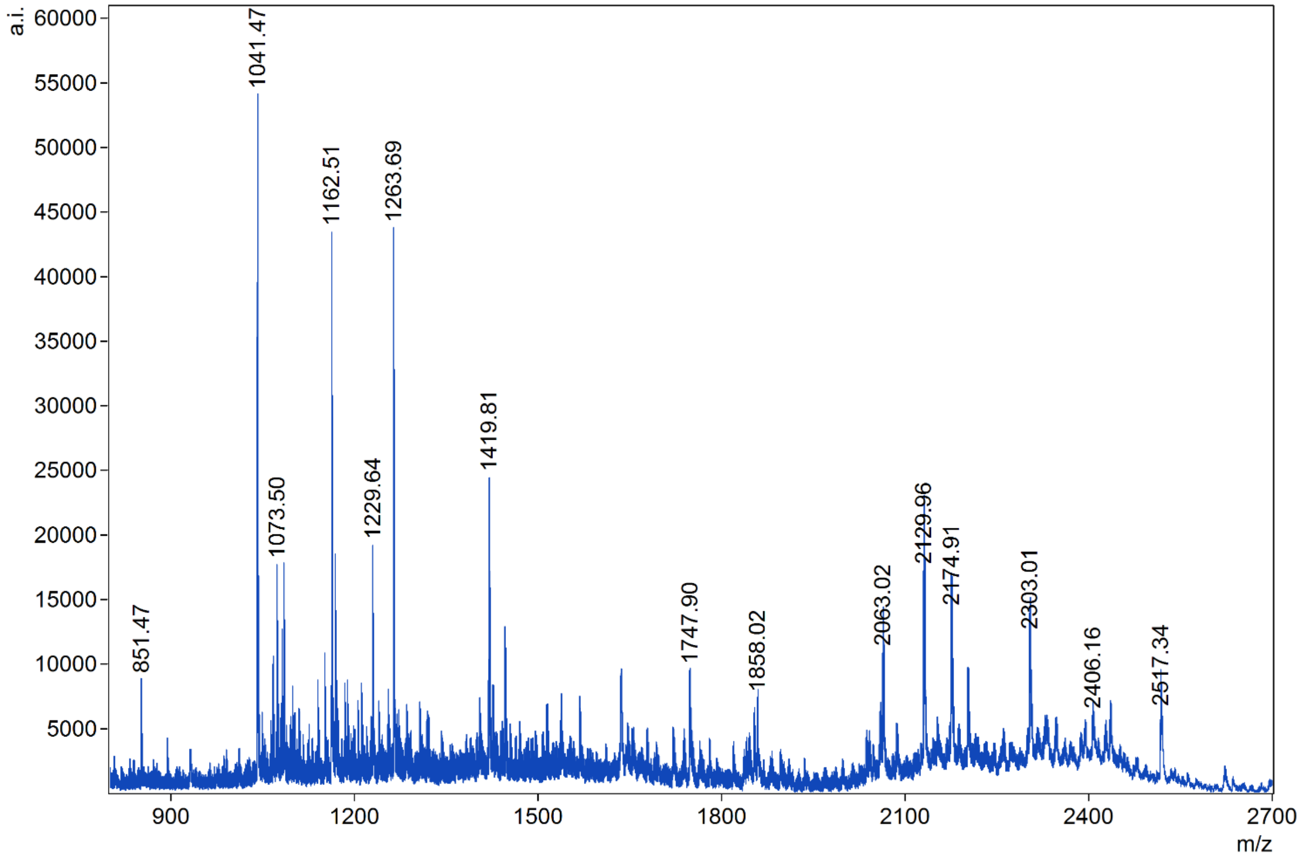

MS spectrum of B339
