# Supplementary material for: Molecular markers in keratins from Mysticeti whales for species identification of baleen in museum and archaeological collections
Source: PLoS One. 2017 Aug 30;12(8):e0183053. doi: 10.1371/journal.pone.0183053 (PMC5576650; doi:10.1371/journal.pone.0183053)
Supplement: S3 File — (PDF) [file pone.0183053.s003.pdf]

Supporting Information File S3

S3-A. Archaeological materials from Labrador p2-5

Table A: Baleen samples obtained from the Arctic Studies Center, Smithsonian Institution for species identification p5

Figures A1-A29: Peptide mass fingerprinting of archaeological samples p6-20

S3-B. Considerations on deamidation in the archaeological baleen p21

Figure B1: theoretical isotopic distribution of WQFYQNR (non-deamidated peptide) p23

Figure B2: peptide WQFYQNR in the reference bowhead whale sample 571338 and in an archaeological sample Avayalik_12, showing the changes in the shape of the peak p23

Table B: Average values of normalized IN0/IN1 ratio for selected peptides and archaeological sites p24

S3-A. Archaeological materials from Labrador

Bush Island. Bush Island is located north of Killinek Island at the northern tip of Labrador between Lenz and Grey Strait. Its archaeological sites were visited in 1967 by Patrick Plumet ([1] p 250) and by the Smithsonian's Torngat Archaeological Project (TAP) in 1977 and 1978. TAP's goals included documenting and testing Paleoeskimo (Pre-Dorset, Dorset) and Neoeskimo (Thule, Historic Inuit) archaeological sites to better understand regional culture history, technology, subsistence-settlement patterns, and site conservation conditions. Bush Island-4 (JcDe-10; Plumet's Killinek-6) consists of two sod dwellings and associated tent rings, caches, graves and other structures. House 1 was a large sod- and rock-walled winter dwelling with a 7-meter long entrance tunnel that contained well-preserved bone, wood, and a few European materials in a Thule and/or Historic Labrador Inuit context. A considerable amount of baleen was present together with a faunal collection predominatly of seal bones.

Avayalik Island (JaDb-10) [2] in Northern Labrador was investigated by TAP in 1977 and 1978. Avayalik is a small island 25 kms south of the tip of the Labrador Peninsula. The site is located in a small embayment that has attracted marine mammal hunters for more than 4000 years owing to a rich marine environment created by the mixing of waters from Ungava Bay, Hudson Strait, and Davis Strait. The 1200 square meter Avayalik-1 site [2, 3] was occupied by Middle and Late Dorset people. The upper level contained a Late Dorset house floor radiocarbon-dated to AD 1250-1420 (uncalibrated). This Late Dorset structure rested on a 30-cm thick frozen Middle Dorset midden dating to AD 420-660 (uncal.) with remarkable organic preservation. Faunal remains in the Middle Dorset midden consisted of walrus, seal, a fragment of a narwhal tusk, and several species of avifauna [4]. Although there is no evidence that Dorset people actively hunted large whales, artifacts made from whale bone are common at Avayalik and in other Dorset sites. In addition, the Avayalik midden contained large amounts of baleen and musk-ox hair cordage [3]. Whale bone and baleen, present in the site both as large strips and knotted strands, were probably scavenged from whale carcasses.

Akulialuk-1 (JcDe-6) [1, 4] , mapped and tested by TAP, is located on the northwestern side of Killinek Island facing Hudson Strait, is a large multicomponent site with Thule and historic Inuit sod houses resting on top of a deep Middle Dorset midden similar to that found at Avayalik. No Thule culture remains were present. Test excavations in the Dorset deposits produced a markedly different faunal assemblage from Avayalik consisting mainly of bird bones with few seal bones and no small or large whale bones. Baleen was present in the form of knotted strands. The houses, fauna, and location indicate a winter season occupation whose economy was based on the presence of an open-water winter polynia created by extreme tides and swift currents where Hudson Strait enters the Labrador Sea. These conditions produced an exceedingly rich avifauna habitat.

Nachvak [5] is the largest and most dramatic of all north Labrador Fjords, where whales and walrus used to be present in abundance. The Nachvak Village site (IgCx-3) has been radiocarbon dated to AD 1400 and contains both Thule and early historic Labrador Inuit occupations and the remains of sixteen sod houses [5-7]. TAP also tested sod houses at Ivitak Cove 1 (IgCw-1) which yielded a mixture of traditional Inuit implements along with 19th-early 20th c. European goods obtained from a nearby Hudson's Bay Company post. In 2003-4 excavations in Nachvak were conducted here by Peter Whitridge of Memorial University, who identified an early contact period in the late 17th-early 18th century at Nachvak Village [7, 8]. Whitridge's excavations at Ivitak confirmed earlier results and identified scattered remains of earlier Dorset occupations.

Komaktorvik-1 (IhCw-1) [9] has several sod houses and tent rings with occupations from Dorset, Thule, and Historic Inuit times. TAP identified most of the houses as 18th-19th century Inuit structures. However, House 7 was a Thule house excavated into a Late Dorset dwelling made of sod, stone, wood, and whale bones [10]. Like Nachvak Village, the site is mainly pre-contact Thule, but the presence of a small amount of European goods indicates some historical contact or exchange.

Hebron [11] was the name of a large Moravian station dating from 1830 to 1959 [5]. Before the Moravian mission arrived the aboriginal site, Hebron-1 (IbCp-17), was inhabited by both Dorset and by Inuit, as attested by "four prominent Inuit sod-house foundations situated on a slope overlooking Hebron Bay and Grubb Point to the south and east of the Moravian building complex" [11]. House-1 is the largest and is located 130 m from Houses 2, 3, and 4 which are clustered together along the edge of a former beach terrace south of the Mission House. Test-pits in all four houses were conducted during the TAP survey [6], which recovered 19th-century historic materials mixed together with Dorset Paleoeskimo artifacts. The baleen samples could have come from either context. Further excavations at Houses 1 and 2 were conducted in 1990, demonstrating an early to middle 18th century occupation [11].

Johannes Point-1 (IbCq-1) [9] is a pre-contact Thule occupation on the north side of Hebron Fjord 6 km west of Hebron-1 that has the visible remains of 14 sod houses excavated into the sandy bank of a prominent beach terrace. The site has traces of Pre-Dorset and Dorset occuaptions, but its visible houses date from the Thule period to the 19th c., with components including prehistoric Thule, early historic Inuit, and 19th-early 20th c. Inuit. No late 18th c. material was recovered. The baleen sampled from House 13 was described during excavation as 'poorly preserved' and probably part of a floor covering ([10], pp. 587-588). The soil was a peat-covered, dry sandy silt not condusive to organic preservation.

Table A (next page): Baleen samples obtained from the Arctic Studies Center, Smithsonian Institution for species identification.

Accession #	Analysis #	Collection location	Collection date	Period	Type	Mass tested (mg)	Identification
**Sample 12**	Ava12	Avayalik House 1, 2S/8E	1978	Dorset	Bristle	24	Bowhead
**Sample 13**	Ava13	Avayalik House 1, 0N/6E	1978	Dorset	Bristle	24	Bowhead
**Sample 14**	Ava14	Avayalik House 1, 2N/6E	1978	Dorset	Bristle	19	Bowhead
**Sample 16**	Ava16	Avayalik House 1, 2N/8E	1978	Dorset	Bristle	42	Likely bowhead
**Sample 18**	Ava18	Avayalik House 1, 2N/8E	1978	Dorset	Bristle	29	Bowhead
**Sample 19**	Ava19	Avayalik House 1, 2N/6E	1978	Dorset	Bristle	26	Bowhead
**Sample 21**	Ava21	Avayalik House 1, 2N/6E	1978	Dorset	Bristle	25	Bowhead
**Sample 22**	Ava22	Avayalik House 1, 0N/6E	1978	Dorset	Bristle	28	Bowhead
**Sample 25**	Ava25	Avayalik House 1, 2N/8E	1978	Dorset	Bristle	25	Bowhead
**Sample 26**	Ava26_B	Avayalik House 1, 2N/8E	1978	Dorset	Bristle	25	Bowhead
**Sample 26**	Ava26_S	Avayalik House 1, 2N/8E	1978	Dorset	Strip	21	Bowhead
**Sample 27**	Ava27	Avayalik House 1, 0N/6E	1978	Dorset	Bristle	28	Bowhead
**Sample 32**	Ava32	Avayalik House 1, 2N/8E	1978	Dorset	Bristle	23	Bowhead
**Sample 33**	Ava33	Avayalik House 1, 0N/8E	1978	Dorset	Bristle	23	Likely bowhead
**Sample 34**	Ava34	Avayalik House 1, 0N/8E	1978	Dorset	Bristle	27	Bowhead
**Sample 35**	Ava35	Avayalik House 1, 2N/2E	1978	Dorset	Bristle	25	Bowhead
**-**	HebH3	Hebron mission, House 3 midden TP 20-45 cm b.s.	1978	Inuit	Strip	20	Bowhead
**Sample 50**	Heb50	Hebron mission, House 3 midden TP 20-45 cm b.s.	1978	Inuit	Bristle	8	Bowhead
**Sample 49**	Joh49_B	Johannes Point-1 House 13 complex 12-28	1978	Inuit	Bristle	25	Unknown
**Sample 49**	Joh49_S	Johannes Point-1 House 13 complex 14-15	1978	Inuit	Strip	16	Unknown
**Sample 45**	Kom45_B	Komaktorvik-1 House 7, 2N/4W, 2N/2W, tunnel deposit	1978	Dorset/ Thule	Bristle	25	Bowhead
**Sample 45**	Kom45_S	Komaktorvik-1 House 7, 2N/4W, 2N/2W, tunnel deposit	1978	Dorset/ Thule	Strip	23	Bowhead
**-**	BushH1A	Bush Island-4 House 1 A mkw TP 8-16-78	1978	Thule	Strip	20	Bowhead
**Sample 52**	Nach52	Nachvak village House 9	1977	Thule	Bristle	20	Bowhead
**Sample 37**	Aku37	Akulialuk A, House 4 rvc	1978	Dorset	Bristle	24	Bowhead
**Sample 43**	Aku43	Akulialuk A, House 4 old midden	1978	Dorset	Bristle	20	Bowhead
**Sample 54**	Aku54	Akulialuk A, House 1 mkw	1978	Thule/ Inuit	Bristle	19	Bowhead
**Sample 55**	Aku55	Akulialuk A, House 4 rvc	1978	Dorset	Bristle	25	Bowhead
**Sample 57**	Aku57	Akulialuk A, House 4 rvc	1978	Dorset	Bristle	19	Bowhead

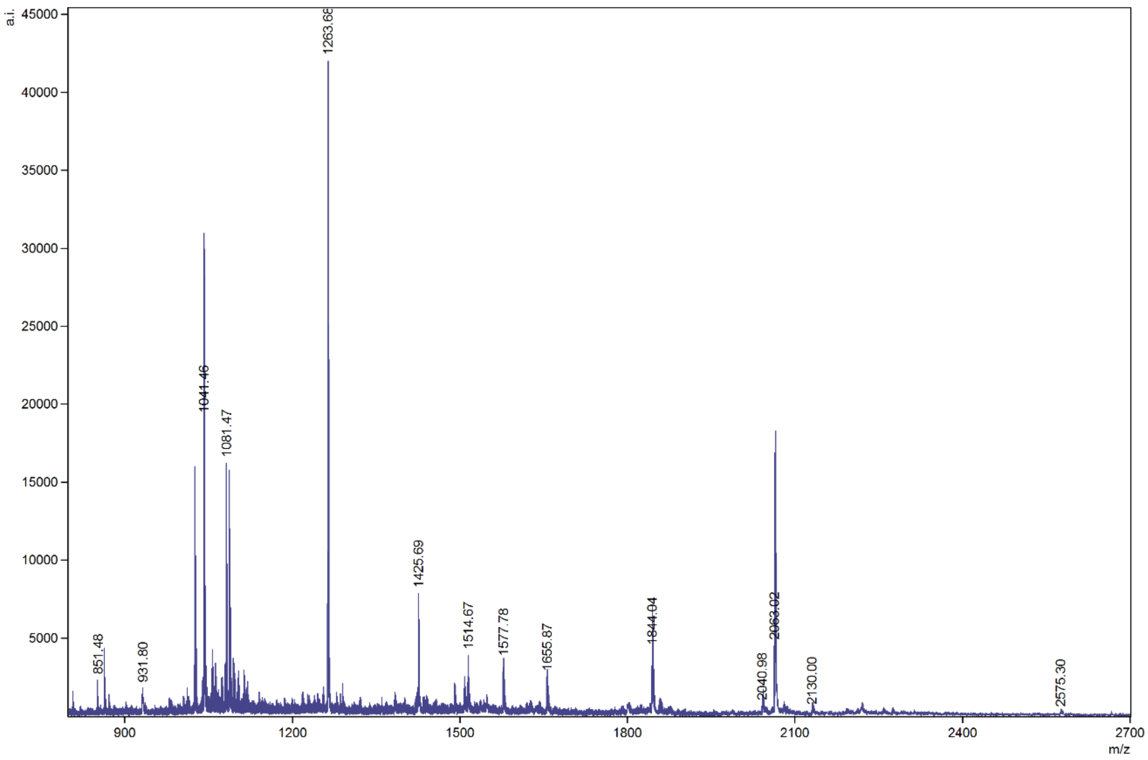

Figure A1: MS spectrum of Ava12 (Dorset)

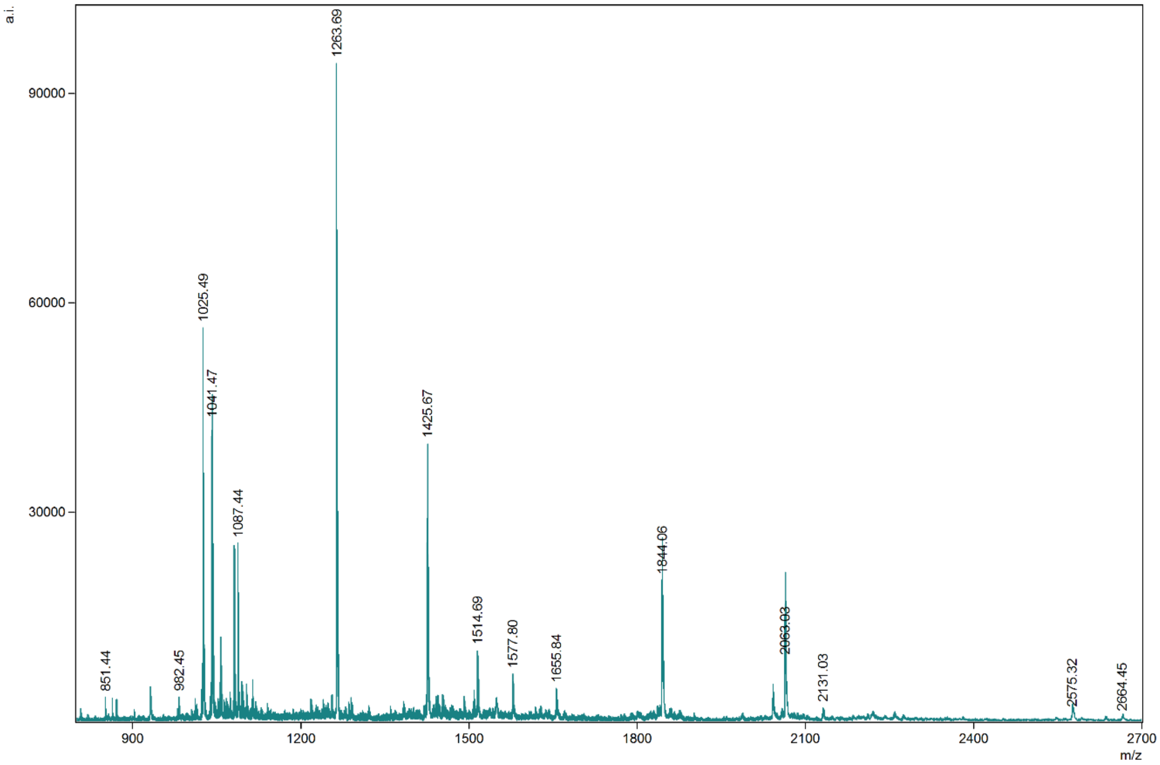

Figure A2: MS spectrum of Ava13 (Dorset)

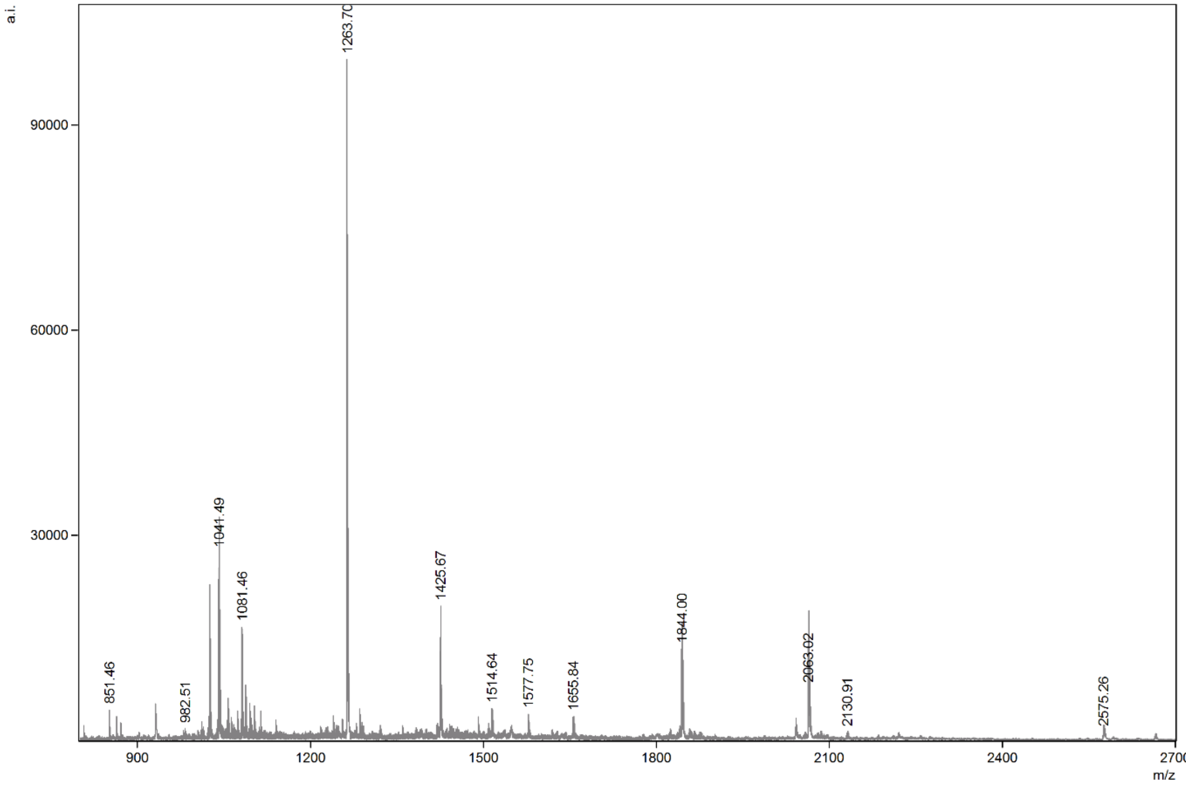

Figure A3: MS spectrum of Ava14 (Dorset)

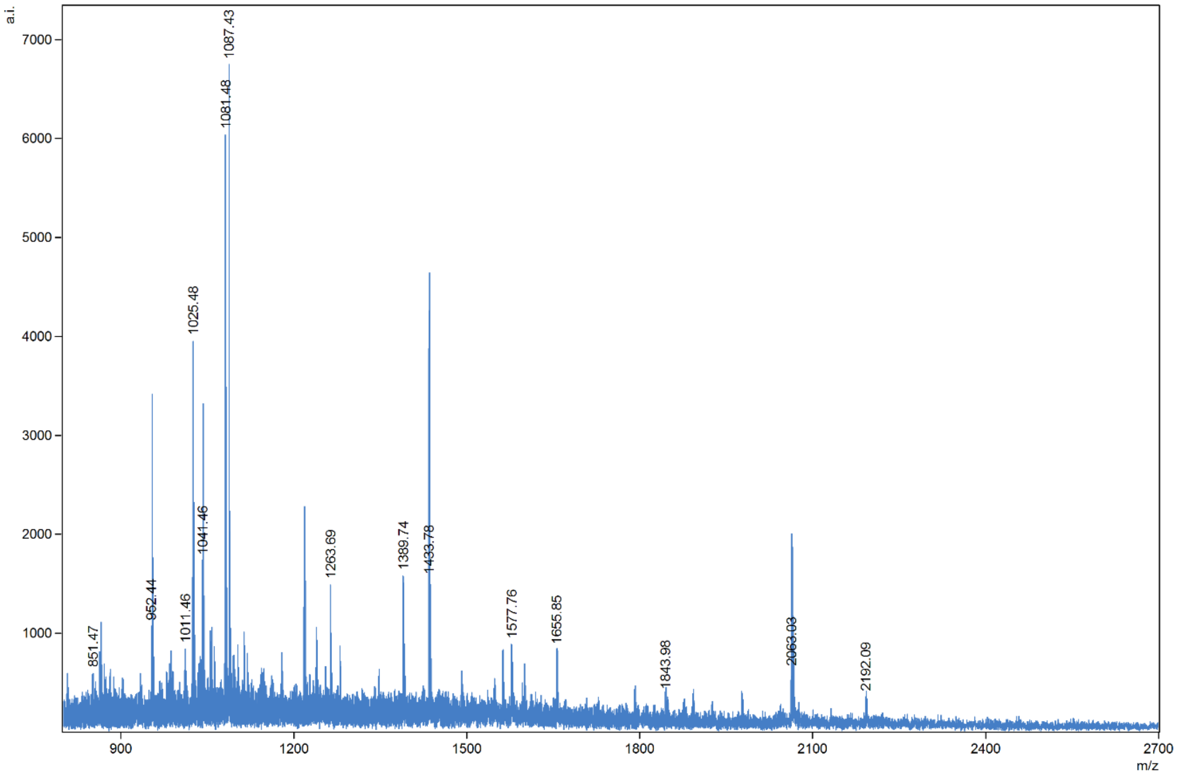

Figure A4: MS spectrum of Ava16 (Dorset)

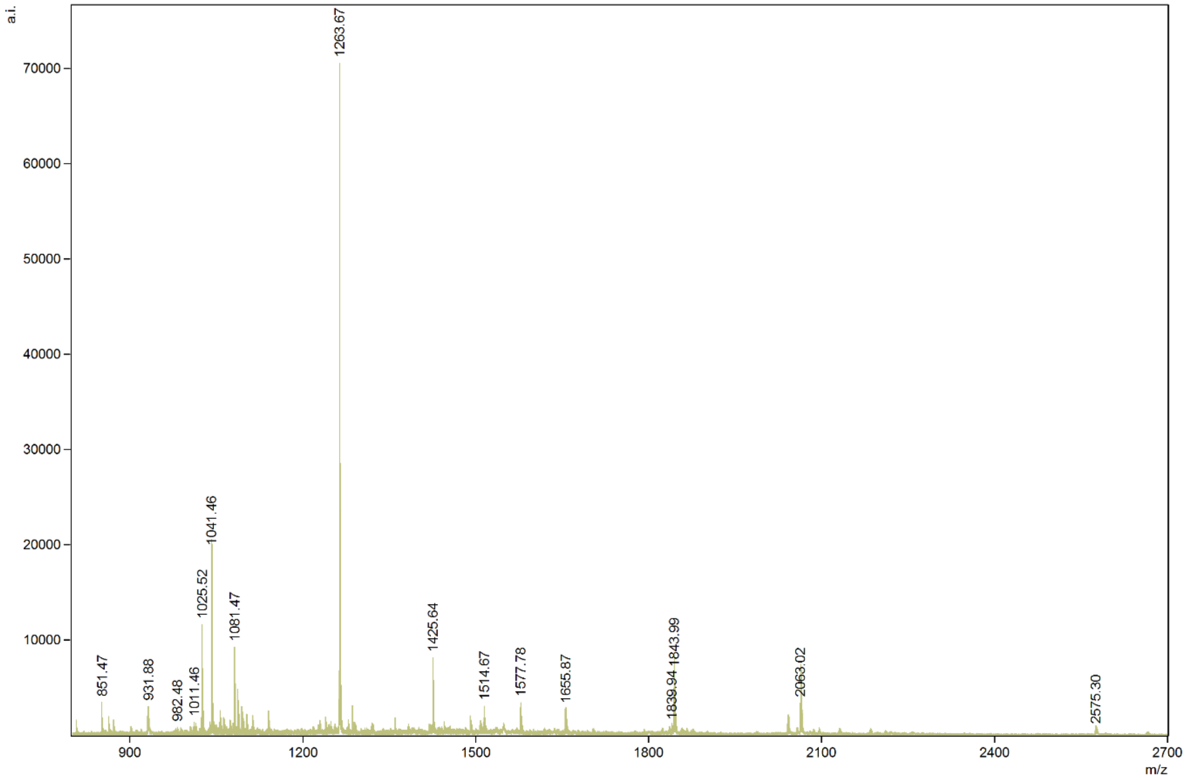

Figure A5: MS spectrum of Ava18 (Dorset)

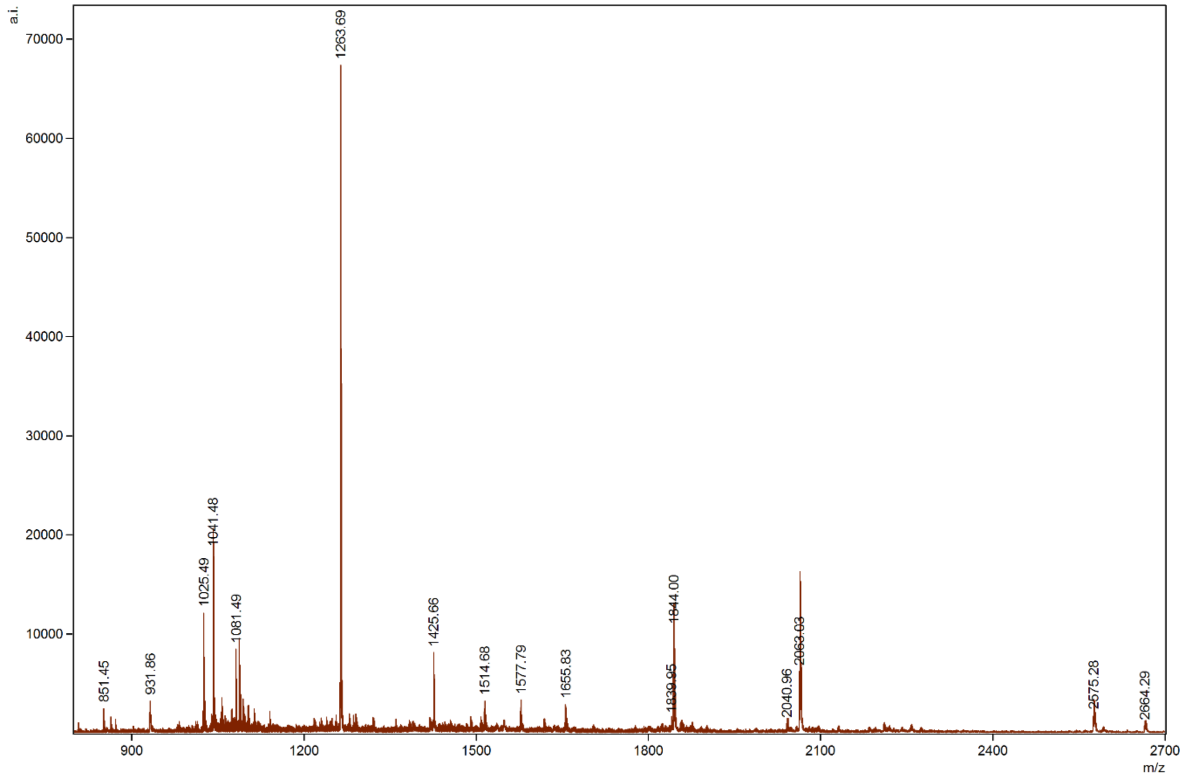

Figure A6: MS spectrum of Ava19 (Dorset)

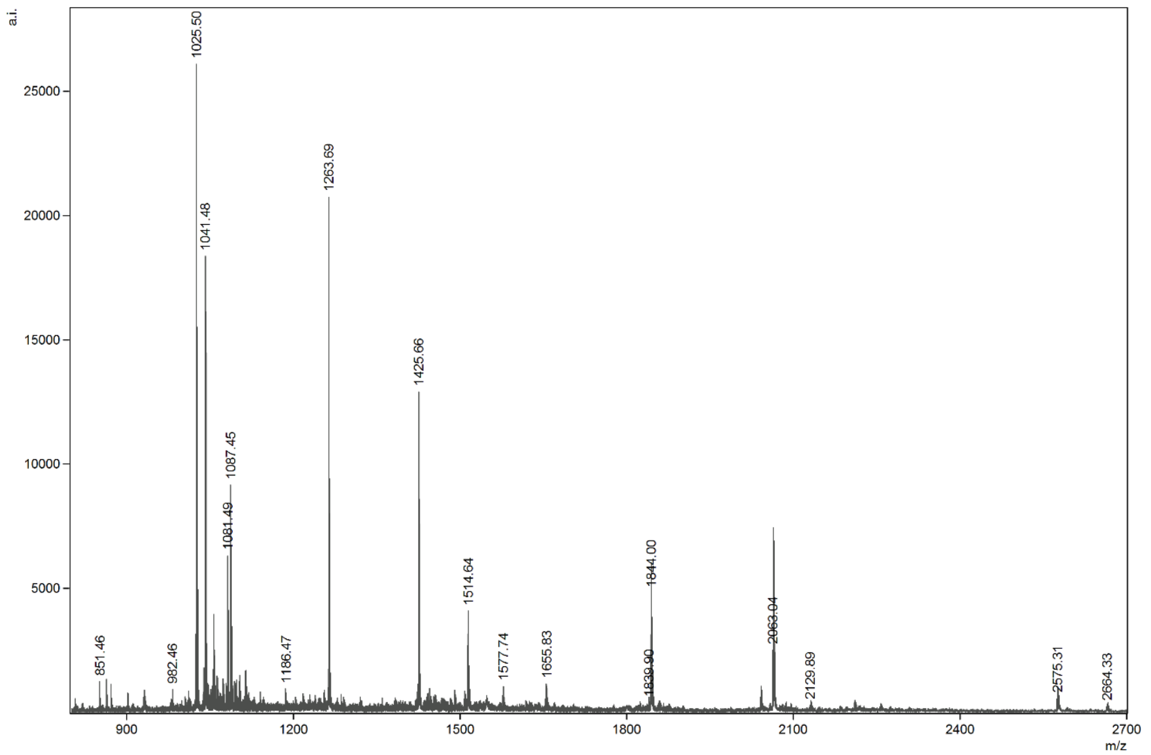

Figure A7: MS spectrum of Ava21 (Dorset)

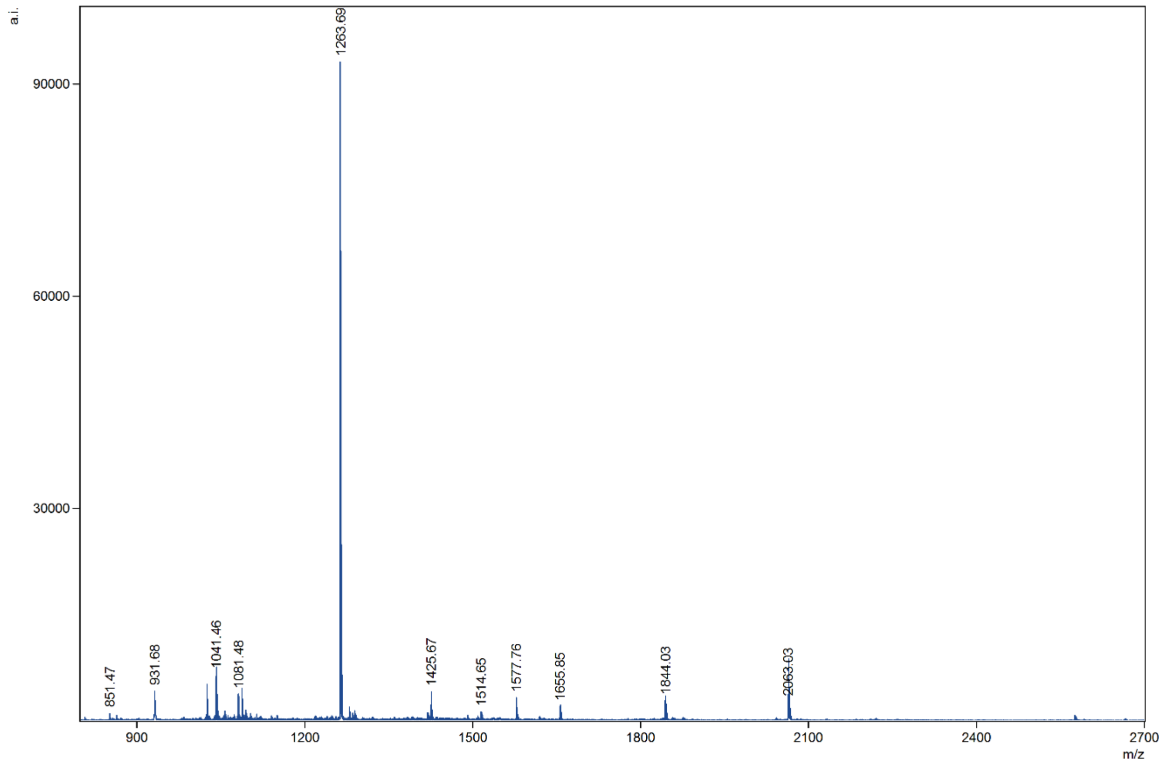

Figure A8: MS spectrum of Ava22 (Dorset)

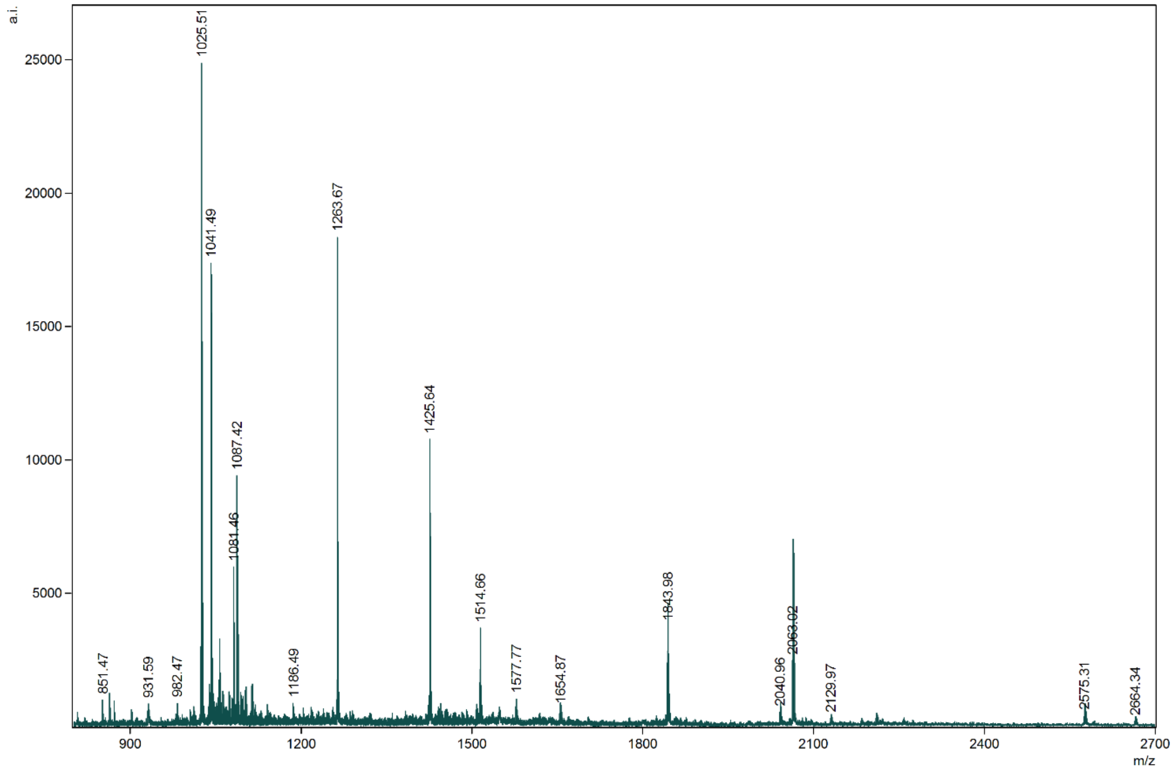

Figure A9: MS spectrum of Ava25 (Dorset)

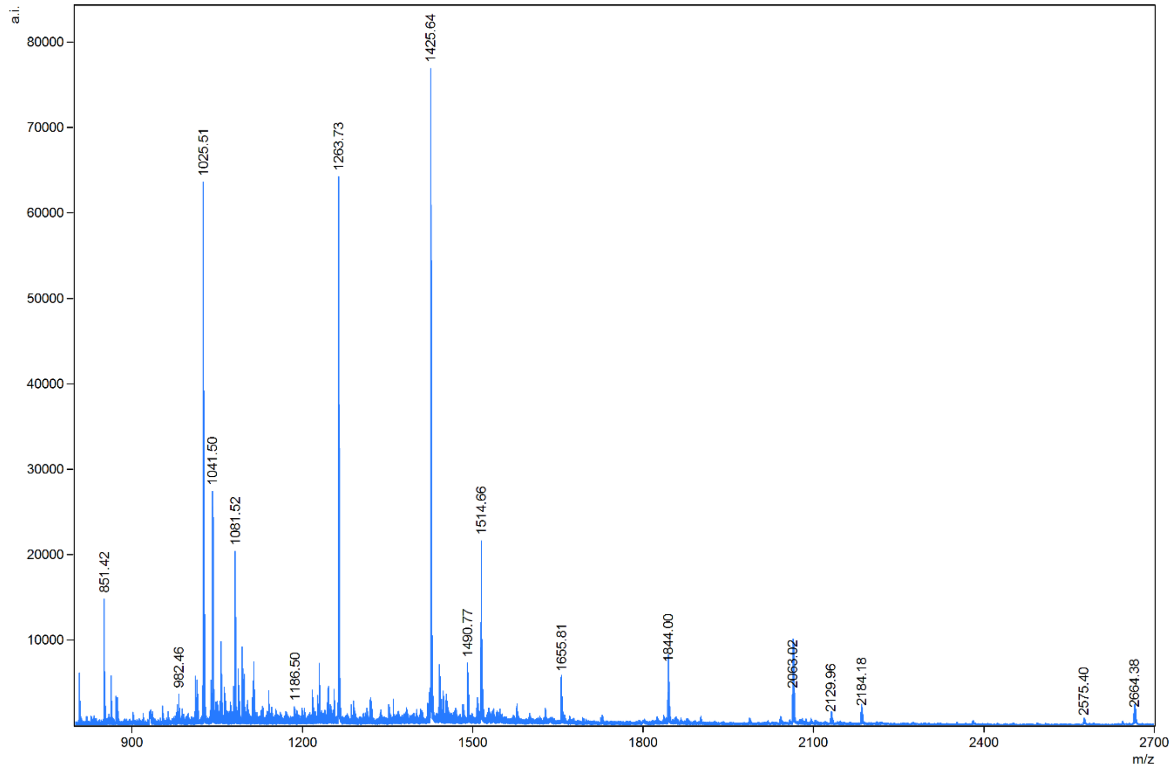

Figure A10: MS spectrum of Ava26_Strip (Dorset)

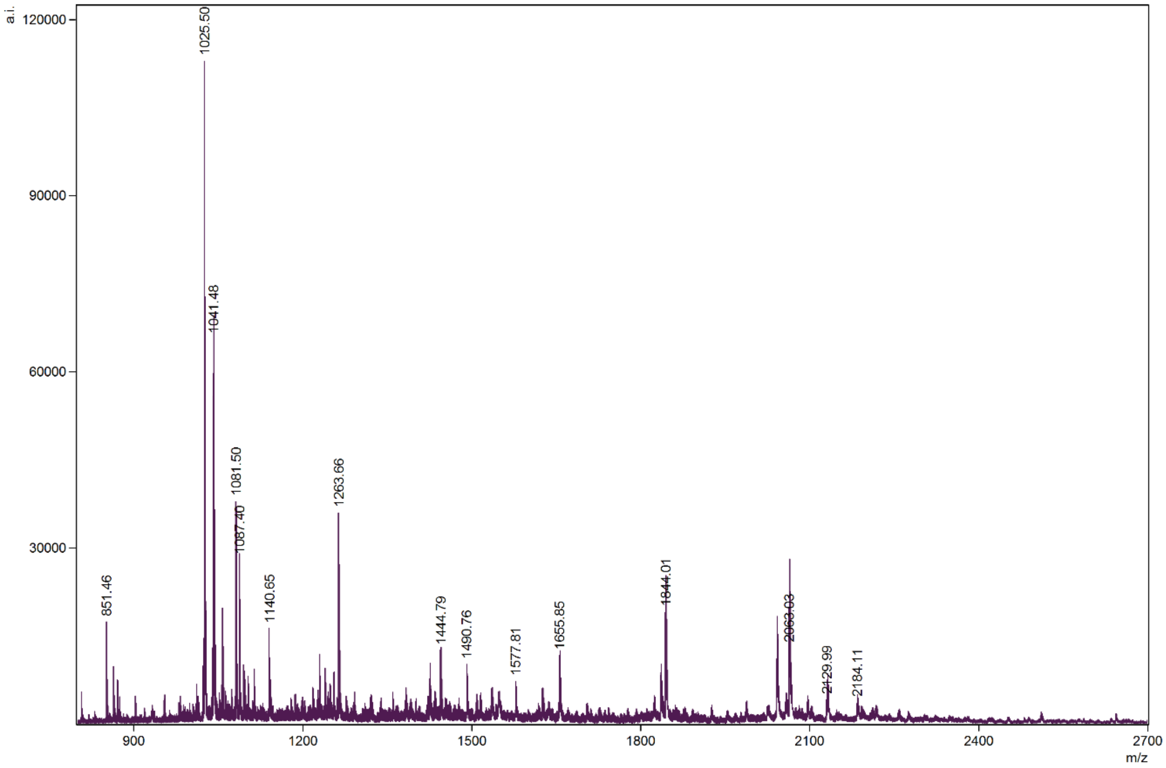

Figure A11: MS spectrum of Ava26_Bristle (Dorset)

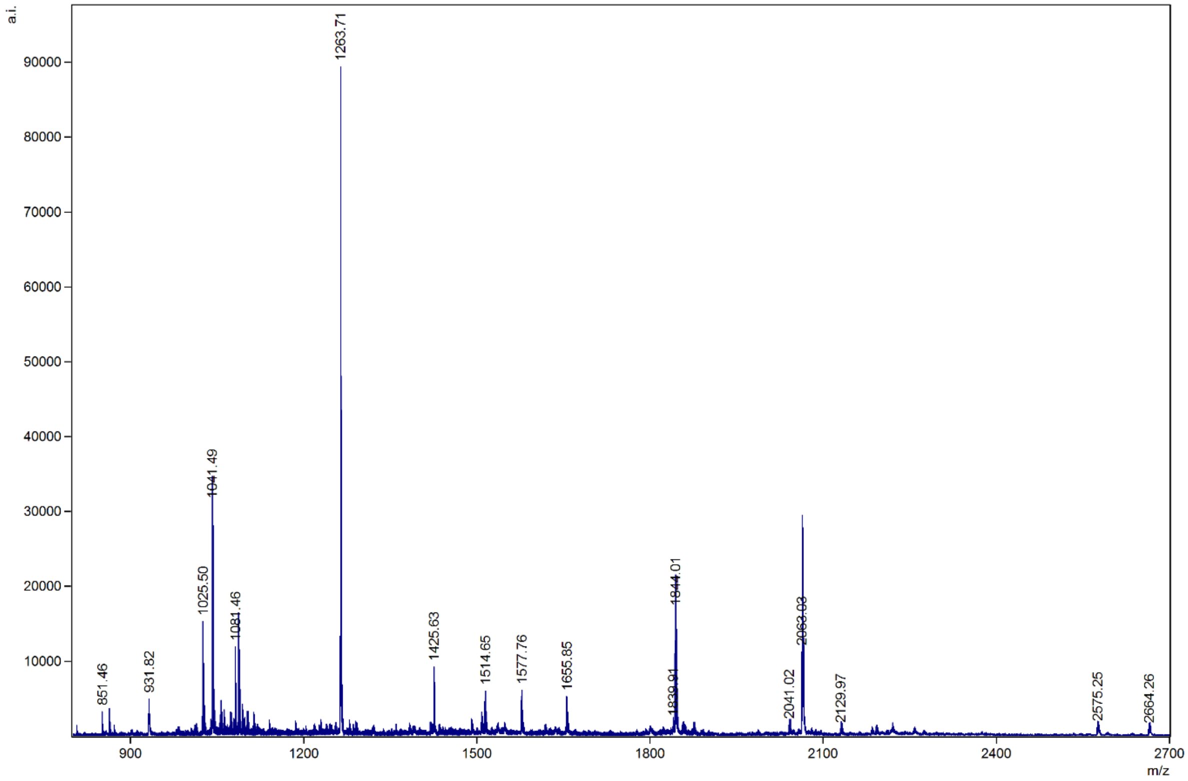

Figure A12: MS spectrum of Ava27 (Dorset)

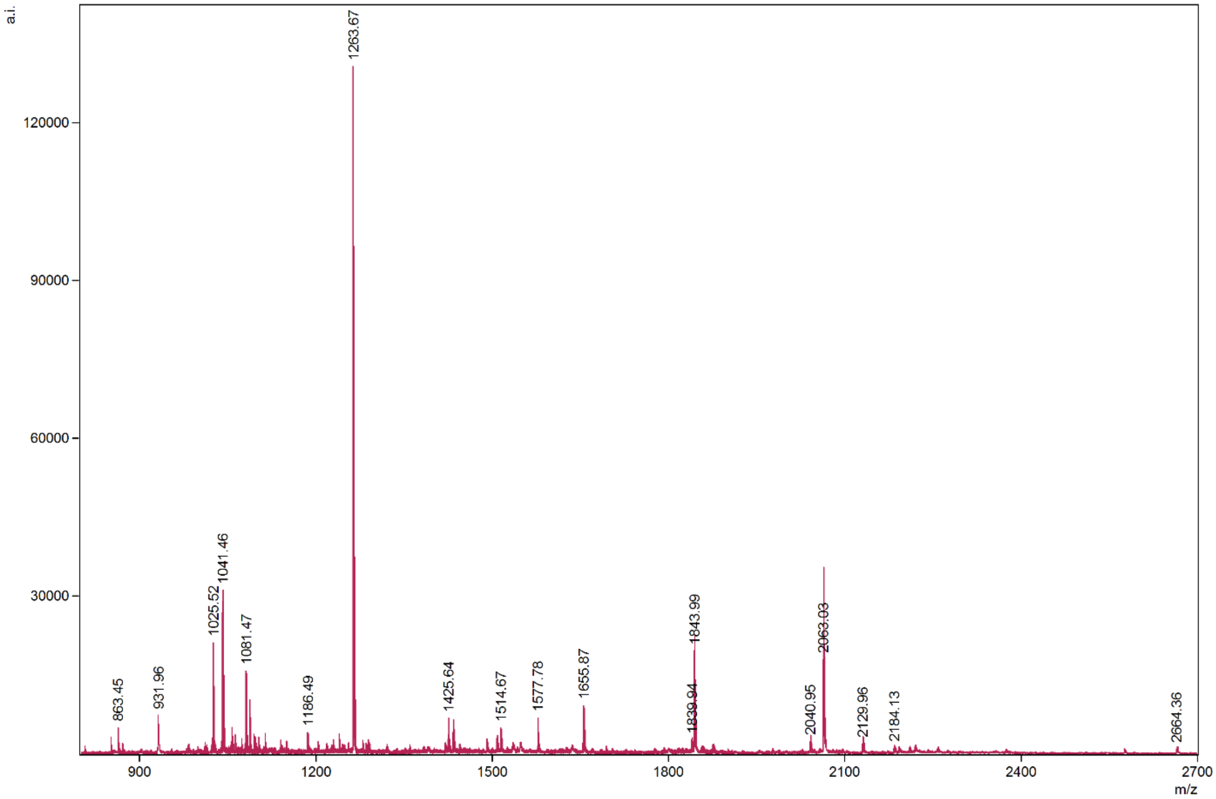

Figure A13: MS spectrum of Ava32 (Dorset)

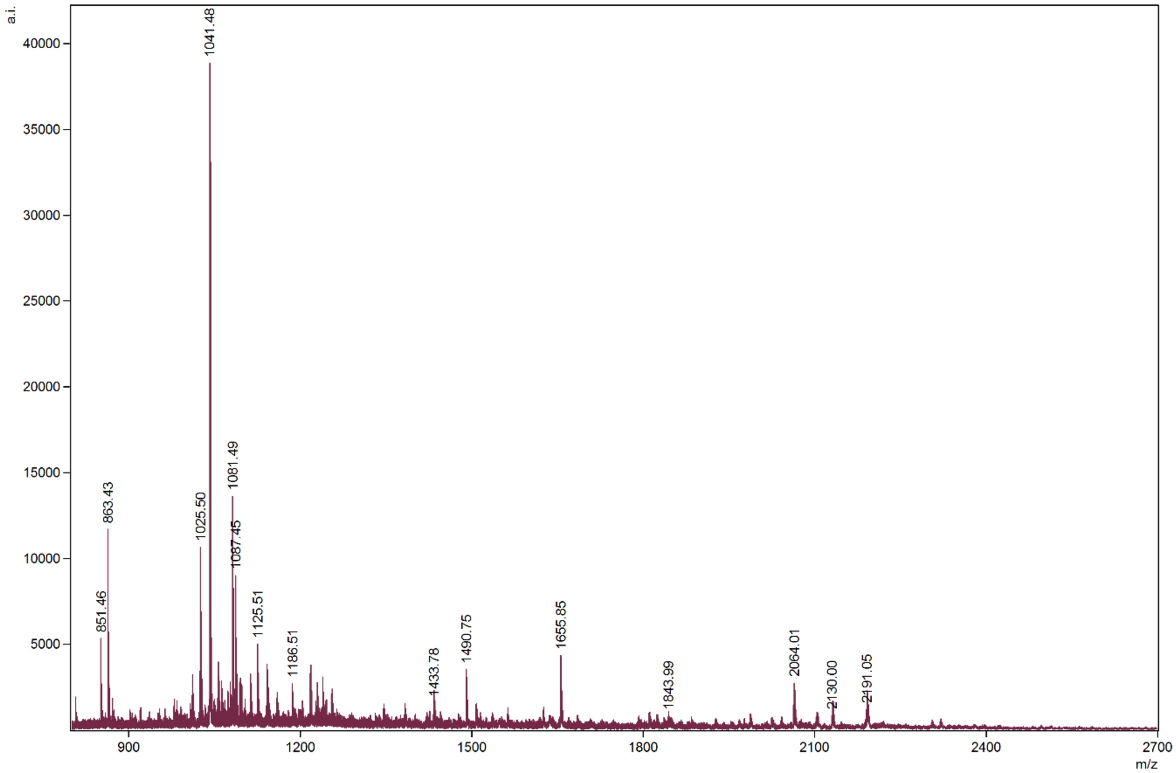

Figure A14: MS spectrum of Ava33 (Dorset)

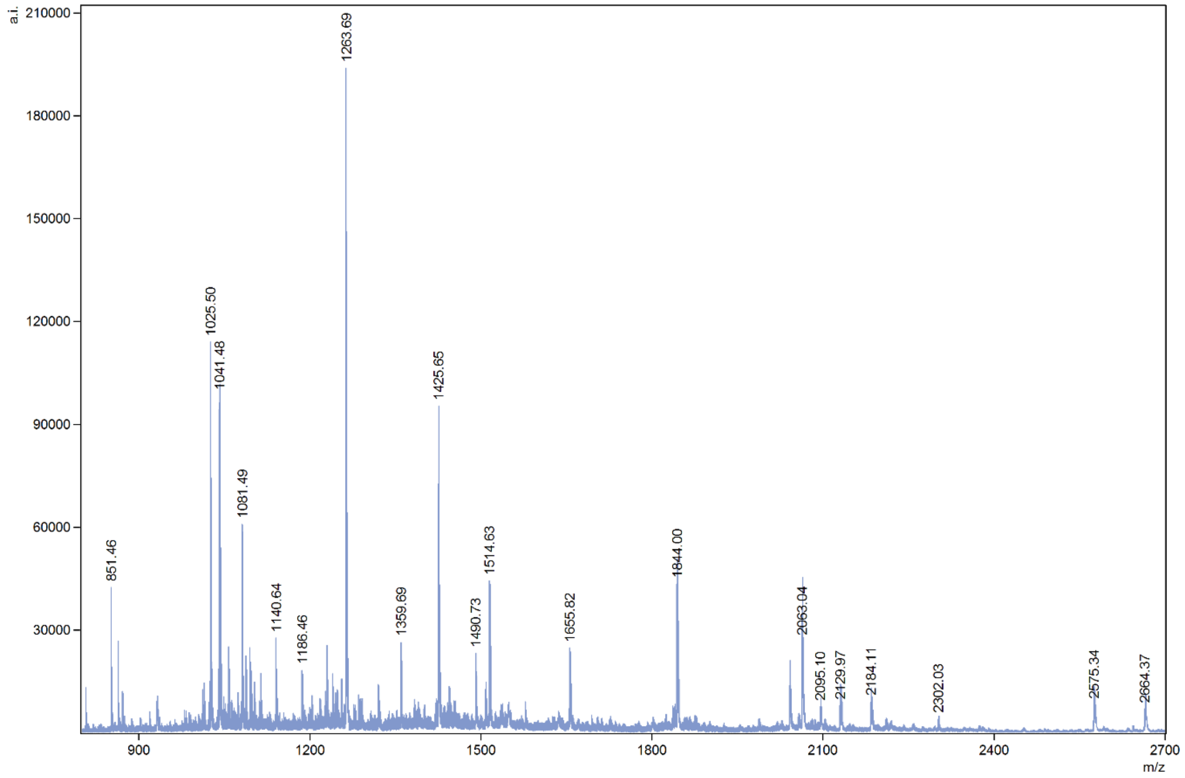

Figure A15: MS spectrum of Ava34 (Dorset)

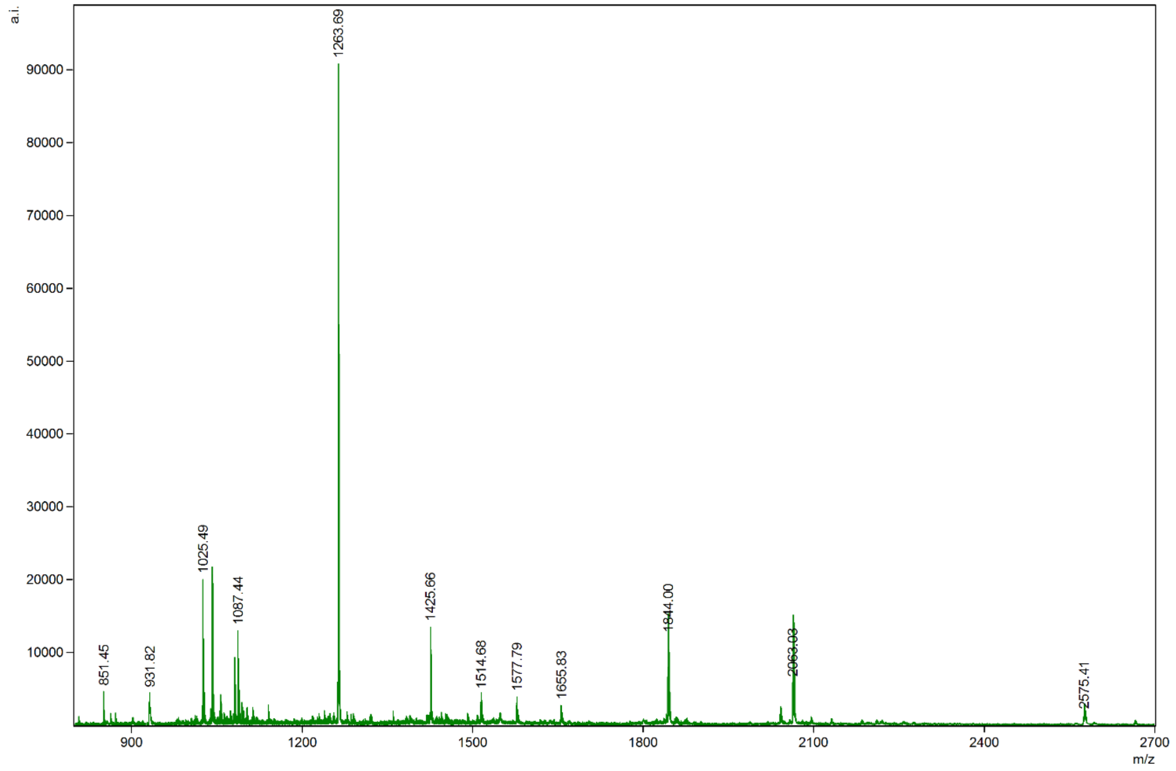

Figure A16: MS spectrum of Ava35 (Dorset)

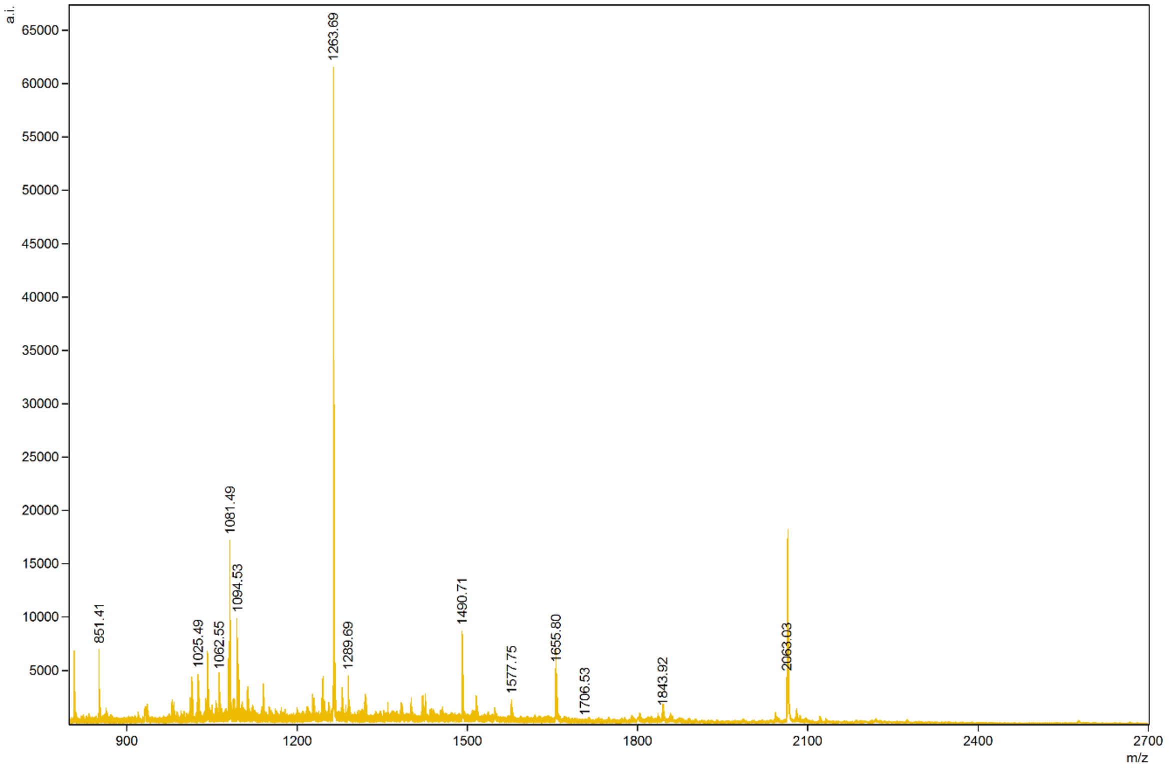

Figure A17: MS spectrum of HebH3_Strip (Inuit)

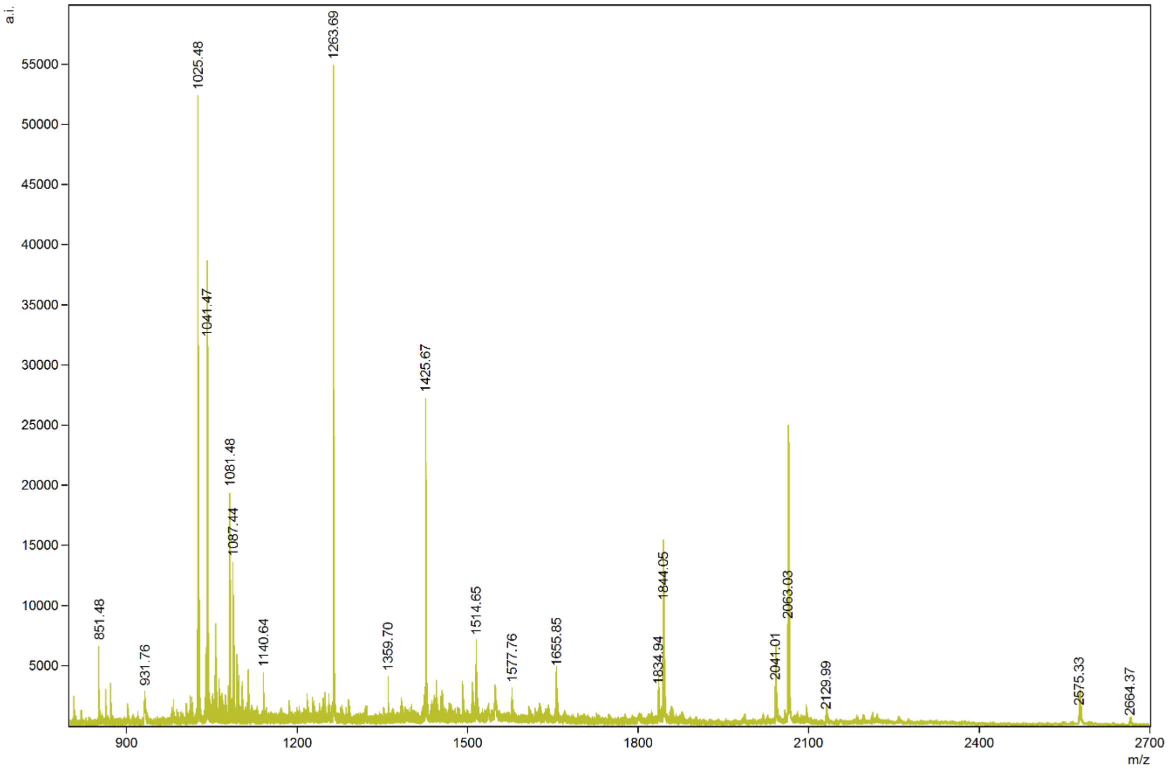

Figure A18: MS spectrum of Heb50_Bristle (Inuit)

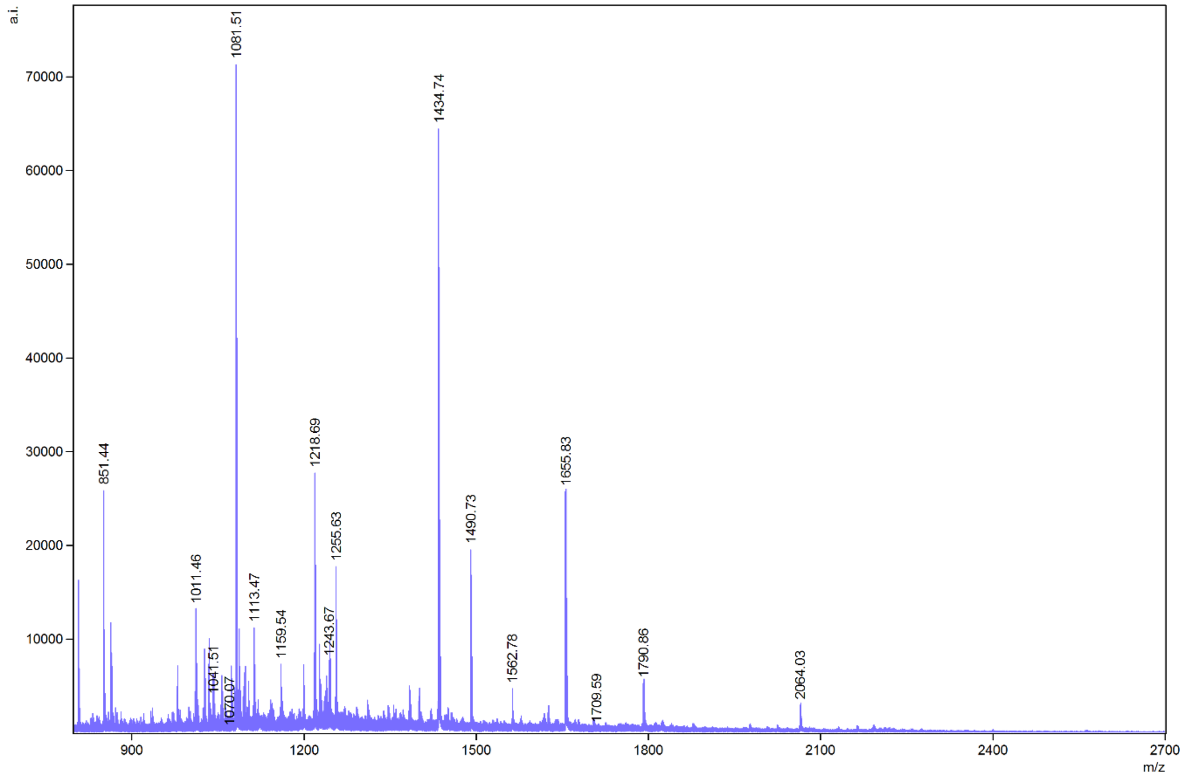

Figure A19: MS spectrum of Joh49_Strip (Inuit)

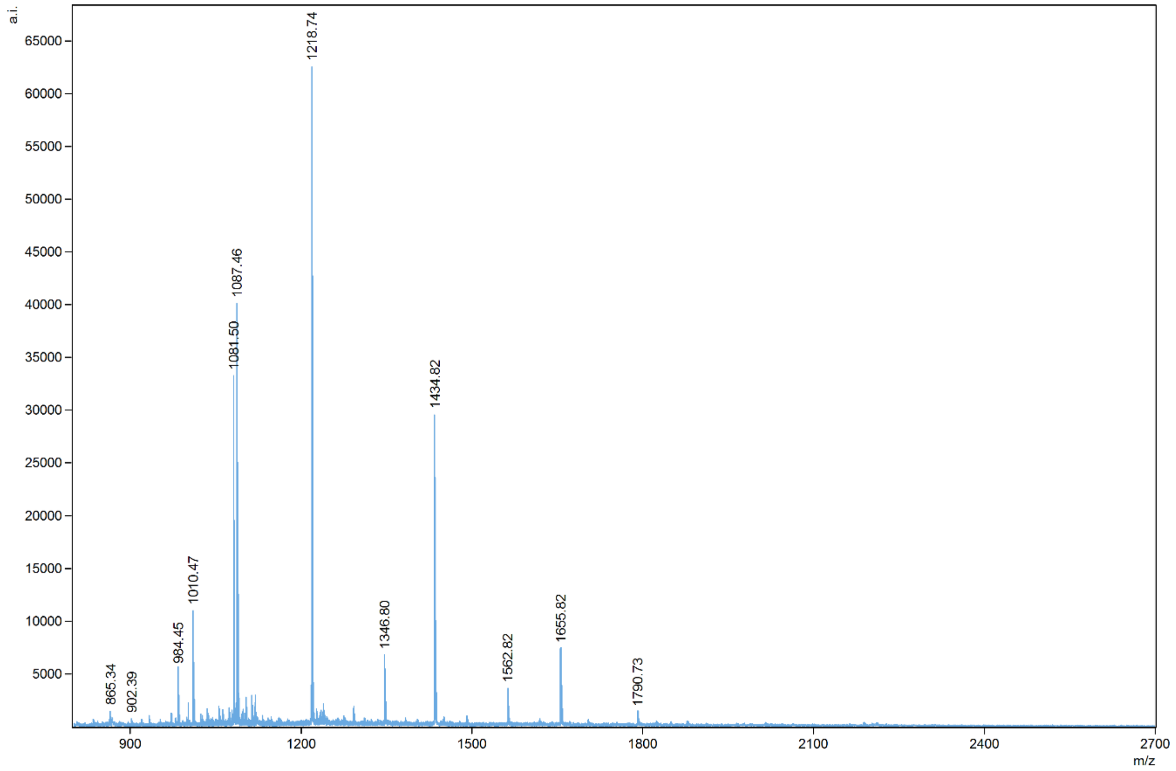

Figure A20: MS spectrum of Joh49_Bristle (Inuit)

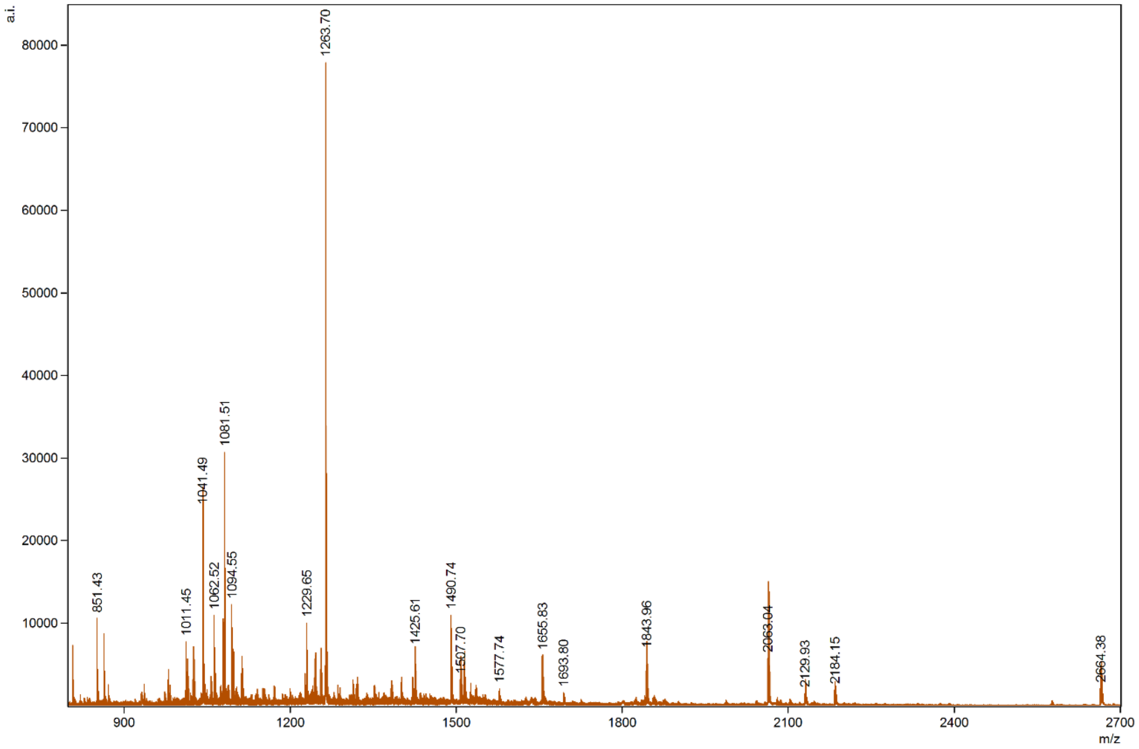

Figure A21: MS spectrum of Kom45_Strip (Dorset/Thule)

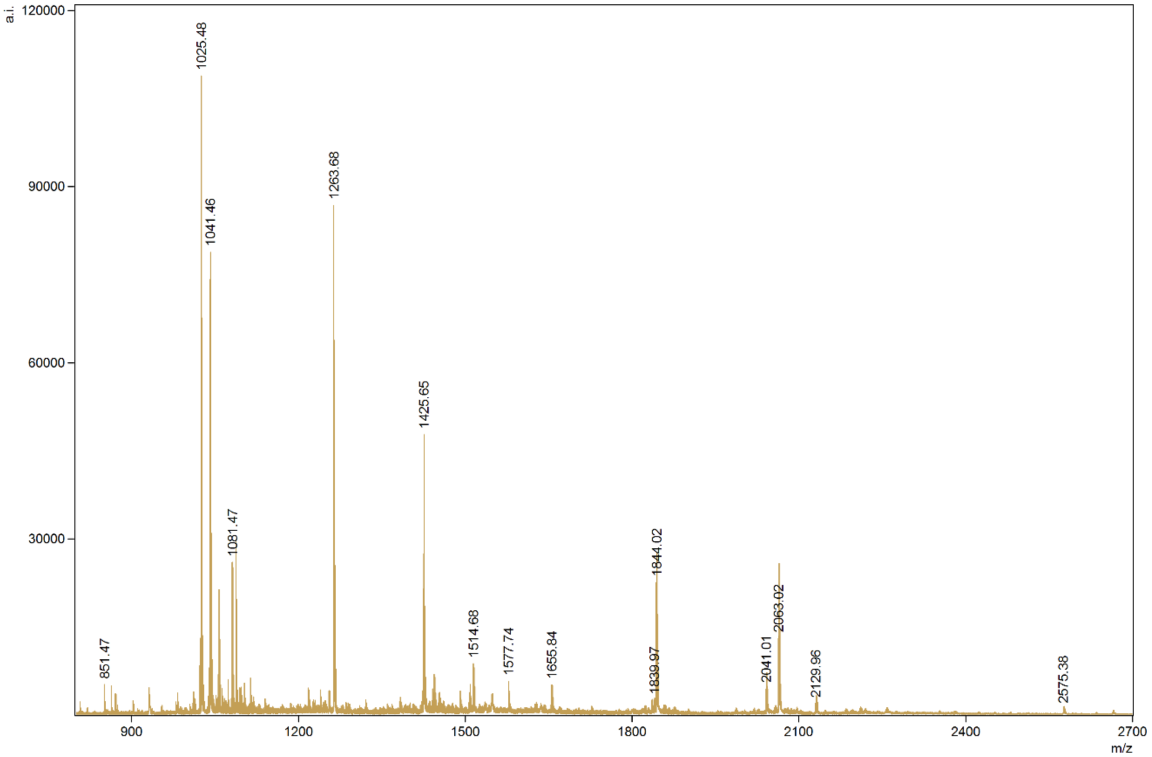

Figure A22: MS spectrum of Kom45_Bristle (Dorset/Thule)

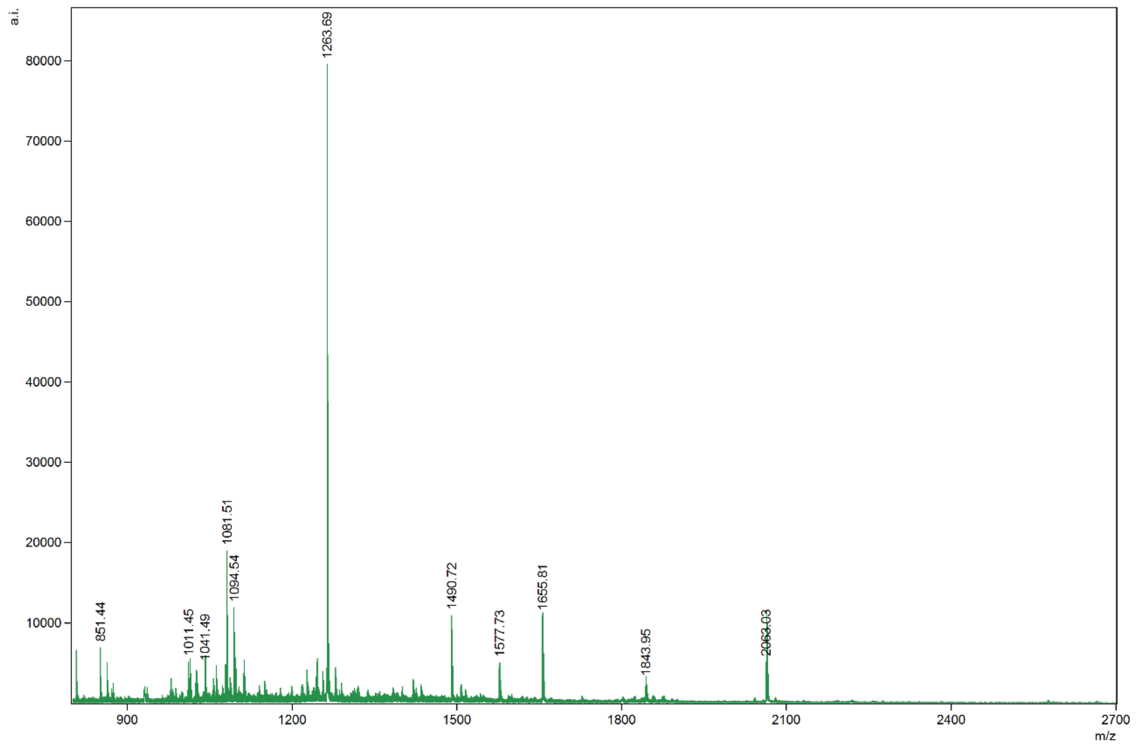

Figure A23: MS spectrum of Bush H1A (Thule)

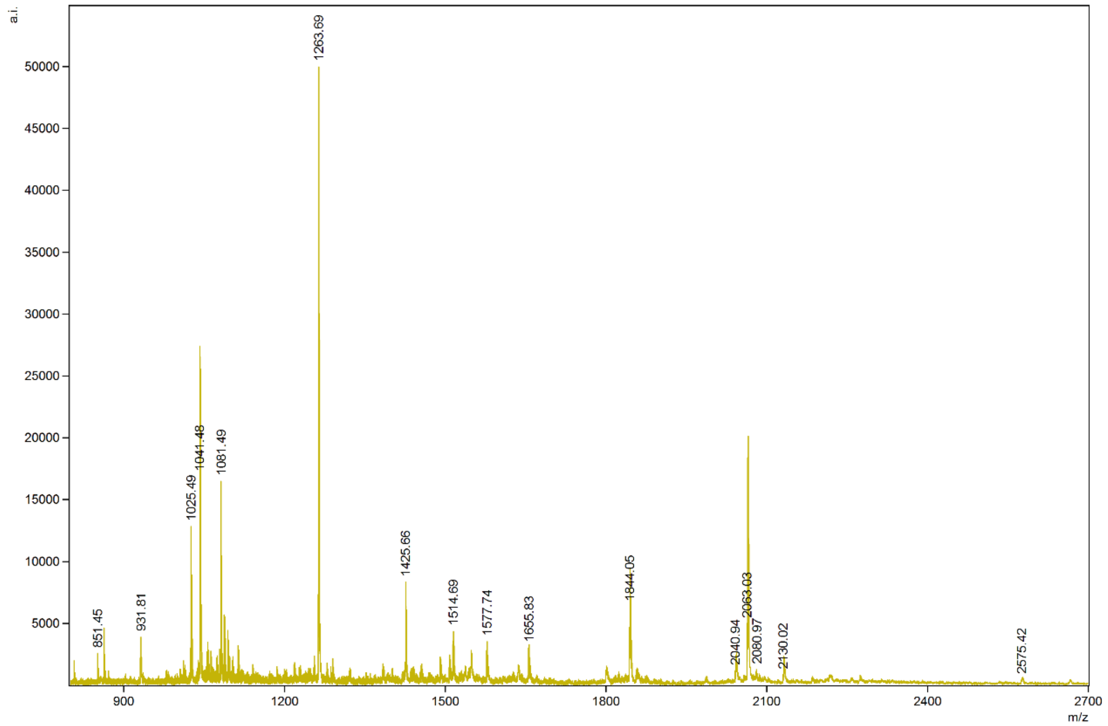

Figure A24: MS spectrum of Nach52 (Thule)

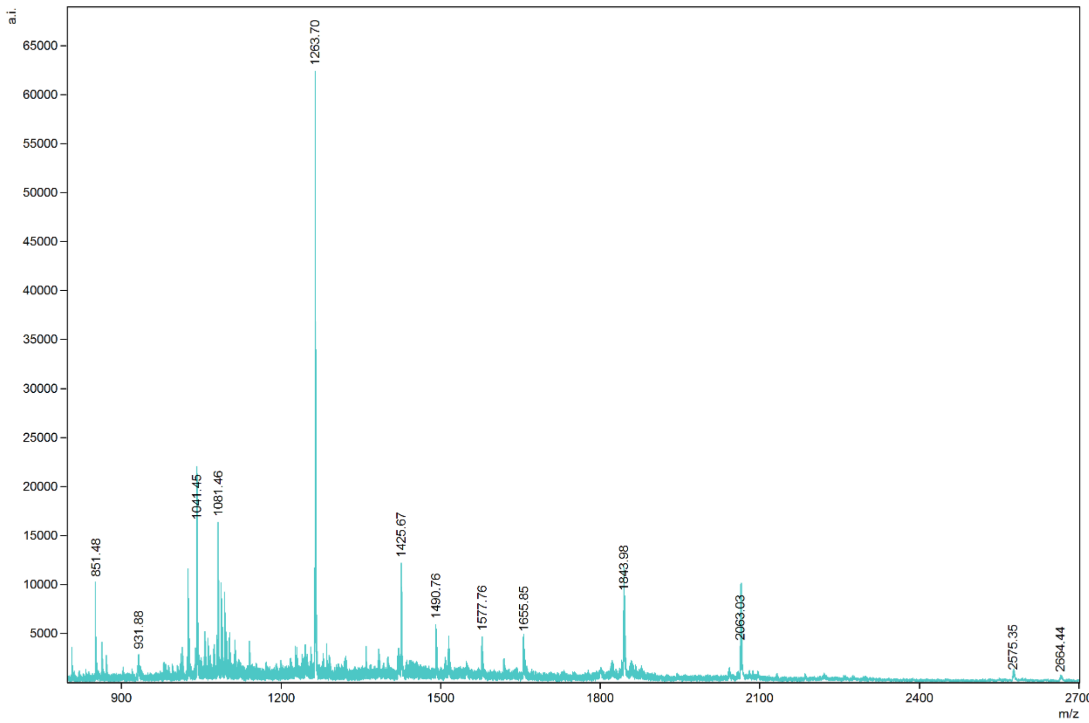

Figure A25: MS spectrum of Aku37 (Dorset)

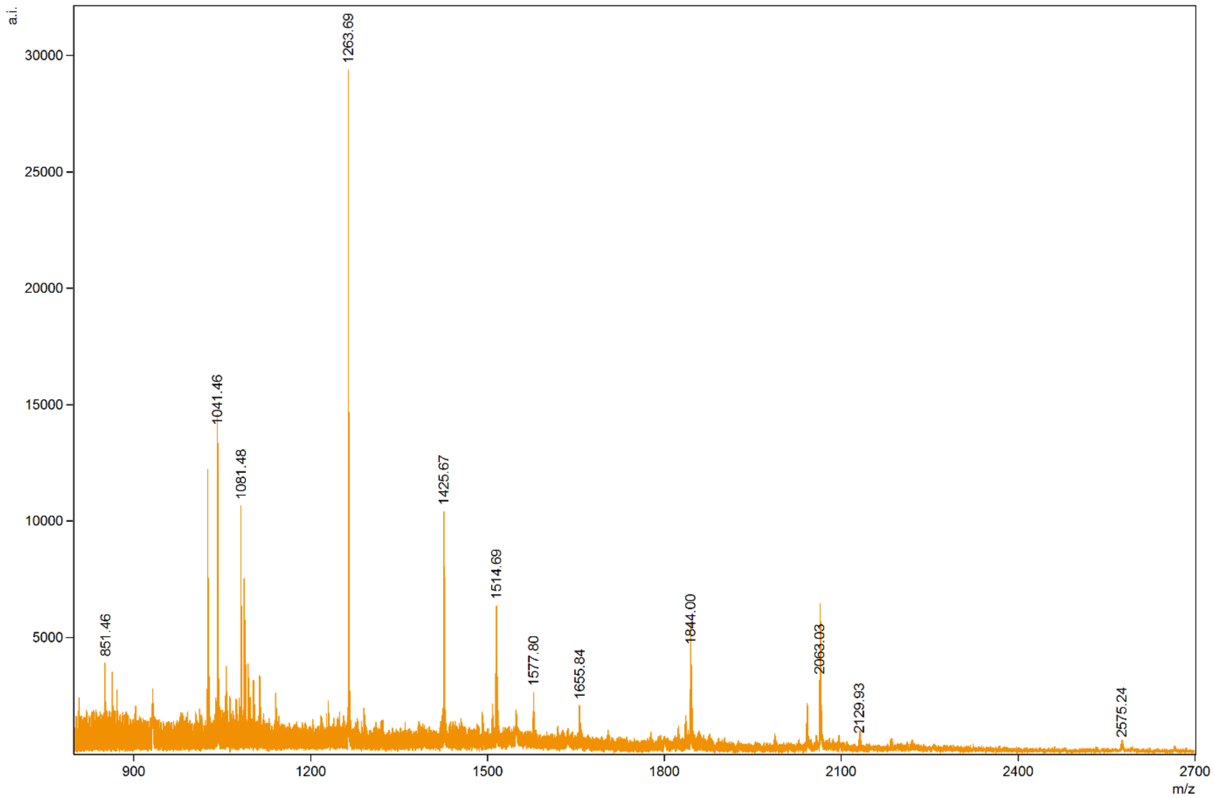

Figure A26: MS spectrum of Aku43 (Dorset)

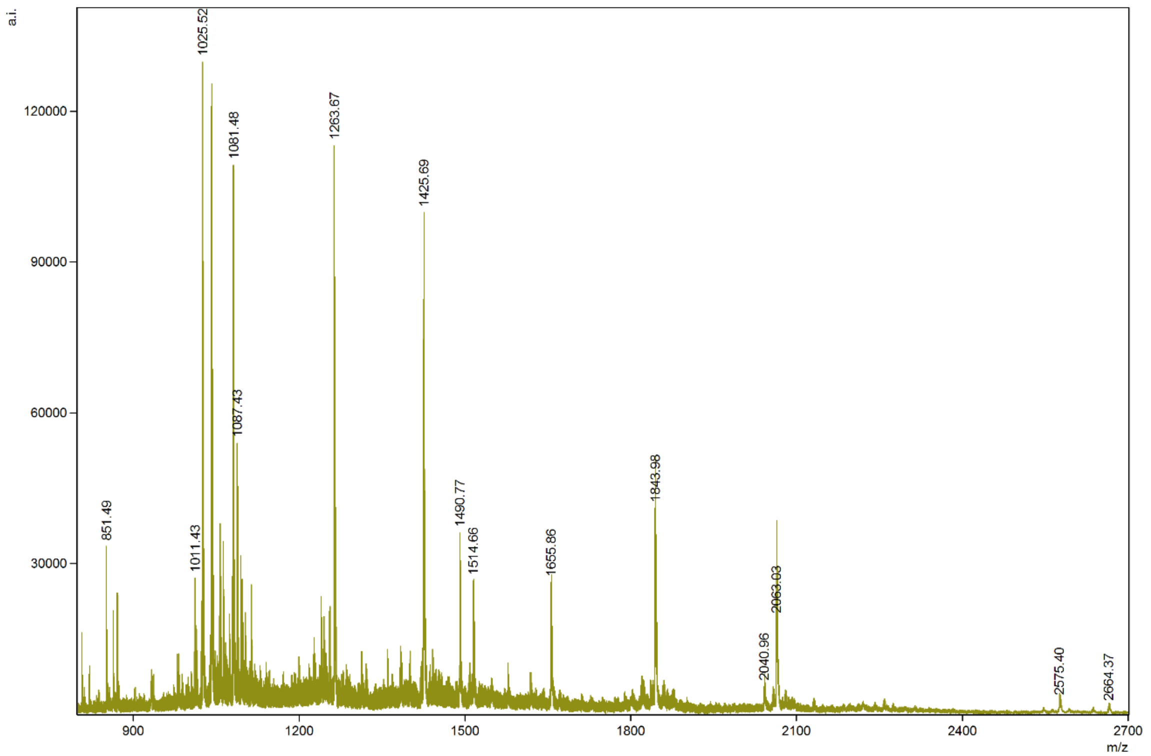

Figure A27: MS spectrum of Aku54 (Thule/Inuit)

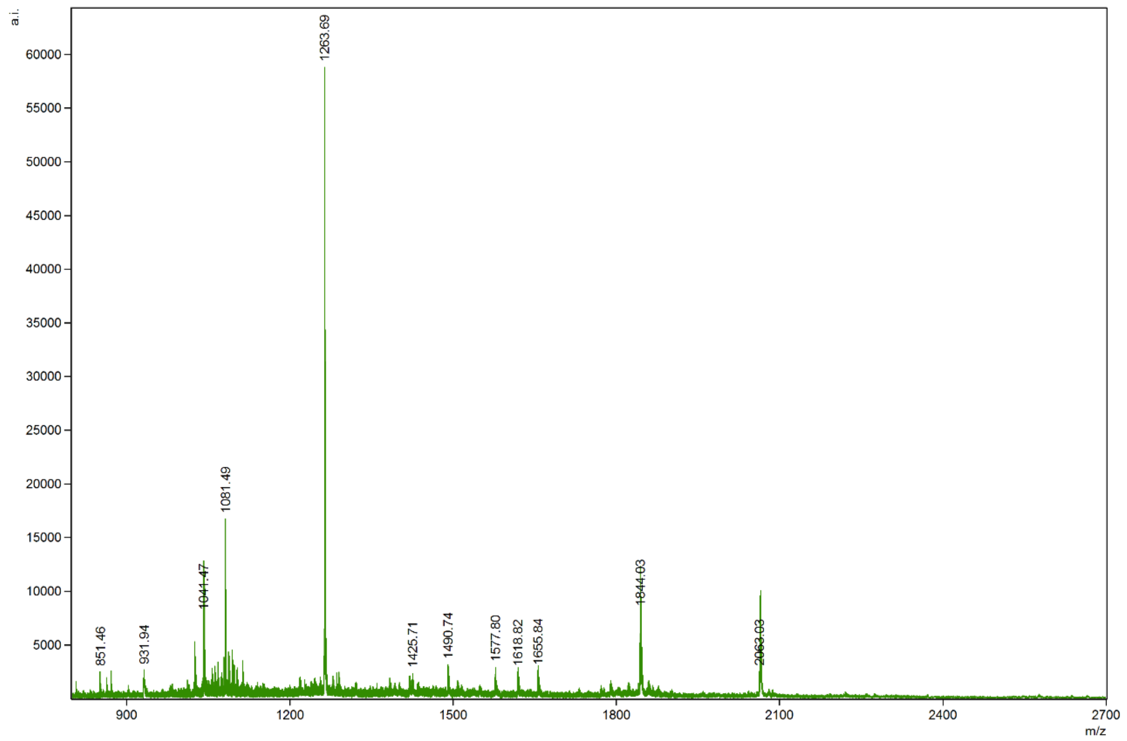

Figure A28: MS spectrum of Aku55 (Dorset)

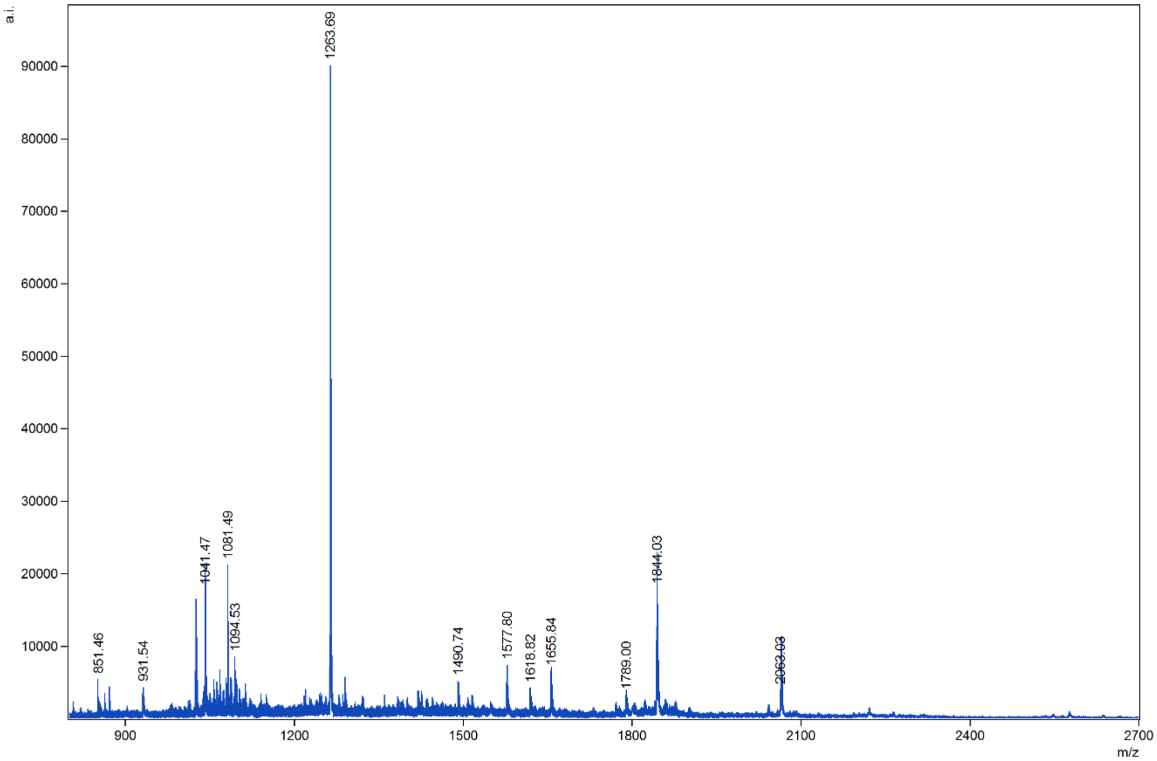

Figure A29: MS spectrum of Aku57 (Dorset)

S3-B. Considerations on deamidation in the archaeological baleen

Deamidation is a well-known modification that occurs in ancient proteins and is very useful in assessing the degree of degradation, and to a certain extent the antiquity of a sample. Recently, tools have been developed to monitor and quantify deamidation and the modification has been abundantly studied on both ancient collagen [12-17] and keratin [18, 19]. With their abundance of glutamine (Q) and asparagine (N), alpha-keratins are ideal proteins to study the deamidation process of these two residues in glutamic (E) and aspartic acid (D).

In a previous article, deamidation was quantified for key peptides in archaeological sheep wool [19]. Of the chosen peptides in that study, only one peptide is also present in baleen: peptide SDLEANSEALIQEIDFLR at *m/z* 2063.03. Two other peptides have known homologous sequences: LNVEVDAAPTEDLNR at *m/z* 1655.82 (LNVEVDAAPTVDLNR at *m/z* 1625.84 in sheep) and TVNALEIELQAQHSMR at *m/z* 1839.94 and TVHALEVELQAQHNLR at *m/z* 1857.99 (TVNALEVELQAQHNLR at *m/z* 1834.97 in sheep). In the bowhead whale however, the peptide at *m/z* 1857.99 is substituted by one at *m/z* 1843.95 (unknown sequence). Finally the bowhead whale has a peak at *m/z* 2664.37, YTSQLAQIQCLISNVEAQLSEIR, homologous to the sheep peptide YSCQLNQVQSLIVSVESQLAEIR (also *m/z* 2664.37).

In the study of sheep wool based on MALDI-TOF profiles of archaeological wool, it was shown that *m/z* 2063 had the highest degree of deamidation, followed by *m/z* 1835. The peaks at *m/z* 1625 and 2664 indicated limited deamidation for these peptides.

To evaluate the extent of deamidation in our archaeological baleen, we looked at four peptides that were present in high intensity: LNVEVDAAPTEDLNR at *m/z* 1655.82, SDLEANSEALIQEIDFLR at *m/z* 2063.03, WQFYQNR at *m/z* 1041.49 and the peptide at *m/z* 1843.95 (assuming its sequence is

homologous to the other sequences listed above). The peak at *m/z* 2664.37 was missing in too many archaeological samples to be representative. The ratios between the first two isotopic peaks was calculated and compared to the theoretical ratio obtained by using the MS-Isotope program in Prospector (http://prospector.ucsf.edu):

Example on peptide WQFYQNR at *m/z* 1041.49:

Elemental Composition: **C49 H65 N14 O12**

Profile Type: **Gaussian**
Resolution: **10000**

Isotope Number	*m/z*	Percent Total	Percent Maximum
0	1041.49009	52.97	100.00
1	1042.49297	32.53	61.42
2	1043.49566	11.12	21.00
3	1044.49826	2.74	5.17
4	1045.50081	0.54	1.02
5	1046.50332	0.09	0.17
6	1047.50579	0.01	0.02
7	1048.50841	0.00	0.00
8	1049.51089	0.00	0.00

The theoretical abundance ratio (Fig S3-1a) between the two peaks is calculated as: IN0/IN1=52.97/32.53=1.628 where IN0 represents the monoisotopic peak and IN1 the next peak. When an asparagine or glutamine residue is deamidaded, each deamidaded residue adds +0.984 Da resulting in a mass shift to 1042.474 and so on, thus changing the shape of the isotopic distribution (Fig 3-1b). By measuring the ratio between the first two peaks, we can evaluate whether the peptide is deamidated. This ratio is normalized to 1 by dividing it by the theoretical value of the non-deamidated peptide. A IN0/IN1 value of zero would indicate that at least one residue of the peptide is fully deamidated.

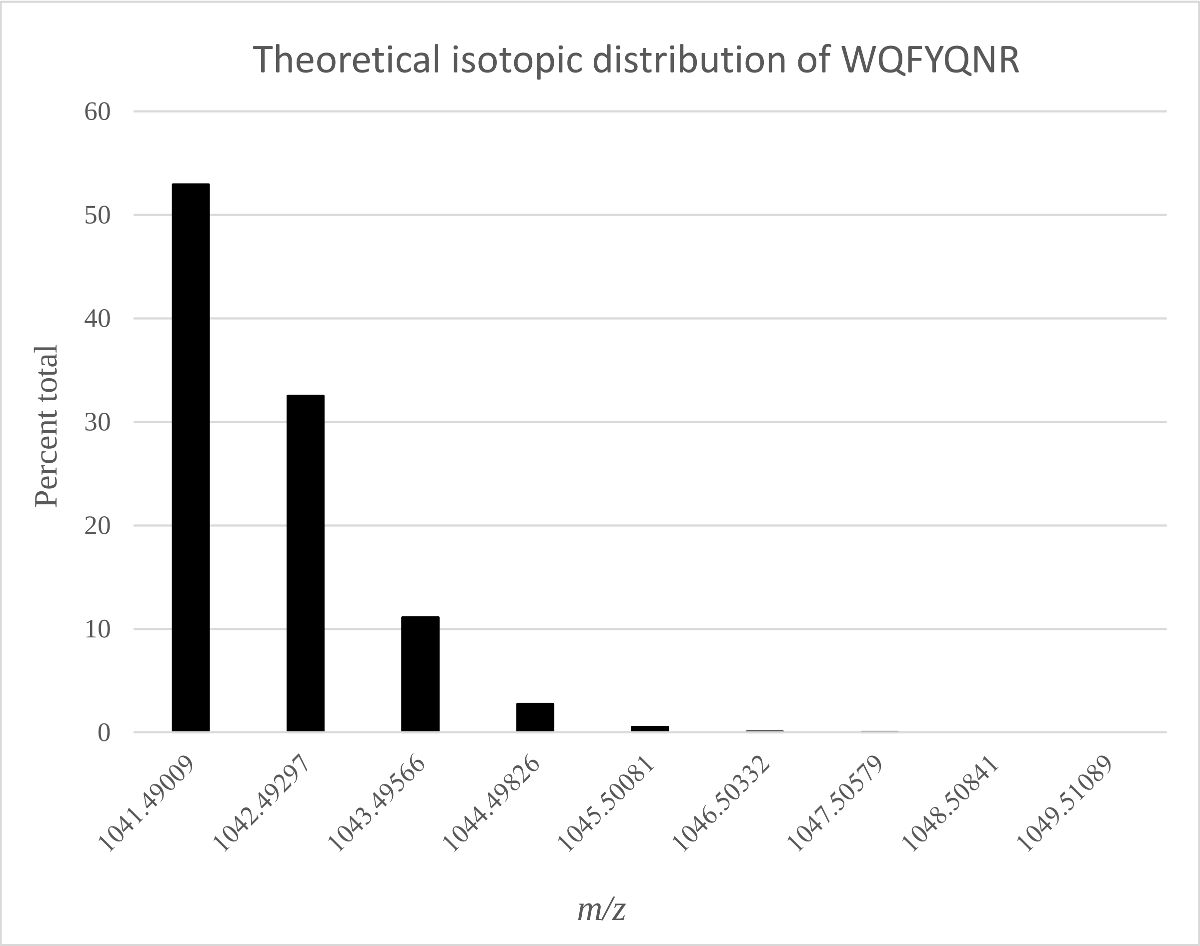

Figure B1: theoretical isotopic distribution of WQFYQNR (non-deamidated peptide)

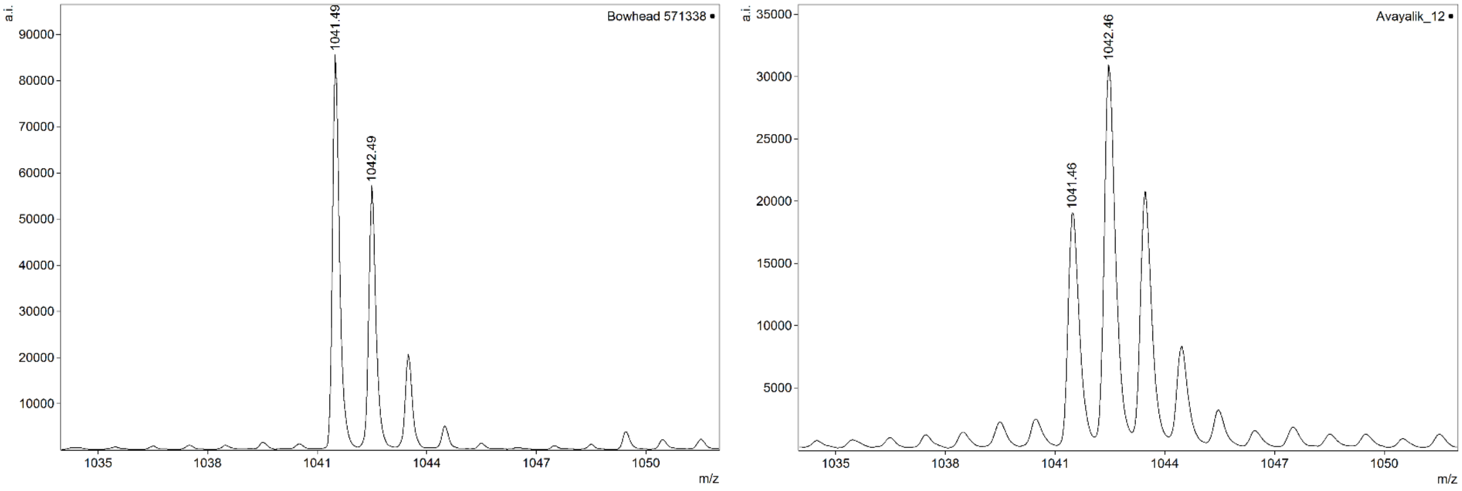

Figure B2: peptide WQFYQNR in the reference bowhead whale sample 571338 and in an archaeological sample Avayalik_12, showing the changes in the shape of the peak

To evaluate the extent of deamidation, we calculated the ratio in the archaeological samples on the intensity of the peaks (using relative intensity). The ratios obtained are considered to be only an approximation and not a precise quantification of deamidation for each peak (which would require more complex mathematical calculations, especially as the alpha-keratin peptides often contain multiple glutamine and asparagine as potential deamidation sites). Table S3-2 indicates the average (with standard deviation) of the values obtained for four sites.

Table B: Average values of normalized IN0/IN1 ratio for selected peptides and archaeological sites. * Ratios normalized to 1: IN0/IN1=1 indicates a non-deamidated peptide and IN0/IN1=0 a peptide with one site fully deamidated. Only sites with at least two samples are included here. The Johannes Point is not included either because of the quality of the spectra and missing peaks.

	n	IN0/IN1* *m/z* 1041	IN0/IN1* *m/z* 1655	IN0/IN1* *m/z* 1844	IN0/IN1* *m/z* 2063
**Reference Bowhead 571338**	**1**	**0.92**	**0.94**	**0.96**	**0.89**
Komaktorvik	2	0.62 (0.10)	088 (0.03)	0.81 (0.01)	0.49 (0.10)
Avayalik	16	0.55 (0.09)	0.87 (0.14)	0.78 (0.06)	0.44 (0.10)
Akulialuk	5	0.51 (0.11)	0.82 (0.06)	0.75 (0.06)	0.43 (0.11)
Hebron	2	0.42 (0.12)	0.72 (0.10)	0.63 (0.06)	0.32 (0.07)

From these values, the first observation is that for each peptide, we see a consistent pattern in deamidation over each site, with Komaktorvik showing the least deamidation, followed by Avayalik, Akulialik and finally Hebron as the most deamidated. This last site Hebron has noticeable higher deamidation levels compared to the other three sites and is also a neighbouring site to Johannes Point where the most degraded samples were found. The second observation is that the peptide with the highest level of deamidation is *m/z* 2063. As in wool [19], deamidation is high in the archaeological samples for this peptide, with particularly low IN0/IN1 at Hebron. Likewise, *m/z* 1844 shows high levels of deamidation

consistent with previous observations, while values for *m/z* 1655 indicate again a slower rate of deamidation for this peptide.

<u>Conclusions on deamidation</u>: This exercise is consistent with previous observations on archaeological wool [19] and indicates that deamidation occurred in the baleen samples with various degrees. In particular, it indicates faster deamidation at the Hebron site, close to Johannes Point. The trend observed here is independent of the time period of the samples and most likely represents preservation at the different sites. However, of the five Akulialuk samples, four are Dorset and one is Thule/Inuit (Aku54); the latter sample has generally lower levels of deamidation than the former (see tables below).

While the ratio calculated here is not intended to give accurate values of deamidation in our samples, it is useful to show the occurrence of deamidation and some patterns of degradation at the different sites. For accurate measure of deamidation, several studies [13, 19] have tackled the subject and offered better mathematical models; this was beyond the scope of this study.

<u>Complete deamidation tables:</u>

	N0/N1 *m/z* 1041	N0/N1 *m/z* 1655	N0/N1 *m/z* 1844	N0/N1 *m/z* 2063
**Theoretical**	**1.63**	**1.14**	**1.00***	**0.90**
Reference Bowhead 571338	1.50	1.08	0.96	0.80
Normalised Bowhead	0.92	0.94	0.96	0.89

* N0/N1=0.98 for *m/z* 1858 and N0/N1=1.01 for *m/z* 1840: an arbitrary value of 1.00 was therefore chosen for the *m/z* 1844 peptide

m/z **1041**	**Ava12**	**Ava13**	**Ava14**	**Ava16**	**Ava18**	**Ava19**	**Ava21**	**Ava22**	**Ava25**	**Ava26_B**	**Ava26_S**	**Ava27**	**Ava32**	**Ava33**
**N0/N1***	0.38	0.56	0.51	0.32	0.58	0.54	0.63	0.53	0.61	0.58	0.66	0.55	0.62	0.64
**Ava34**	**Ava35**	**HebH3_S**	**Heb50_B**	**Joh49_S**	**Joh49_B**	**Kom45_S**	**Kom45_B**	**Bush Is**	**Nachvak**	**Aku37**	**Aku43**	**Aku54**	**Aku55**	**Aku57**
0.59	0.53	0.34	0.50	0.34	NA	0.55	0.70	0.47	0.38	0.44	0.59	0.63	0.37	0.50

* Normalized values

m/z **1655**	**Ava12**	**Ava13**	**Ava14**	**Ava16**	**Ava18**	**Ava19**	**Ava21**	**Ava22**	**Ava25**	**Ava26_B**	**Ava26_S**	**Ava27**	**Ava32**	**Ava33**
**N0/N1***	0.44	0.92	0.86	0.85	0.82	1.00	1.04	0.78	1.02	0.82	0.84	0.92	0.93	0.93
**Ava34**	**Ava35**	**HebH3_S**	**Heb50_B**	**Joh49_S**	**Joh49_B**	**Kom45_S**	**Kom45_B**	**Bush Is**	**Nachvak**	**Aku37**	**Aku43**	**Aku54**	**Aku55**	**Aku57**
0.91	0.90	0.65	0.79	0.86	0.86	0.86	0.89	0.84	0.80	0.81	0.92	0.82	0.73	0.81

* Normalized values

m/z **1844**	**Ava12**	**Ava13**	**Ava14**	**Ava16**	**Ava18**	**Ava19**	**Ava21**	**Ava22**	**Ava25**	**Ava26_B**	**Ava26_S**	**Ava27**	**Ava32**	**Ava33**
**N0/N1***	0.59	0.77	0.77	0.80	0.78	0.79	0.79	0.81	0.81	0.75	0.80	0.77	0.86	0.75
**Ava34**	**Ava35**	**HebH3_S**	**Heb50_B**	**Joh49_S**	**Joh49_B**	**Kom45_S**	**Kom45_B**	**Bush Is**	**Nachvak**	**Aku37**	**Aku43**	**Aku54**	**Aku55**	**Aku57**
0.85	0.78	0.59	0.67	NA	NA	0.80	0.82	0.81	0.67	0.74	0.78	0.80	0.65	0.77

* Normalized values

m/z **2063**	**Ava12**	**Ava13**	**Ava14**	**Ava16**	**Ava18**	**Ava19**	**Ava21**	**Ava22**	**Ava25**	**Ava26_B**	**Ava26_S**	**Ava27**	**Ava32**	**Ava33**
**N0/N1***	0.29	0.45	0.44	0.32	0.50	0.42	0.37	0.33	0.39	0.50	0.39	0.42	0.56	0.58
**Ava34**	**Ava35**	**HebH3_S**	**Heb50_B**	**Joh49_S**	**Joh49_B**	**Kom45_S**	**Kom45_B**	**Bush Is**	**Nachvak**	**Aku37**	**Aku43**	**Aku54**	**Aku55**	**Aku57**
0.65	0.43	0.28	0.37	NA	NA	0.42	0.56	0.49	0.30	0.40	0.53	0.54	0.29	0.38

* Normalized values

REFERENCES

1. Plumet P, Gangloff P. Contribution à l'archéologie et l'ethnohistoire de l'Ungava oriental. Côte est, Killiniq, îles Button, Labrador septentrional. Montréal: Presses de l'Université du Québec; 1990.
2. Jordan RH. Preliminary Results from Archaeological Investigations on Avayalik Island, Extreme Northern Labrador. Arctic. 1980;33(3):607-27.
3. Fitzhugh W, Jordan RH, J A, Laeyendecker D. Cordage and Wood from the Avayalik Dorset Site in Northern Labrador. In: Arneborg J, Grønnow B, editors. Dynamics of Northern Societies, Proceedings of the SILA/NABO Conference on Arctic and North Atlantic Archaeology, Copenhagen. Aarhus: Aarhus Universitetsforlag; 2006. p. 153-75.
4. Cox SL, Spiess A. Dorset Settlement and Subsistence in Northern Labrador. Arctic. 1980;33(3):659-69.
5. Fitzhugh W. Preliminary Report on the Torngat Archaeological Project. Arctic. 1980;33(3):585-606.
6. Kaplan SA. Neo-Eskimo Occupations of the Northern Labrador Coast. Arctic. 1980;33(3):646-58.
7. Whitridge P. Nachvak Fjord, Summary of 2003 Fieldwork. Provincial Archaeology Office Newsletter. 2004;2(1).
8. Whitridge P. Nachvak Fjord, Summary of 2004 Fieldwork. Provincial Archaeology Office Newsletter. 2005;3.
9. Rankin LK. An archaeological view of the Thule/Inuit occupation of Labrador. St. John's, Newfoundland and Labrador, Canada: Memorial University, 2009.
10. Kaplan SA. Economic and social change in Larbador Neo-Eskimo culture. Part II [PhD dissertation]: Bryn Mawr College; 1983.
11. Loring S, Arendt B. "…They Gave Hebron, The City of Refuge…" (Joshua 21:13): An Archaeological Reconnaissance at Hebron, Labrador. JONA. 2009;2(sp 1):33-56. doi: 10.3721/037.002.s106.

12. Perez Hurtado P, O'Connor PB. Deamidation of Collagen. Analytical Chemistry. 2012;84(6):3017-25. doi: 10.1021/ac202980z.
13. Wilson J, van Doorn NL, Collins MJ. Assessing the Extent of Bone Degradation Using Glutamine Deamidation in Collagen. Analytical Chemistry. 2012;84(21):9041-8. doi: 10.1021/ac301333t.
14. van Doorn NL, Wilson J, Hollund H, Soressi M, Collins MJ. Site-specific deamidation of glutamine: a new marker of bone collagen deterioration. Rapid Communications in Mass Spectrometry. 2012;26(19):2319-27. doi: 10.1002/rcm.6351.
15. Welker F, Soressi MA, Roussel M, van Riemsdijk I, Hublin J-J, Collins MJ. Variations in glutamine deamidation for a Châtelperronian bone assemblage as measured by peptide mass fingerprinting of collagen. STAR: Science & Technology of Archaeological Research. 2017;3(1):15-27. doi: 10.1080/20548923.2016.1258825.
16. Schroeter ER, Cleland TP. Glutamine deamidation: an indicator of antiquity, or preservational quality? Rapid Communications in Mass Spectrometry. 2016;30(2):251-5. doi: 10.1002/rcm.7445.
17. Simpson JP, Penkman KEH, Demarchi B, Koon H, Collins MJ, Thomas-Oates J, et al. The effects of demineralisation and sampling point variability on the measurement of glutamine deamidation in type I collagen extracted from bone. Journal of Archaeological Science. 2016;69:29-38. doi: https://doi.org/10.1016/j.jas.2016.02.002.
18. Solazzo C, Rogers PW, Weber L, Beaubien HF, Wilson J, Collins M. Species identification by peptide mass fingerprinting (PMF) in fibre products preserved by association with copper-alloy artefacts. J Archaeol Sci. 2014;49:524-35. doi: 10.1016/j.jas.2014.06.009.
19. Solazzo C, Wilson J, Dyer JM, Clerens S, Plowman JE, von Holstein I, et al. Modeling Deamidation in Sheep α-Keratin Peptides and Application to Archeological Wool Textiles. Analytical Chemistry. 2014;86(1):567-75. doi: 10.1021/ac4026362.
