# Supplementary material for: Molecular markers in keratins from Mysticeti whales for species identification of baleen in museum and archaeological collections
Source: PLoS One. 2017 Aug 30;12(8):e0183053. doi: 10.1371/journal.pone.0183053 (PMC5576650; doi:10.1371/journal.pone.0183053)

Supporting Information File S4

Peptide mass fingerprinting of reference materials and MS/MS spectra

MS spectrum *Eubalaena japonica* 339990 p2
MS spectrum *Eubalaena glacialis* 504257 p3
MS spectrum *Balaena mysticetus* 571338 p4
MS spectrum *Balaenoptera brydei* 504074 North Atlantic p5
MS spectrum *Balaenoptera brydei* 504688 South Pacific p6
MS spectrum *Balaenoptera brydei* 504689 South Pacific p7
MS spectrum *Balaenoptera borealis* 504998 p8
MS spectrum *Balaenoptera musculus* 269541 p9
MS spectrum *Balaenoptera acutorostrata* 239305 p10
MS spectrum *Balaenoptera physalus* 275769 p11
MS spectrum *Megaptera novaeangliae* 504216 p12
MS spectrum *Megaptera novaeangliae* 267999 p13
MS spectrum *Eschrichtius robustus* 572613 p14

MS/MS spectrum of YSSQLAQIQGLISNVEAQLSEIR in *B. brydei* 504689 p15
MS/MS spectrum of YSSQLAQIQGLIGNVEAQLSEIR in *E. robustus* 504999 p16
MS/MS spectrum of YTSQLAQIQCLISNVEAQLSEIR in *E. japonica* 339990 p17
MS/MS spectrum of APYISSVPCAPAPQLSTQIR in *B. brydei* 504074 p18
MS/MS spectrum of VPYISSVPCAPAPQLSTQIR in *E. japonica* 339990 p19

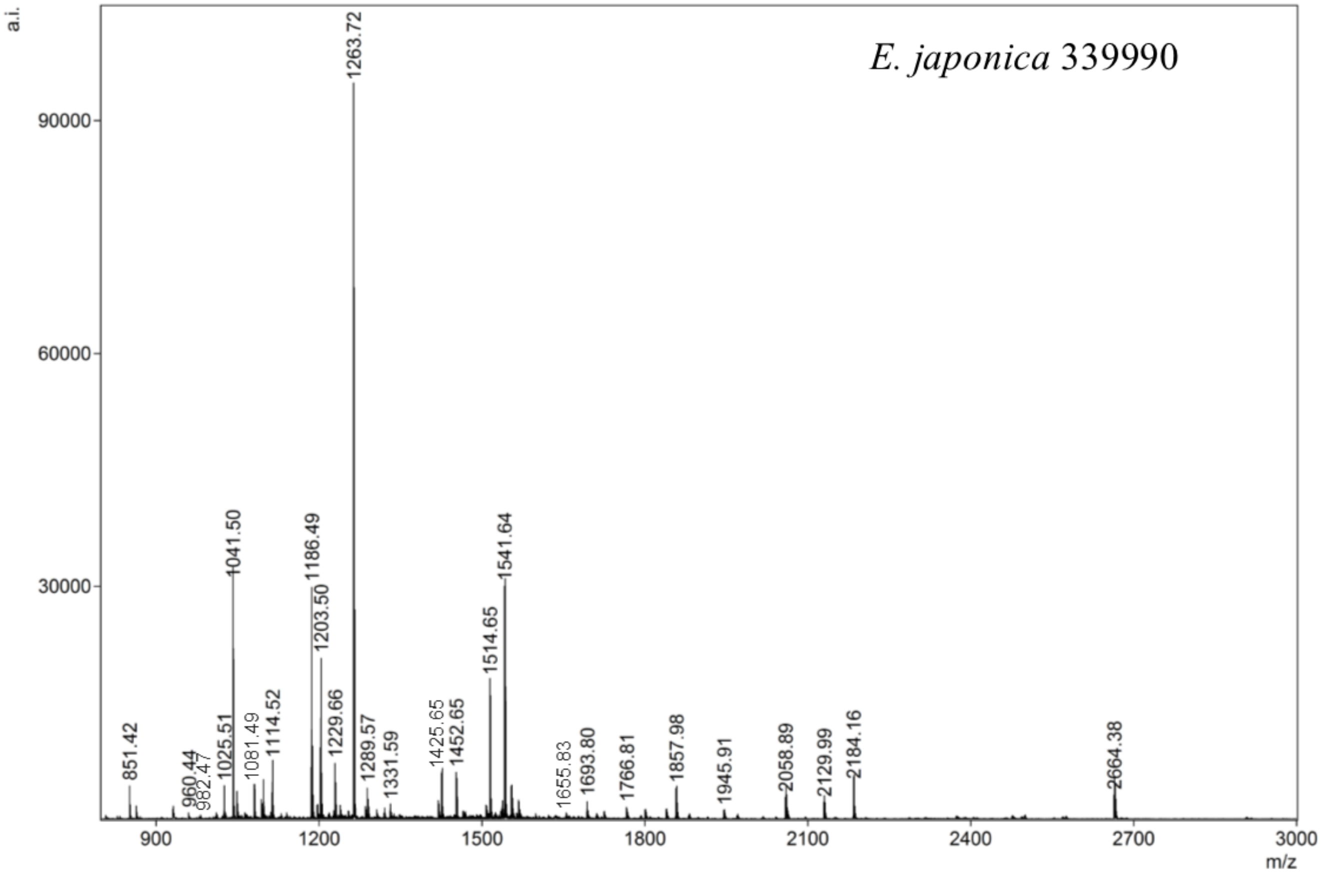
E. japonica 339990
a.i.
90000
60000
30000
851.42
960.44
982.47
1025.51
1041.50
1081.49
1114.52
1186.49
1203.50
1229.66
1263.72
1289.57
1331.59
1425.65
1452.65
1514.65
1541.64
1655.83
1693.80
1766.81
1857.98
1945.91
2058.89
2129.99
2184.16
2664.38
900
1200
1500
1800
2100
2400
2700
3000
m/z

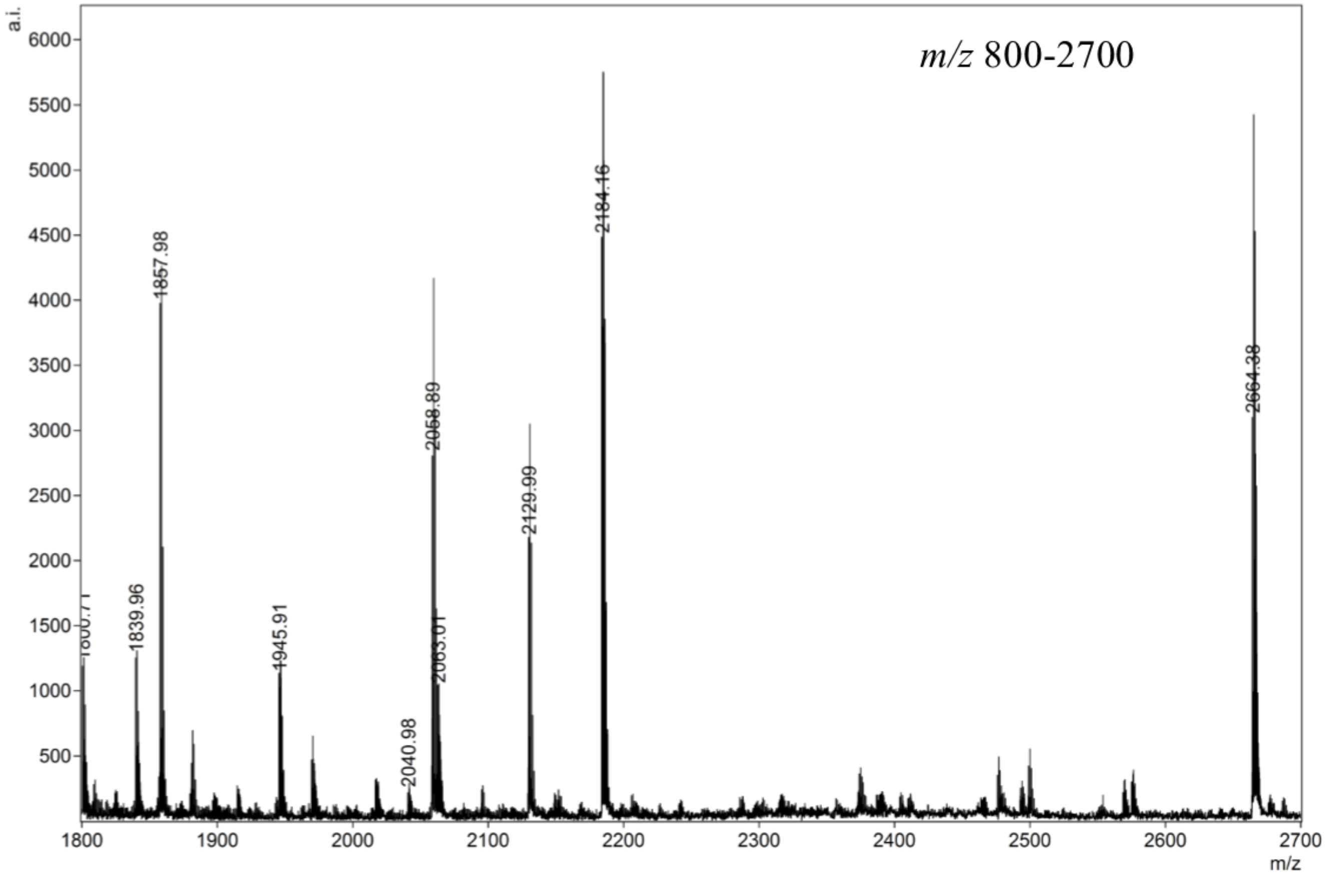
m/z 800-2700
a.i.
6000
5500
5000
4500
4000
3500
3000
2500
2000
1500
1000
500
1839.96
1857.98
1945.91
2040.98
2058.89
2129.99
2184.16
2664.38
1800
1900
2000
2100
2200
2300
2400
2500
2600
2700
m/z

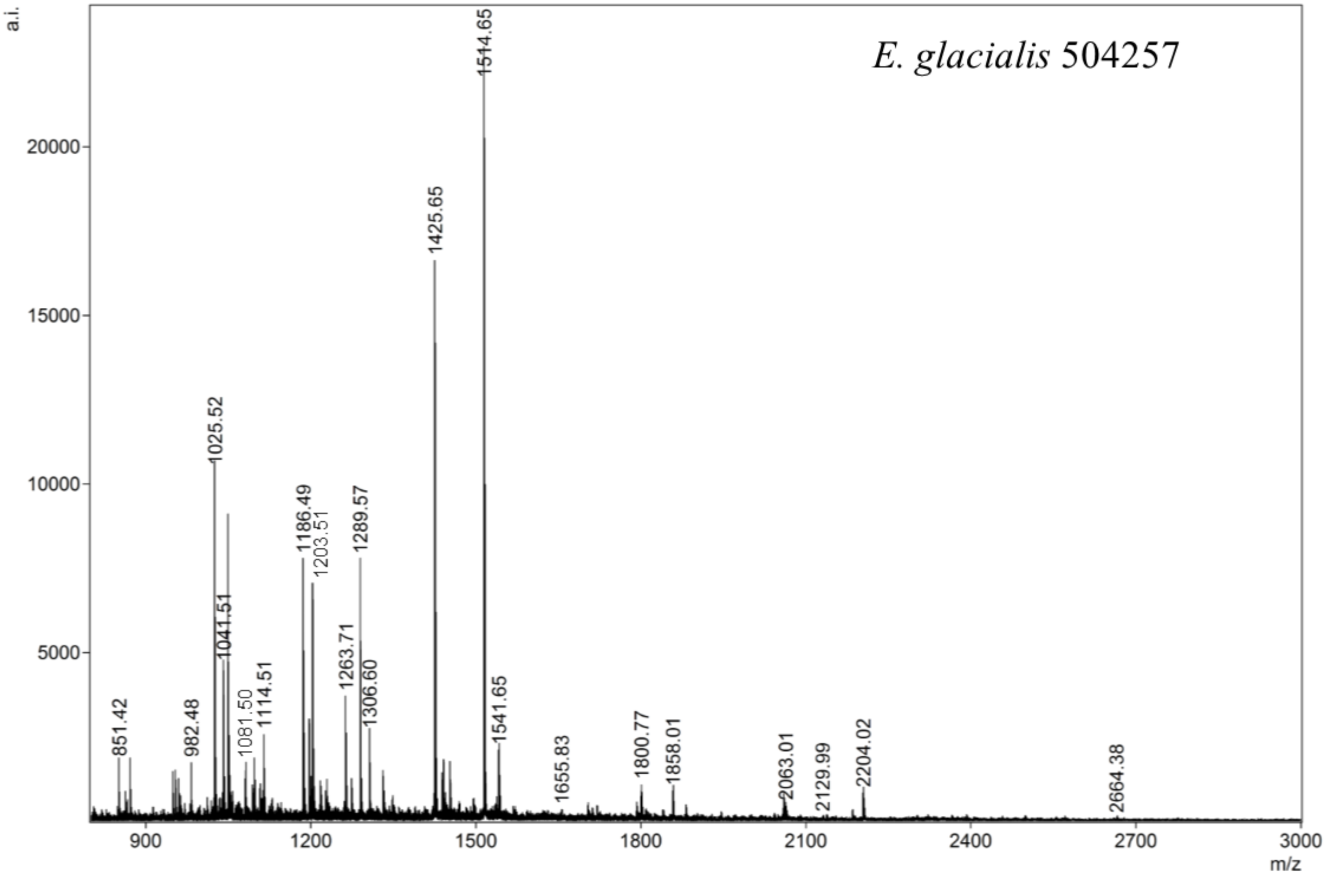
E. glacialis 504257
a.i.
m/z
851.42
982.48
1025.52
1041.51
1081.50
1114.51
1186.49
1203.51
1263.71
1289.57
1306.60
1425.65
1514.65
1541.65
1655.83
1800.77
1858.01
2063.01
2129.99
2204.02
2664.38

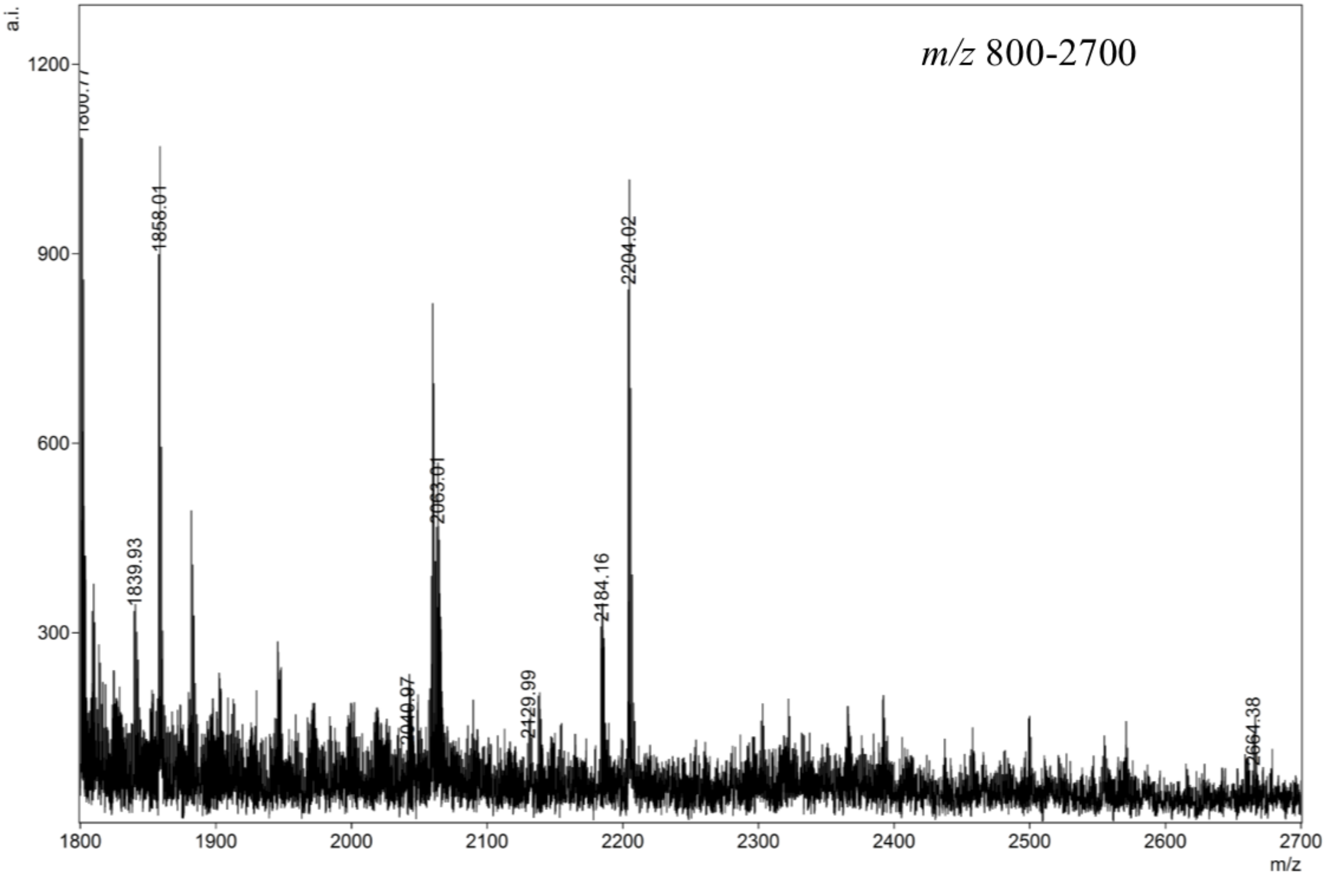
m/z 800-2700
a.i.
m/z
1858.01
1839.93
2040.97
2063.01
2129.99
2184.16
2204.02
2664.38

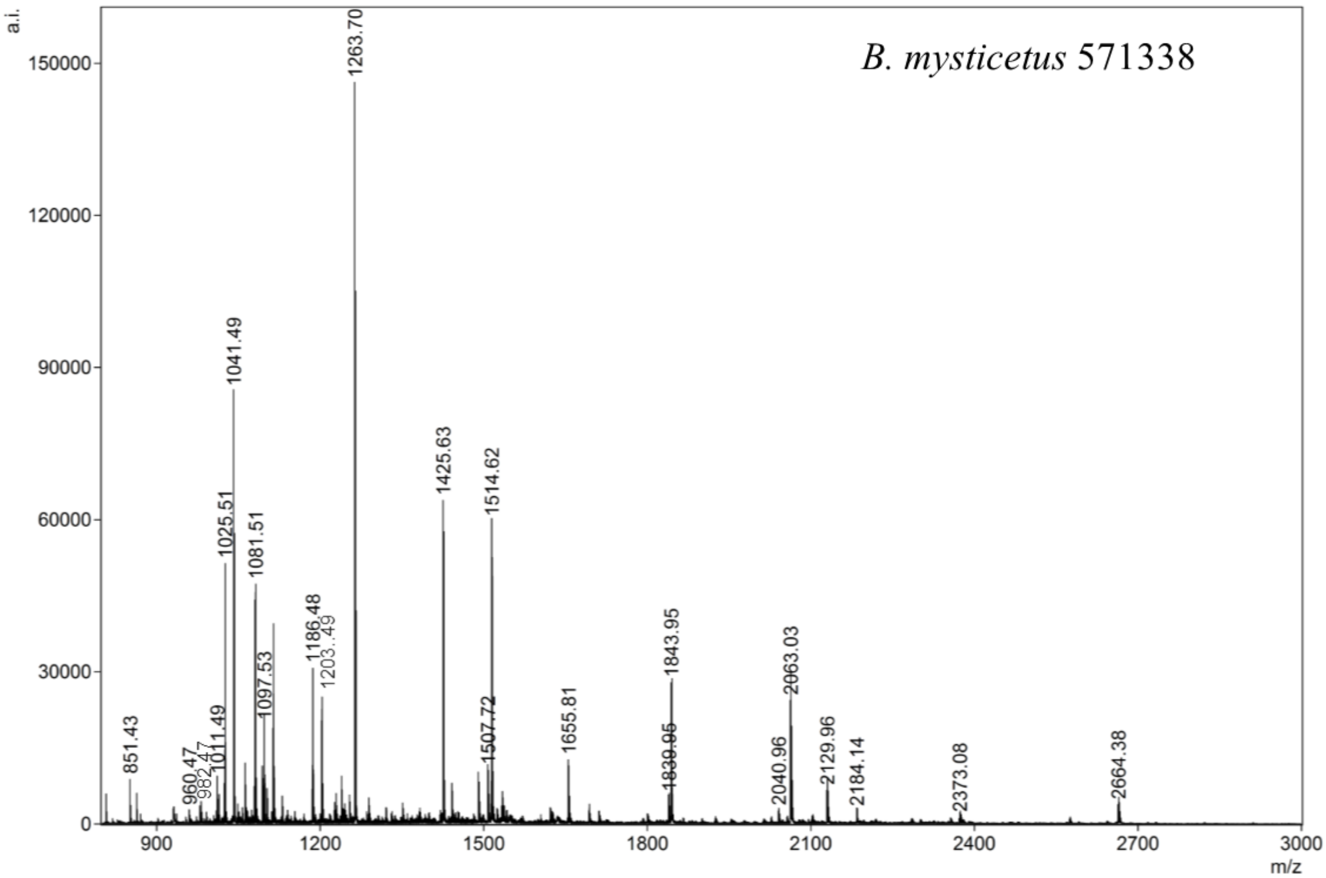
B. mysticetus 571338
a.i.
150000
120000
90000
60000
30000
0
900
1200
1500
1800
2100
2400
2700
3000
m/z
1263.70
1041.49
1425.63
1514.62
1025.51
1081.51
1186.48
1203.49
1097.53
1843.95
2063.03
851.43
960.47
982.47
1011.49
1507.72
1655.81
1839.95
2040.96
2129.96
2184.14
2373.08
2664.38

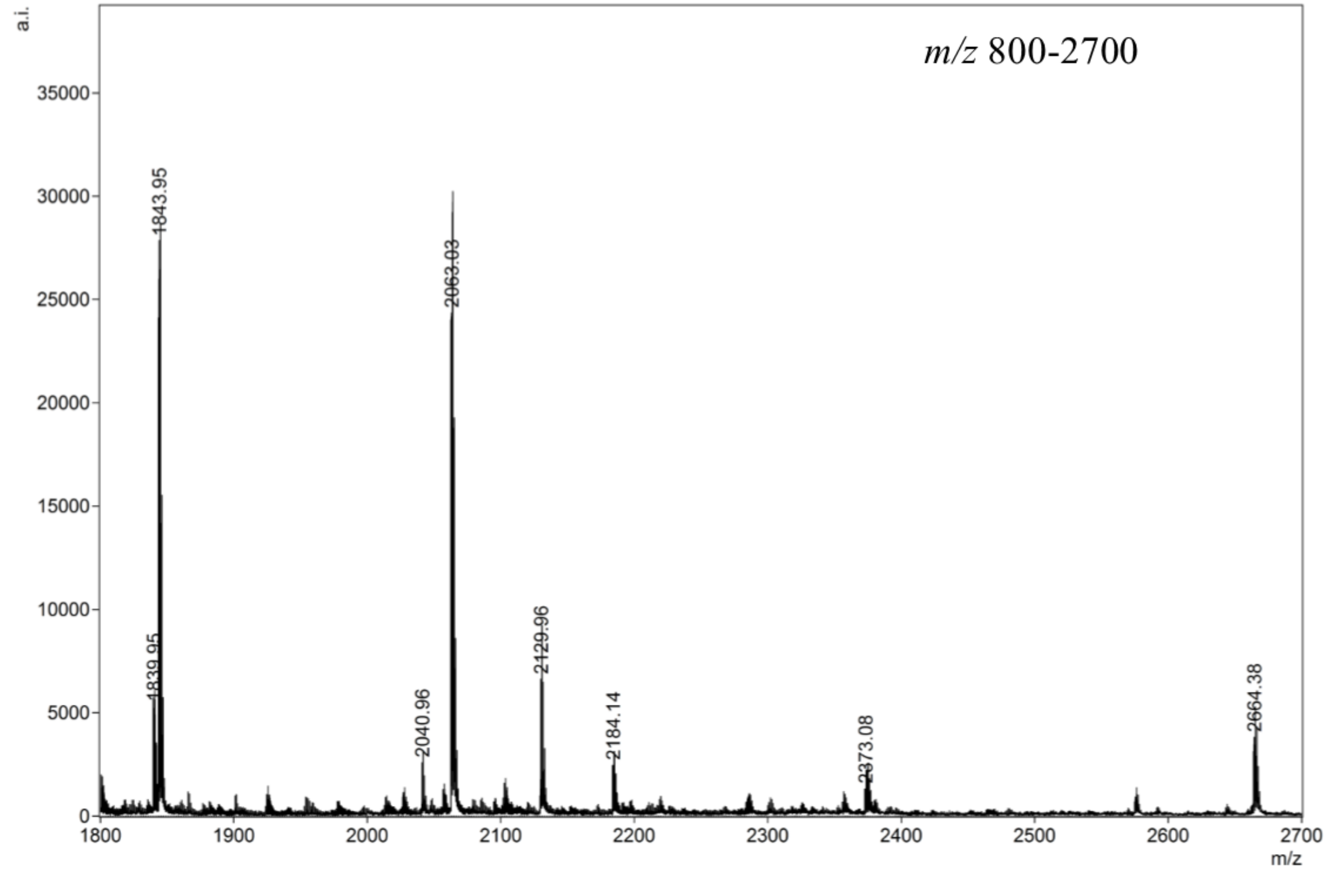
m/z 800-2700
a.i.
35000
30000
25000
20000
15000
10000
5000
0
1800
1900
2000
2100
2200
2300
2400
2500
2600
2700
m/z
1843.95
2063.03
1839.95
2040.96
2129.96
2184.14
2373.08
2664.38

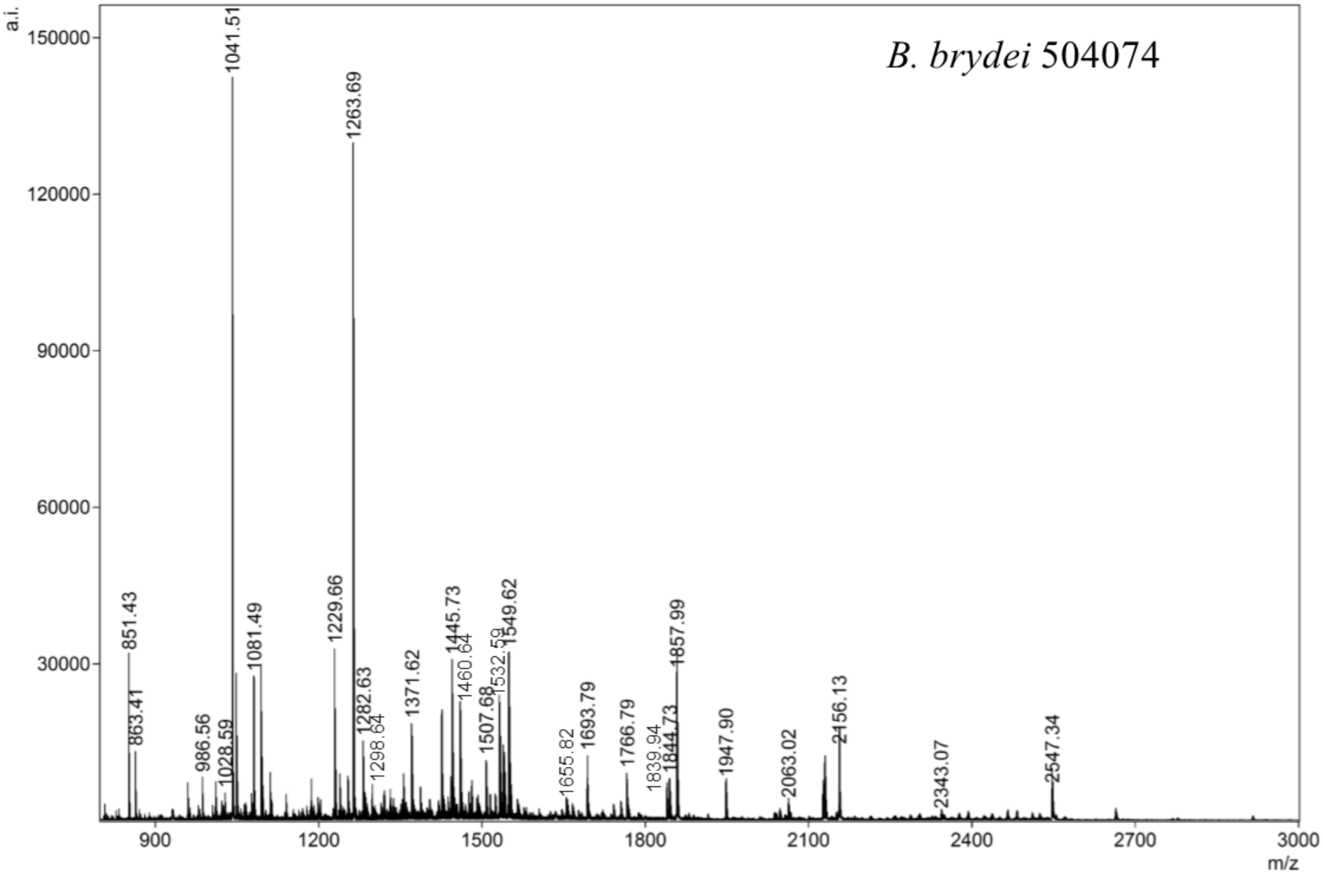
B. brydei 504074
a.i.
150000
120000
90000
60000
30000
1041.51
1263.69
851.43
863.41
986.56
1028.59
1081.49
1229.66
1282.63
1298.64
1371.62
1445.73
1460.64
1507.68
1532.59
1549.62
1655.82
1693.79
1766.79
1839.94
1844.73
1857.99
1947.90
2063.02
2156.13
2343.07
2547.34
900
1200
1500
1800
2100
2400
2700
3000
m/z

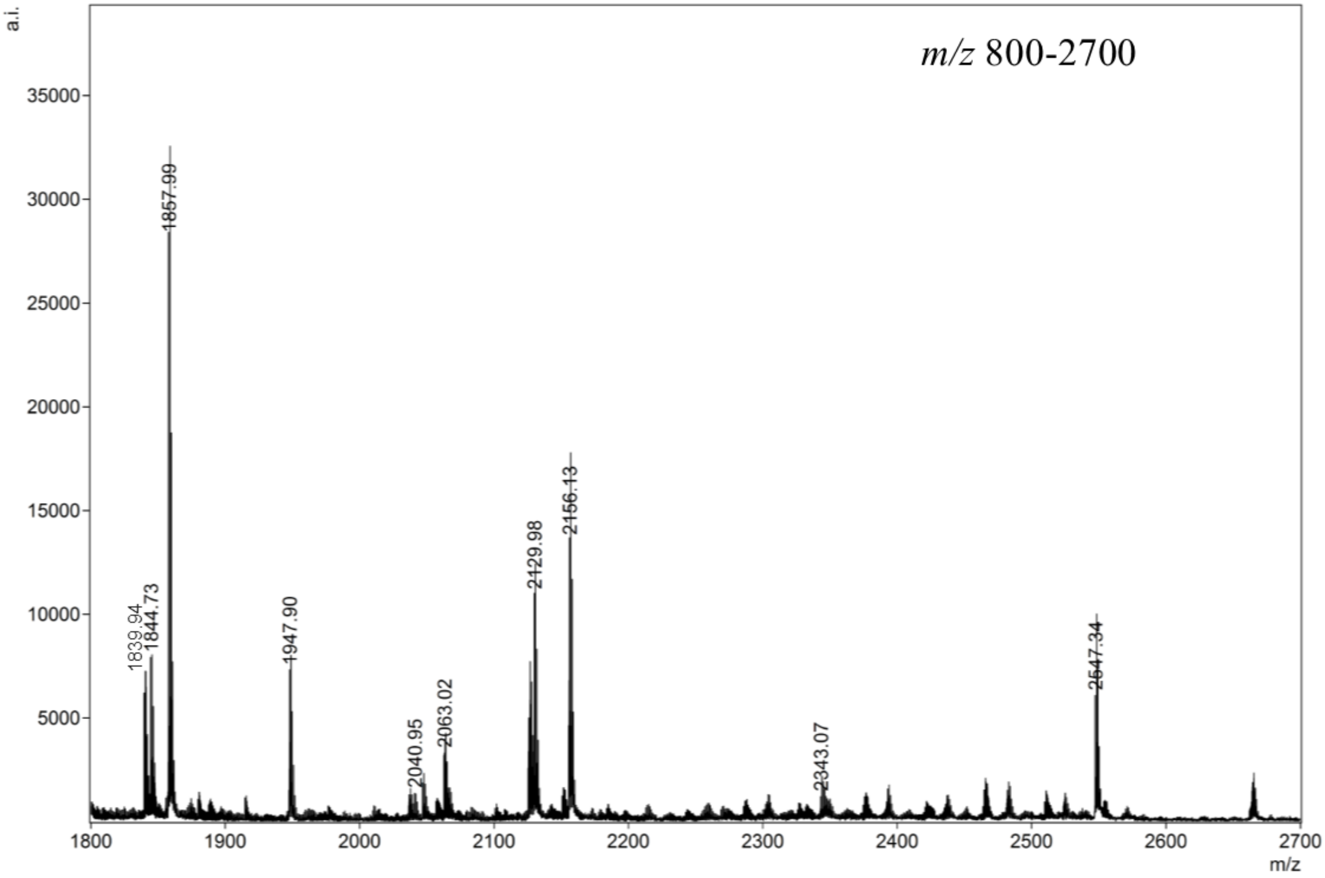
m/z 800-2700
a.i.
35000
30000
25000
20000
15000
10000
5000
1857.99
1839.94
1844.73
1947.90
2040.95
2063.02
2129.98
2156.13
2343.07
2547.34
1800
1900
2000
2100
2200
2300
2400
2500
2600
2700
m/z

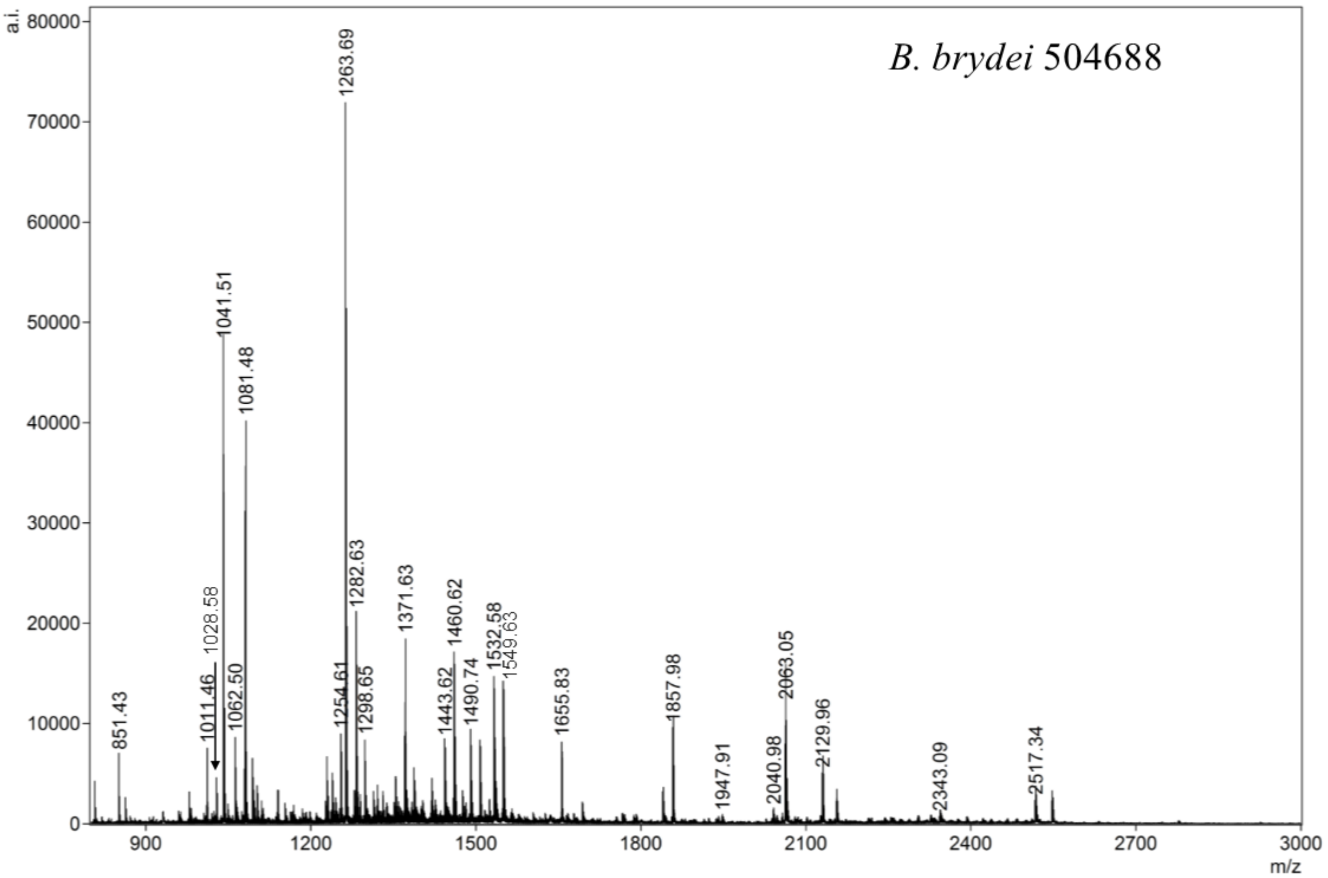
B. brydei 504688
a.i.
m/z
851.43
1011.46
1028.58
1041.51
1062.50
1081.48
1254.61
1263.69
1282.63
1298.65
1371.63
1443.62
1460.62
1490.74
1532.58
1549.63
1655.83
1857.98
1947.91
2040.98
2063.05
2129.96
2343.09
2517.34

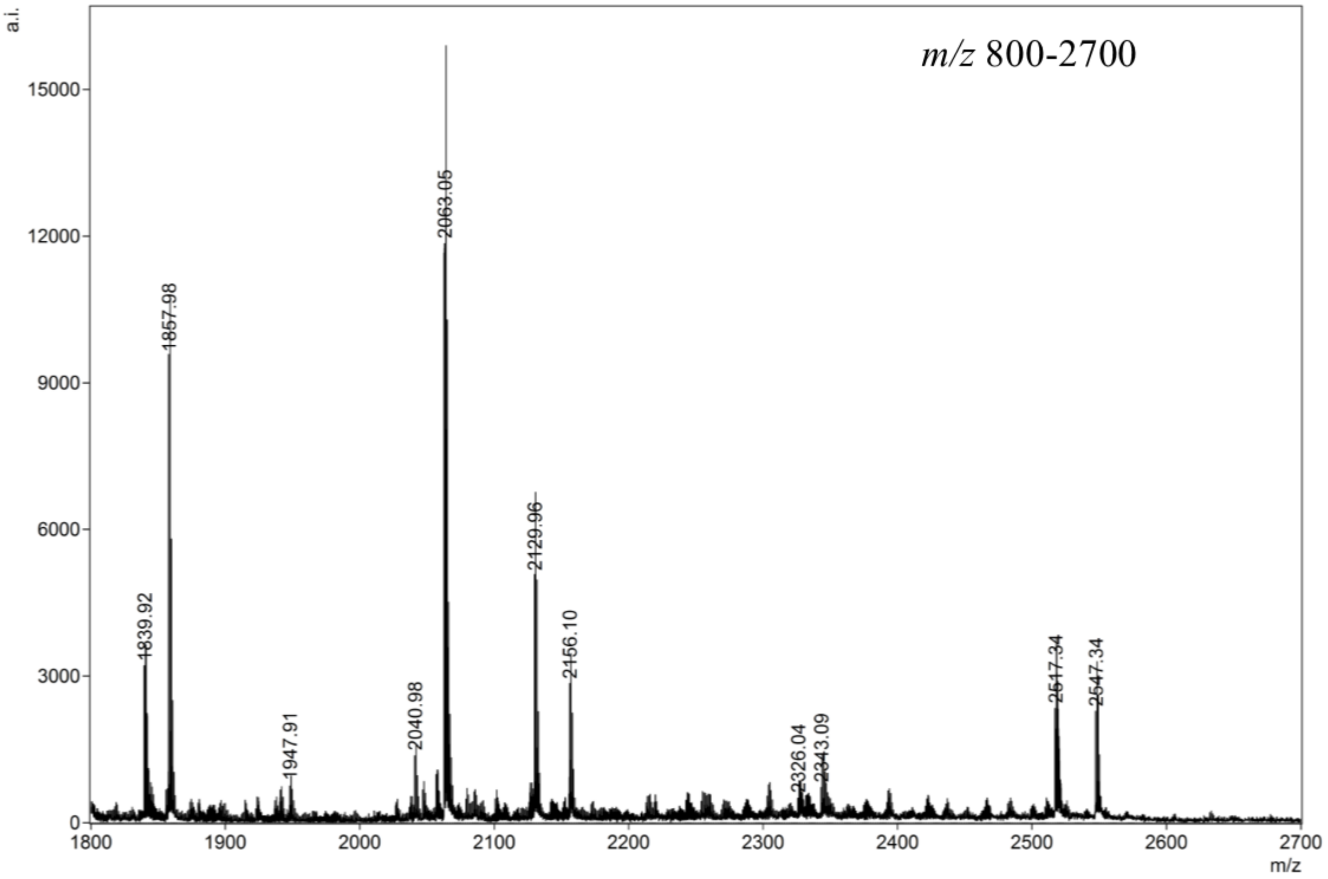
m/z 800-2700
a.i.
m/z
1839.92
1857.98
1947.91
2040.98
2063.05
2129.96
2156.10
2326.04
2343.09
2517.34
2547.34

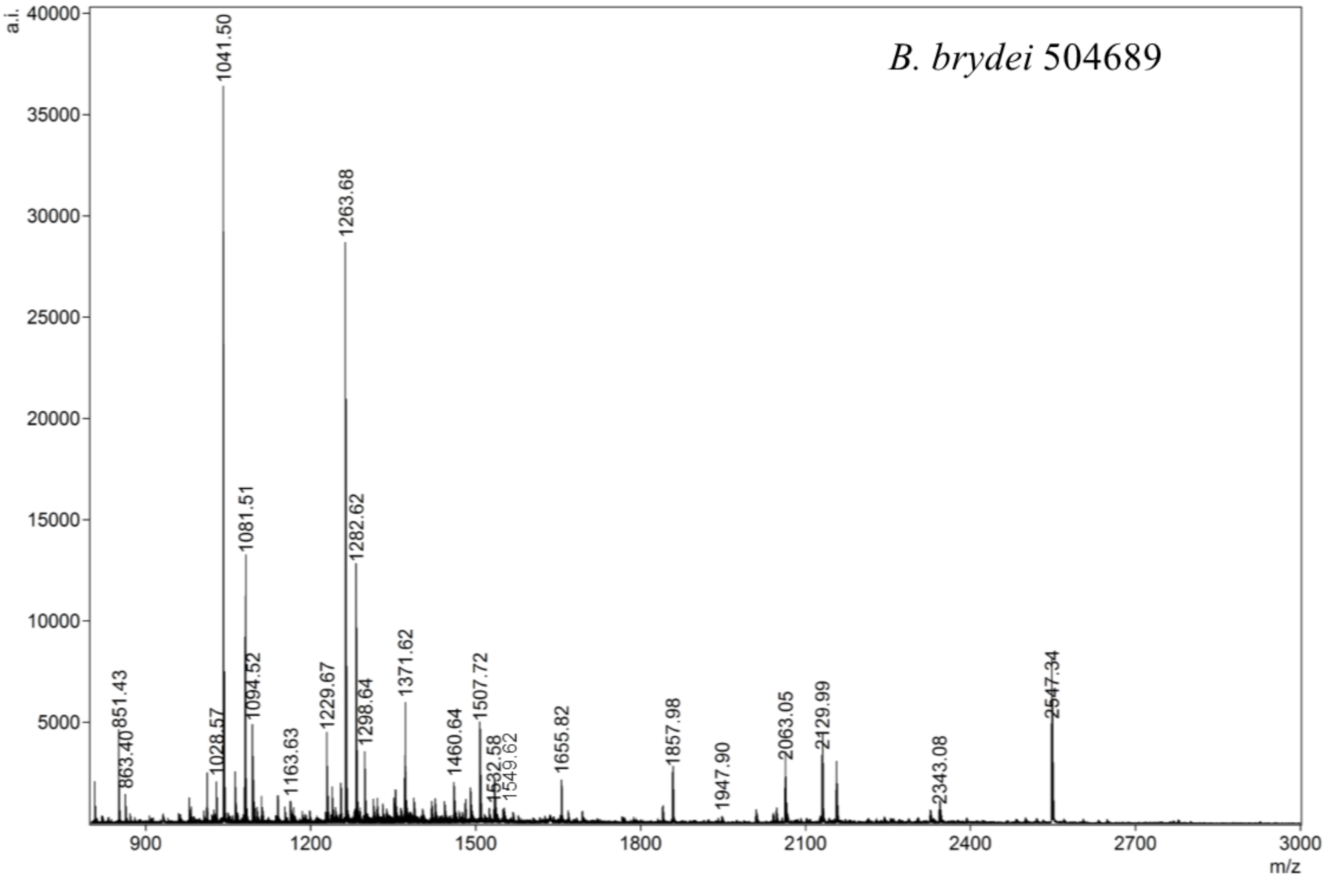
B. brydei 504689
a.i.
m/z
1041.50
1263.68
1081.51
1282.62
1094.52
851.43
863.40
1028.57
1163.63
1229.67
1298.64
1371.62
1460.64
1507.72
1532.58
1549.62
1655.82
1857.98
1947.90
2063.05
2129.99
2343.08
2547.34

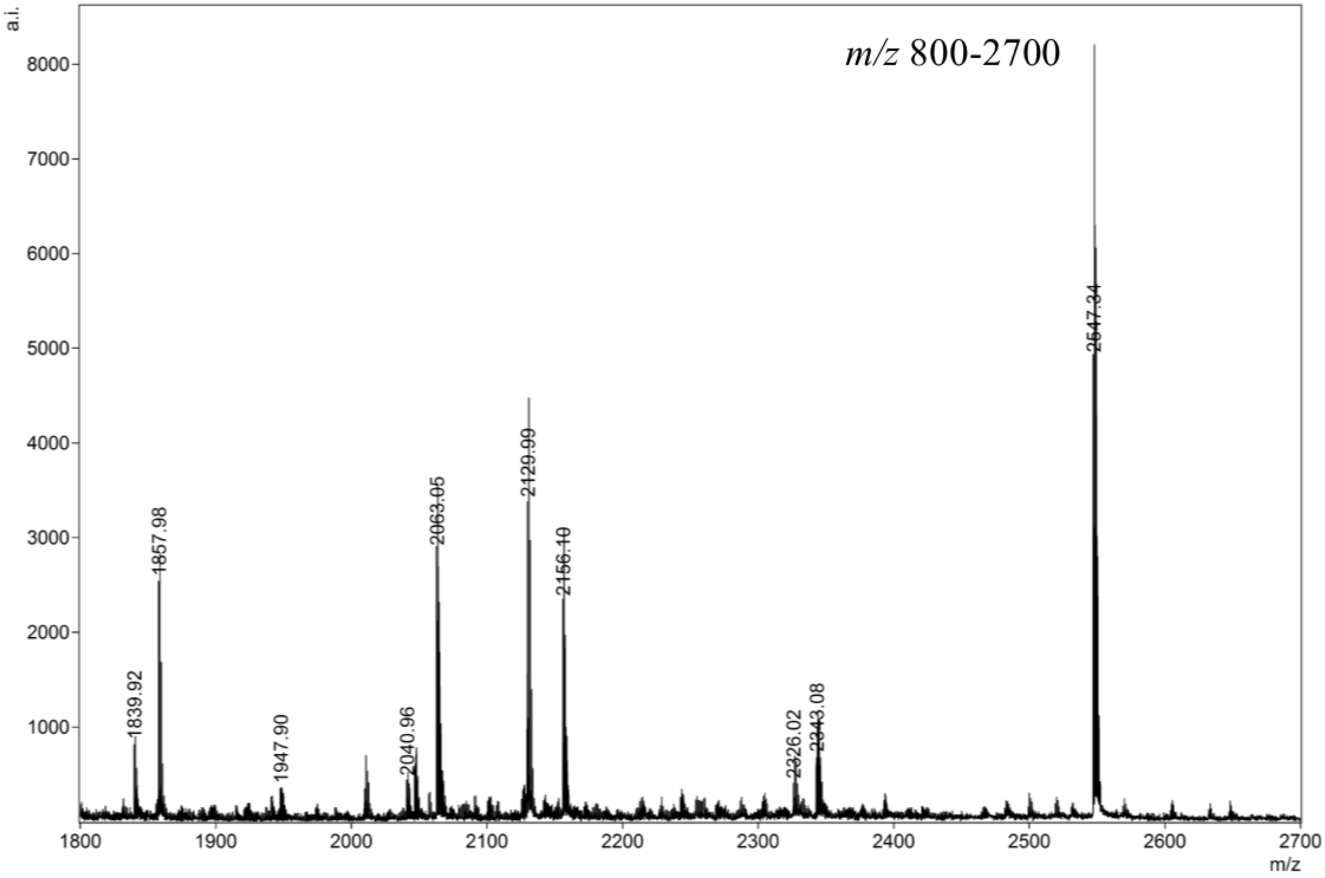
m/z 800-2700
a.i.
m/z
2547.34
2129.99
2063.05
2156.10
1857.98
1839.92
1947.90
2040.96
2326.02
2343.08

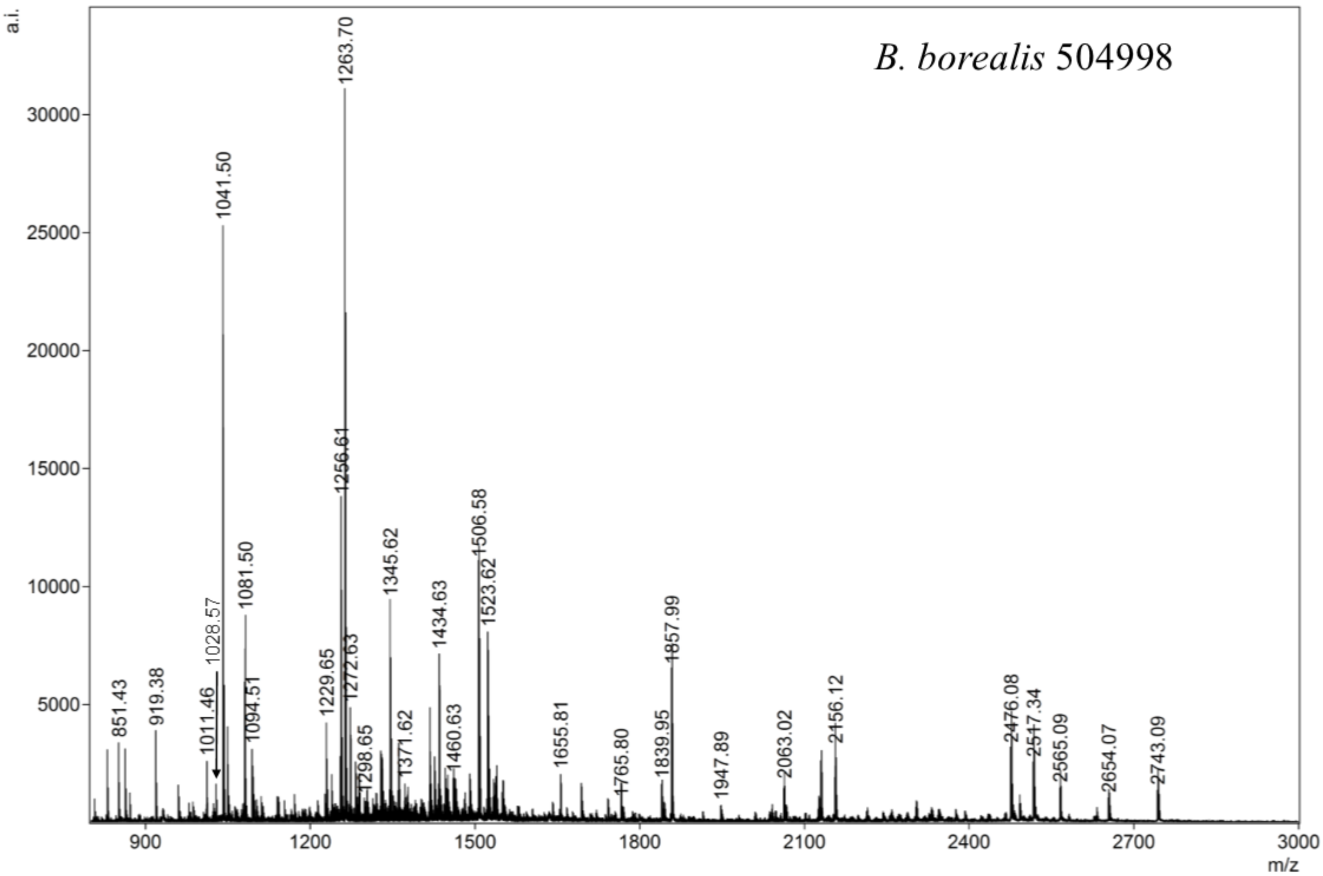
B. borealis 504998
a.i.
m/z
1263.70
1041.50
1256.61
1506.58
1523.62
1345.62
1081.50
1434.63
1857.99
1028.57
1272.63
1229.65
919.38
851.43
1094.51
1011.46
1298.65
1371.62
1460.63
1655.81
1765.80
1839.95
1947.89
2063.02
2156.12
2476.08
2517.34
2565.09
2654.07
2743.09

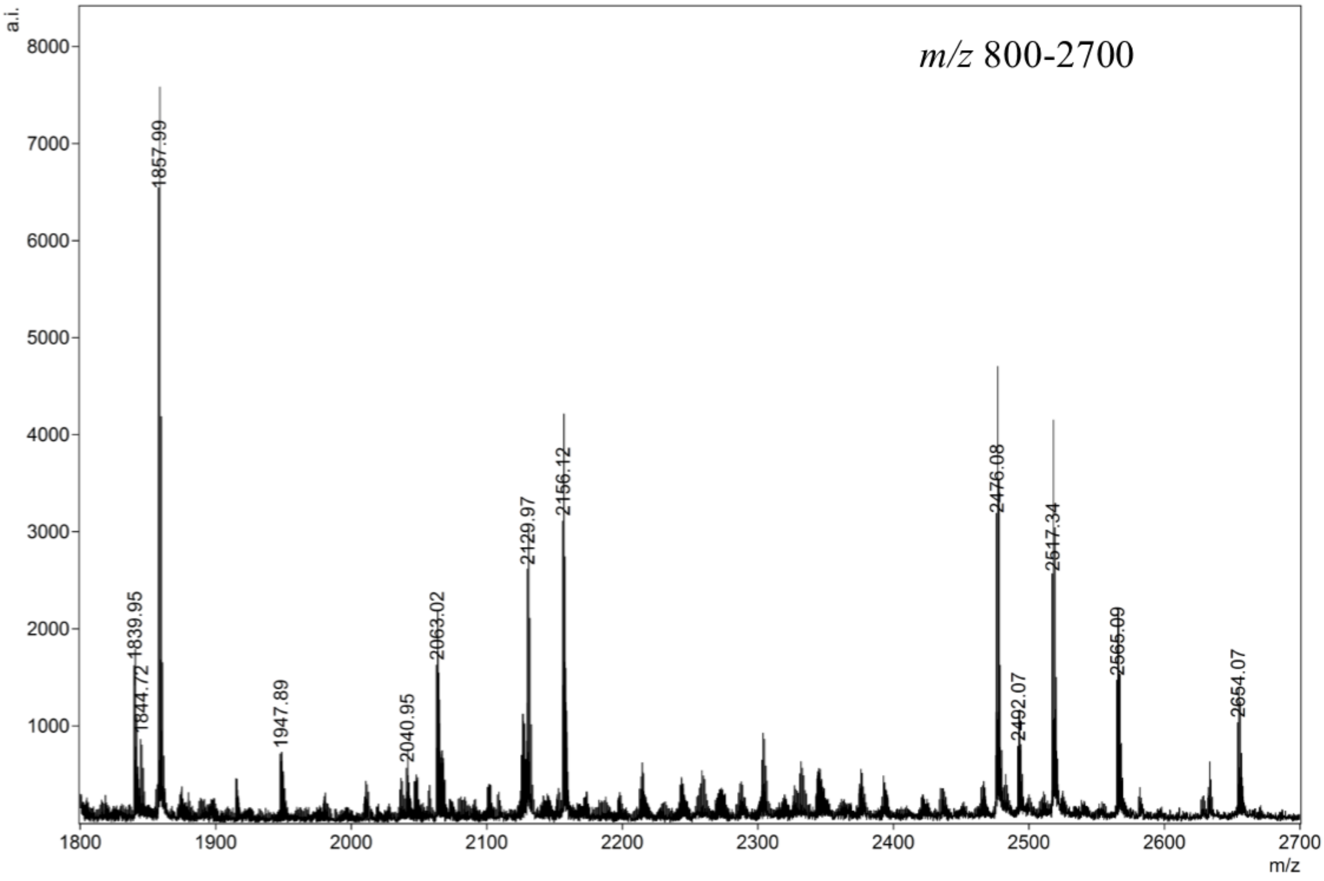
m/z 800-2700
a.i.
m/z
1857.99
1839.95
1844.72
1947.89
2040.95
2063.02
2129.97
2156.12
2476.08
2492.07
2517.34
2565.09
2654.07

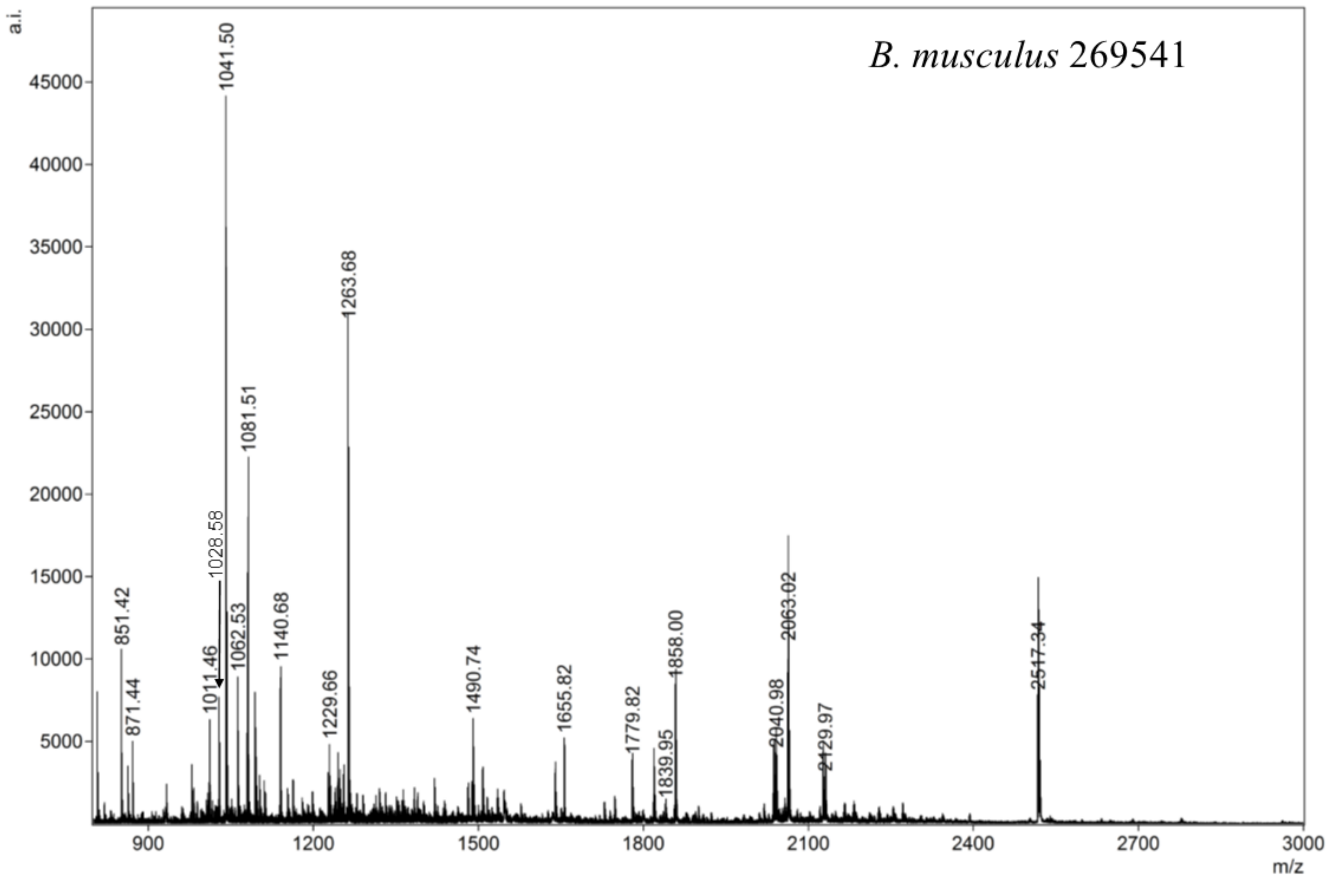
B. musculus 269541
a.i.
45000
40000
35000
30000
25000
20000
15000
10000
5000
1041.50
1263.68
1081.51
1028.58
851.42
871.44
1011.46
1062.53
1140.68
1229.66
1490.74
1655.82
1779.82
1839.95
1858.00
2040.98
2063.02
2129.97
2517.34
900
1200
1500
1800
2100
2400
2700
3000
m/z

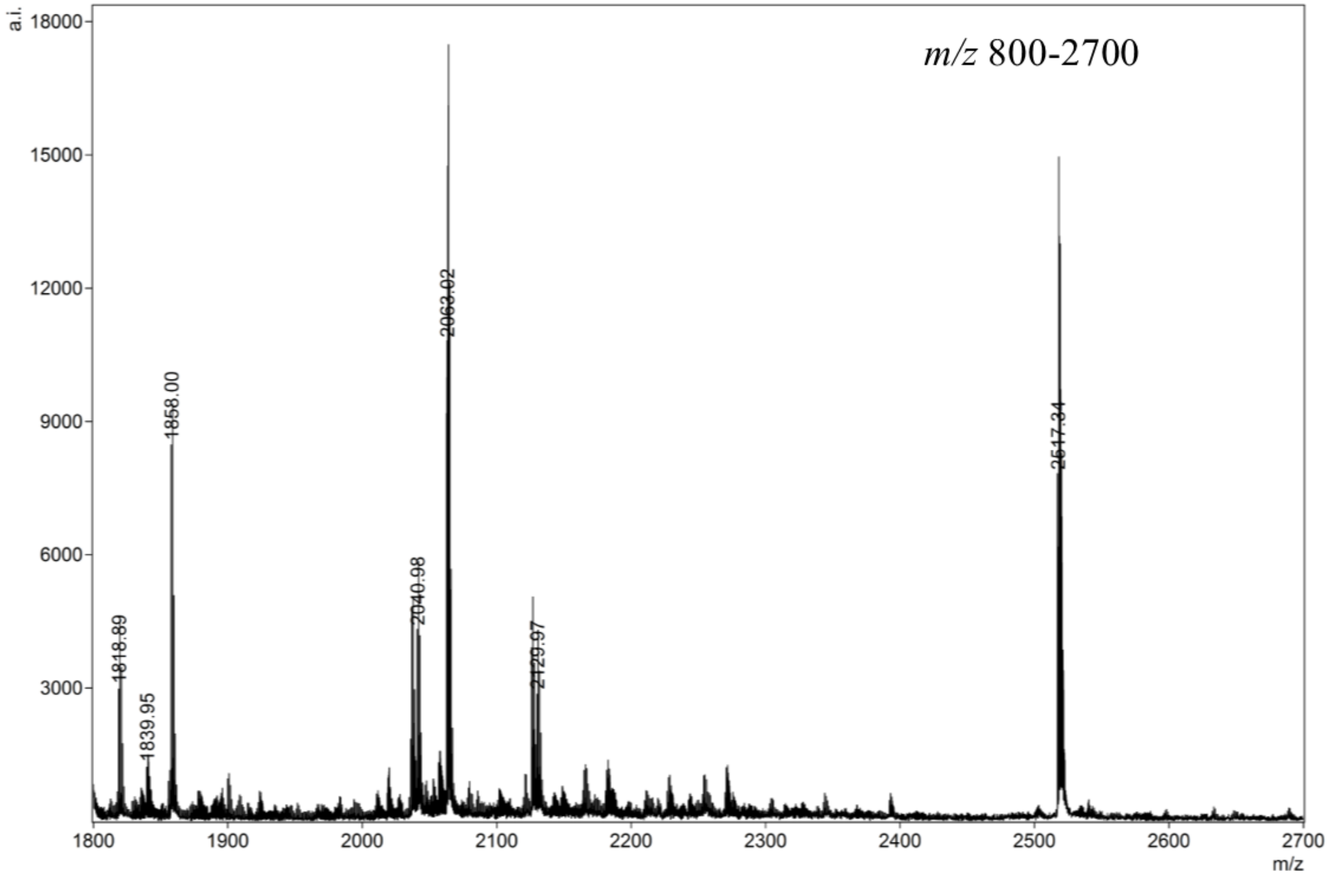
m/z 800-2700
a.i.
18000
15000
12000
9000
6000
3000
1818.89
1839.95
1858.00
2040.98
2063.02
2129.97
2517.34
1800
1900
2000
2100
2200
2300
2400
2500
2600
2700
m/z

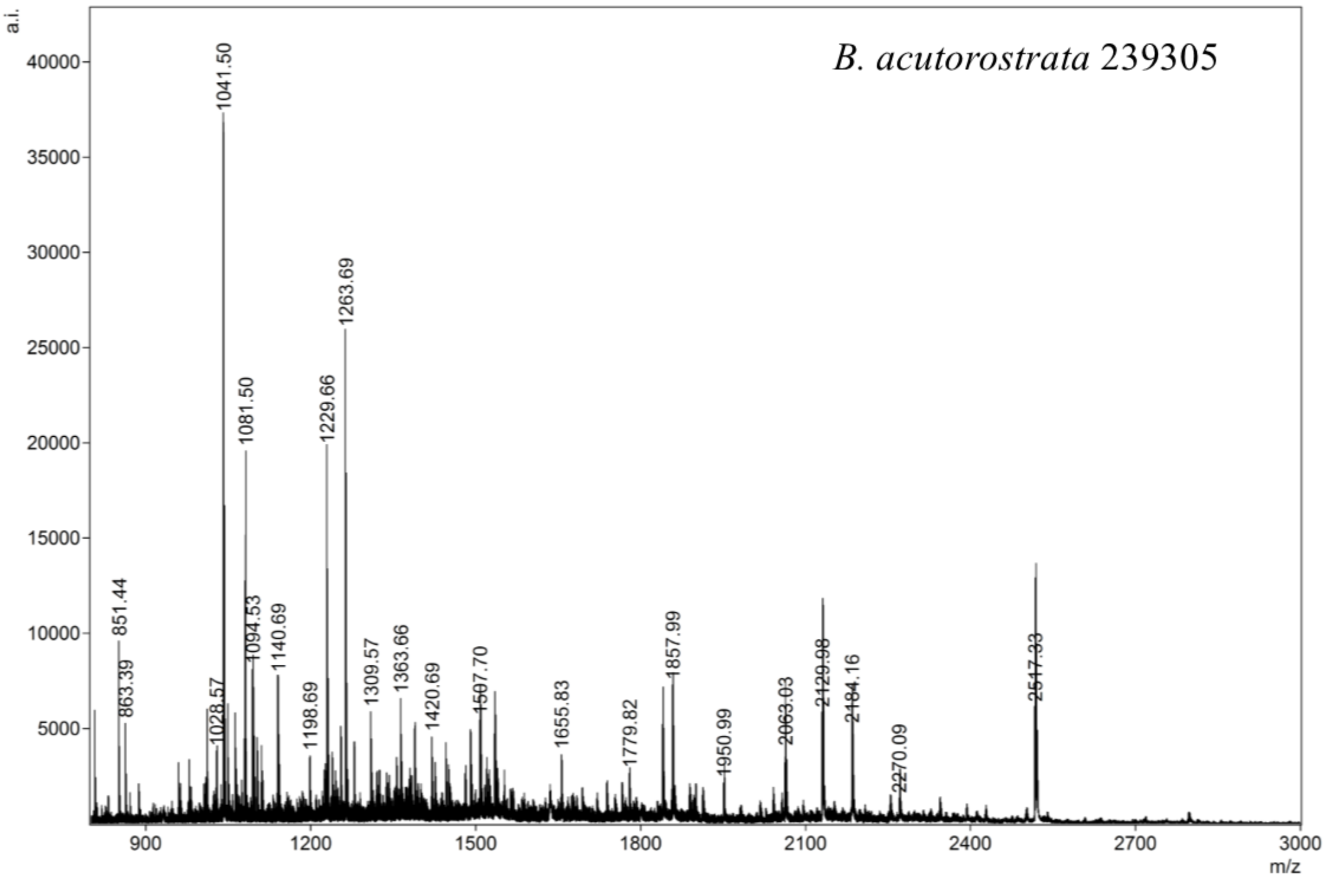
B. acutorostrata 239305
a.i.
40000
35000
30000
25000
20000
15000
10000
5000
1041.50
1263.69
1081.50
1229.66
851.44
863.39
1028.57
1094.53
1140.69
1198.69
1309.57
1363.66
1420.69
1507.70
1655.83
1779.82
1857.99
1950.99
2063.03
2129.98
2184.16
2270.09
2517.33
900
1200
1500
1800
2100
2400
2700
3000
m/z

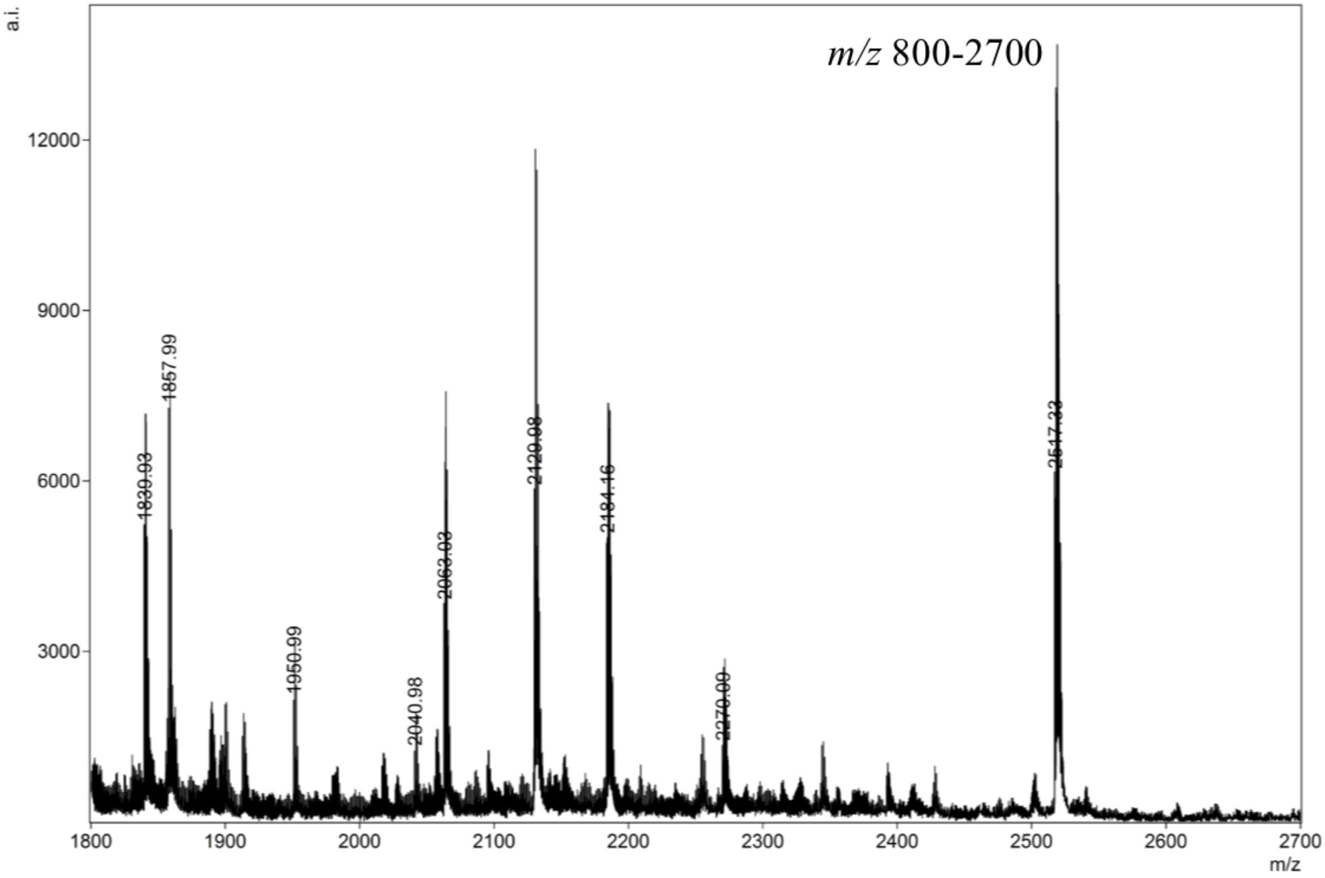
m/z 800-2700
a.i.
12000
9000
6000
3000
1839.93
1857.99
1950.99
2040.98
2063.03
2129.98
2184.16
2270.09
2517.33
1800
1900
2000
2100
2200
2300
2400
2500
2600
2700
m/z

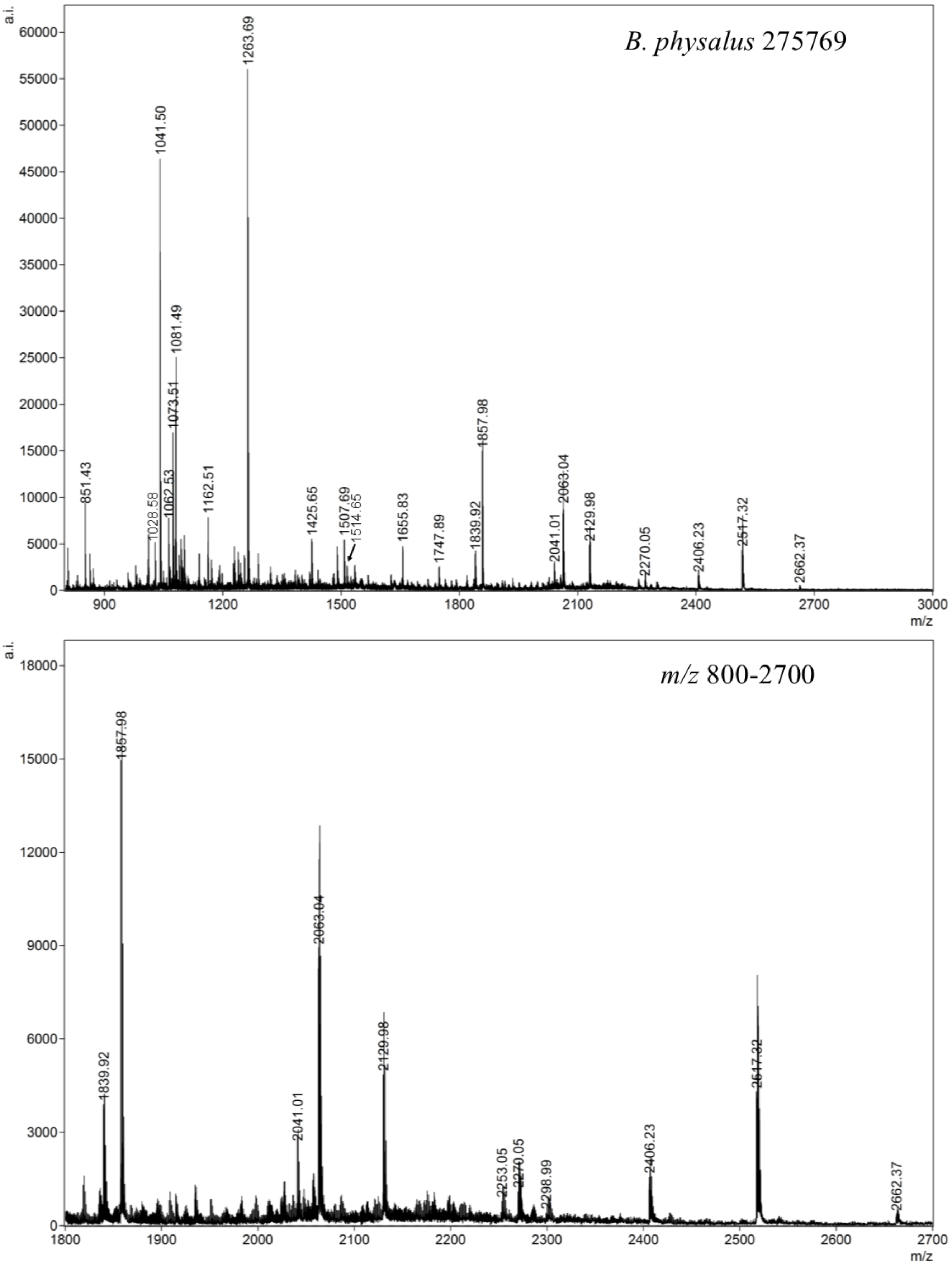
B. physalus 275769
a.i.
m/z
851.43
1028.58
1041.50
1062.53
1073.51
1081.49
1162.51
1263.69
1425.65
1507.69
1514.65
1655.83
1747.89
1839.92
1857.98
2041.01
2063.04
2129.98
2270.05
2406.23
2517.32
2662.37
m/z 800-2700
2253.05
2298.99

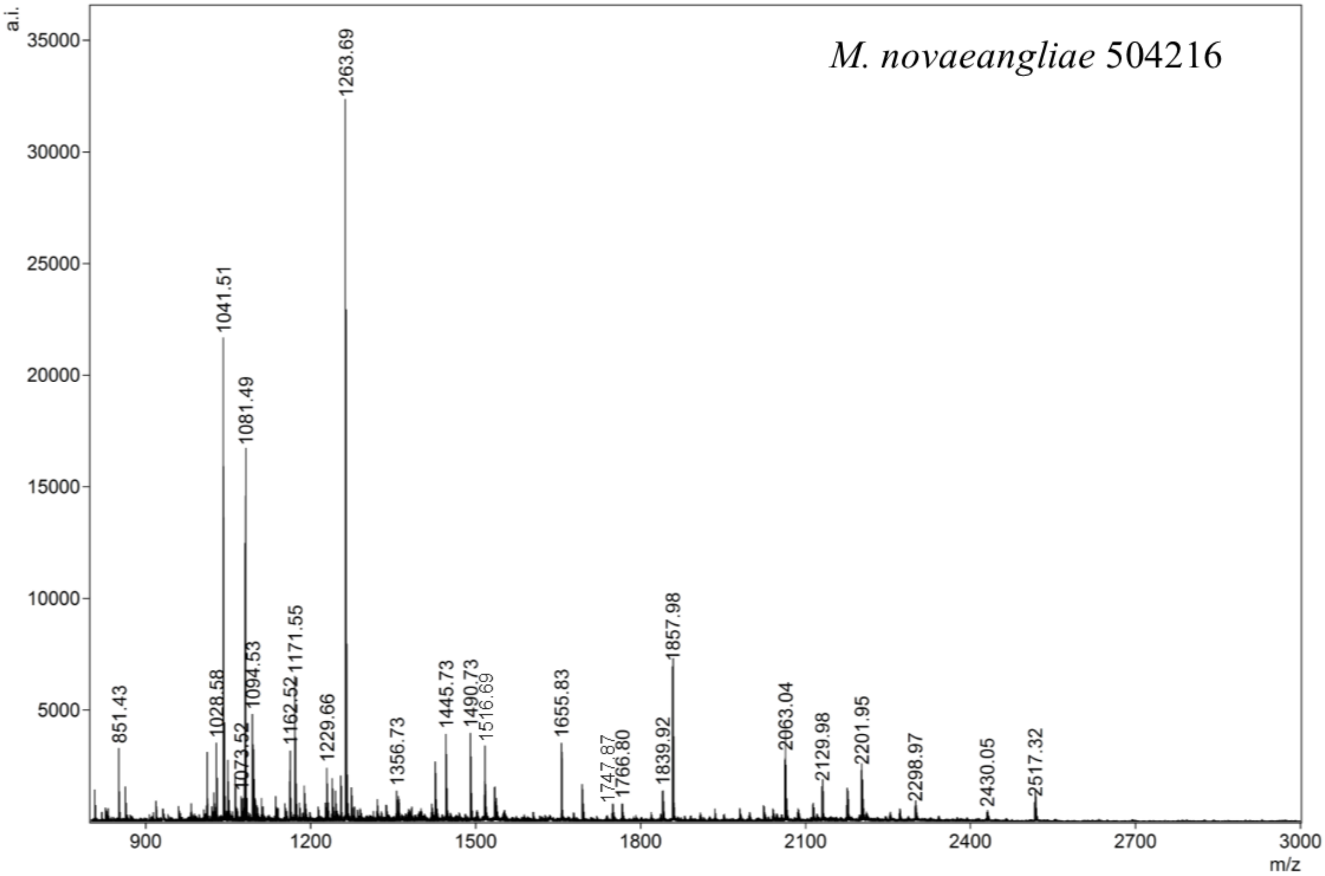

M. novaeangliae 504216
a.i.
m/z
1263.69
1041.51
1081.49
1857.98
1171.55
1094.53
1028.58
1162.52
851.43
1445.73
1490.73
1516.69
1655.83
2063.04
1229.66
2201.95
1073.52
2129.98
2298.97
1356.73
1747.87
1766.80
1839.92
2430.05
2517.32

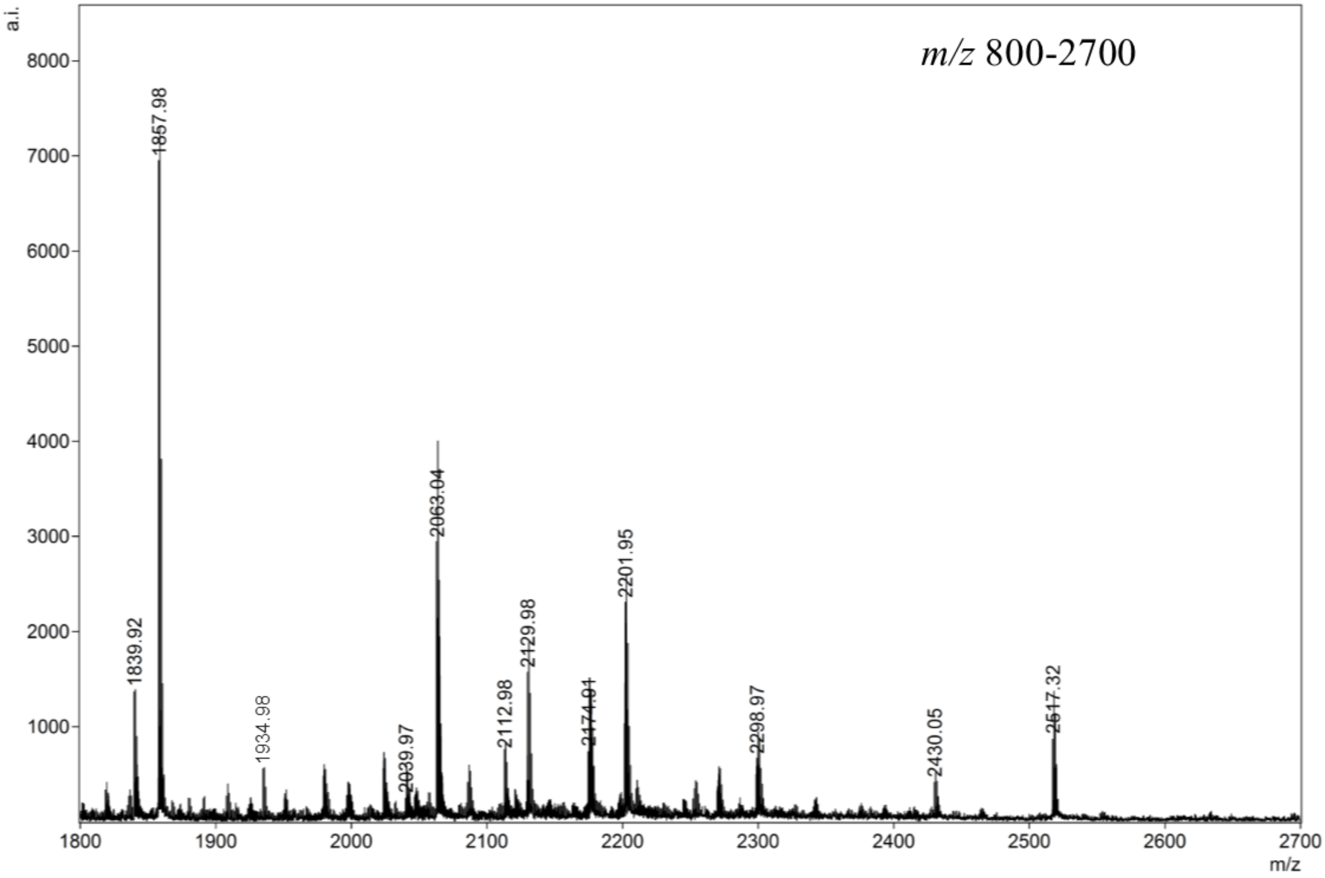

m/z 800-2700
a.i.
m/z
1857.98
2063.04
2201.95
2129.98
1839.92
1934.98
2112.98
2174.91
2298.97
2039.97
2430.05
2517.32

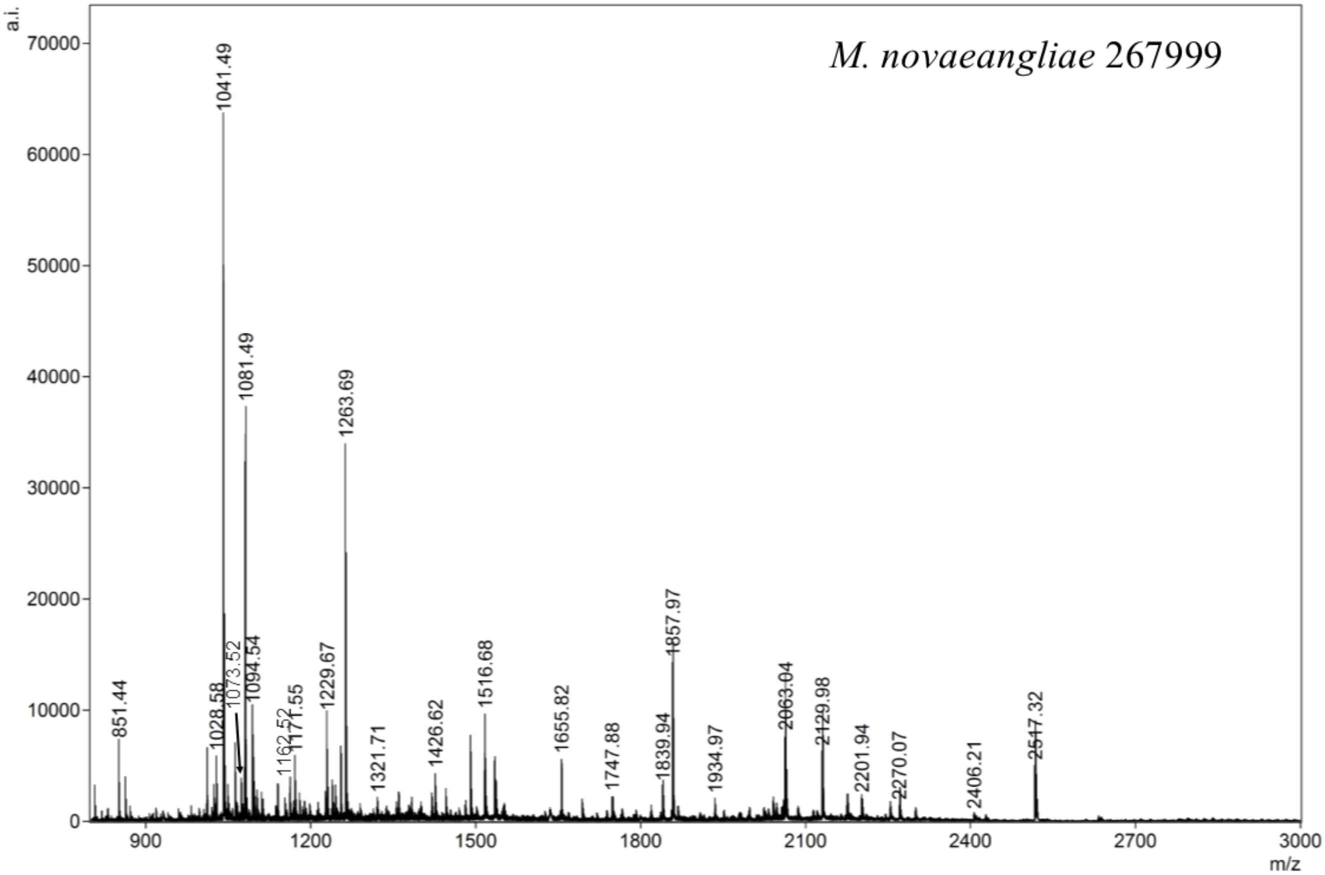
M. novaeangliae 267999
a.i.
m/z
1041.49
1081.49
1263.69
1857.97
851.44
1028.58
1073.52
1094.54
1162.52
1171.55
1229.67
1321.71
1426.62
1516.68
1655.82
1747.88
1839.94
1934.97
2063.04
2129.98
2201.94
2270.07
2406.21
2517.32

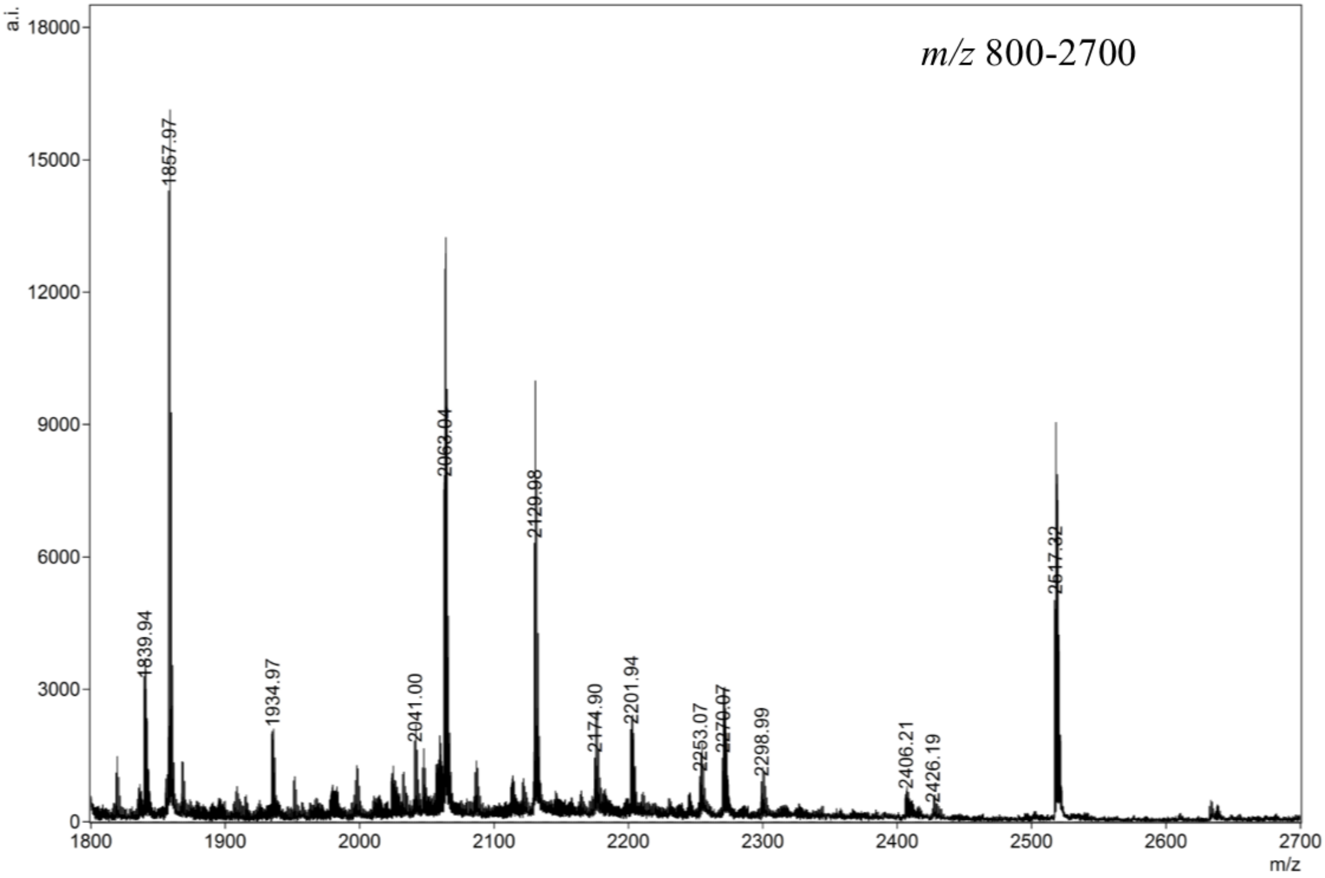
m/z 800-2700
a.i.
m/z
1857.97
1839.94
1934.97
2041.00
2063.04
2129.98
2174.90
2201.94
2253.07
2270.07
2298.99
2406.21
2426.19
2517.32

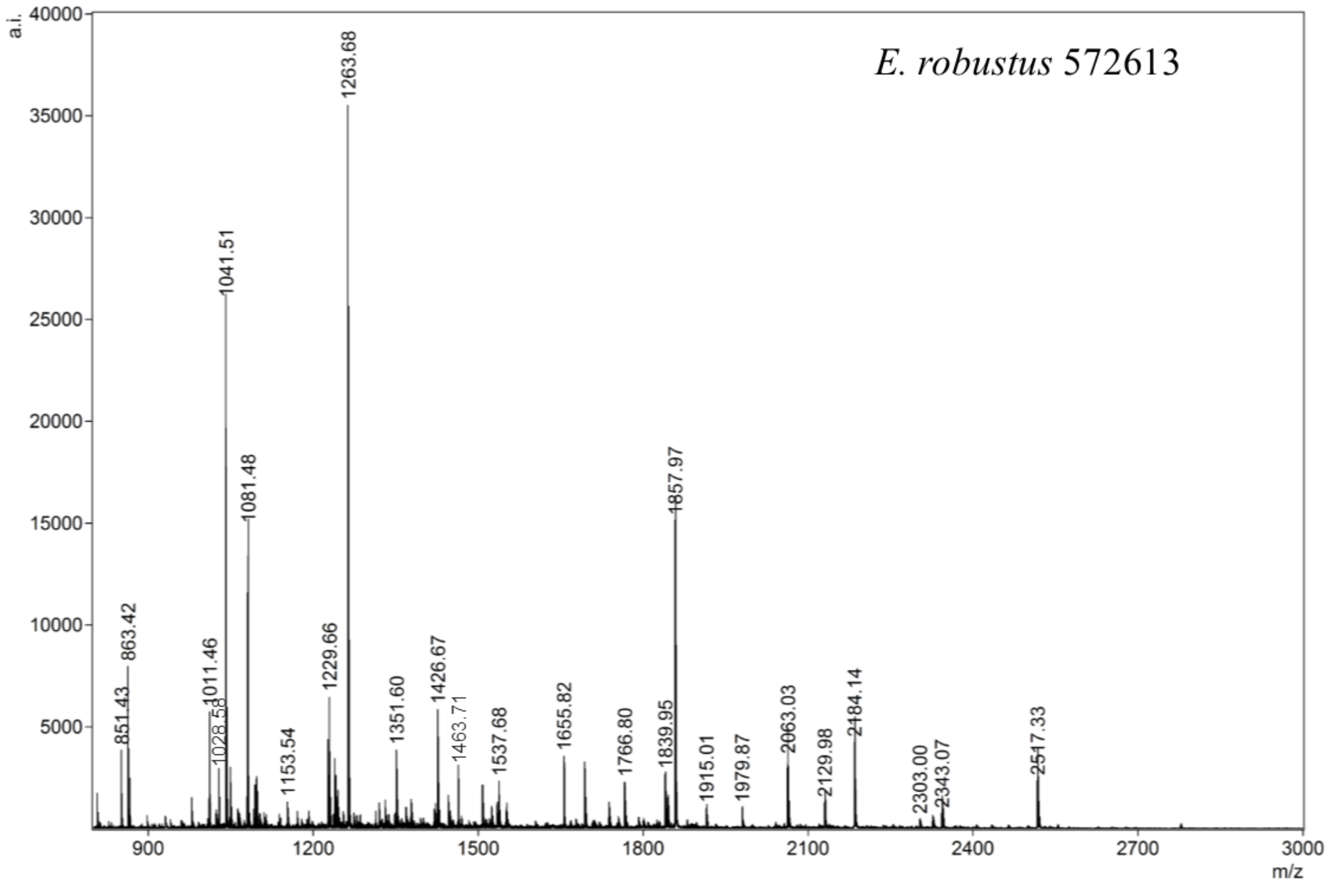
E. robustus 572613
a.i.
m/z
851.43
863.42
1011.46
1028.58
1041.51
1081.48
1153.54
1229.66
1263.68
1351.60
1426.67
1463.71
1537.68
1655.82
1766.80
1839.95
1857.97
1915.01
1979.87
2063.03
2129.98
2184.14
2303.00
2343.07
2517.33

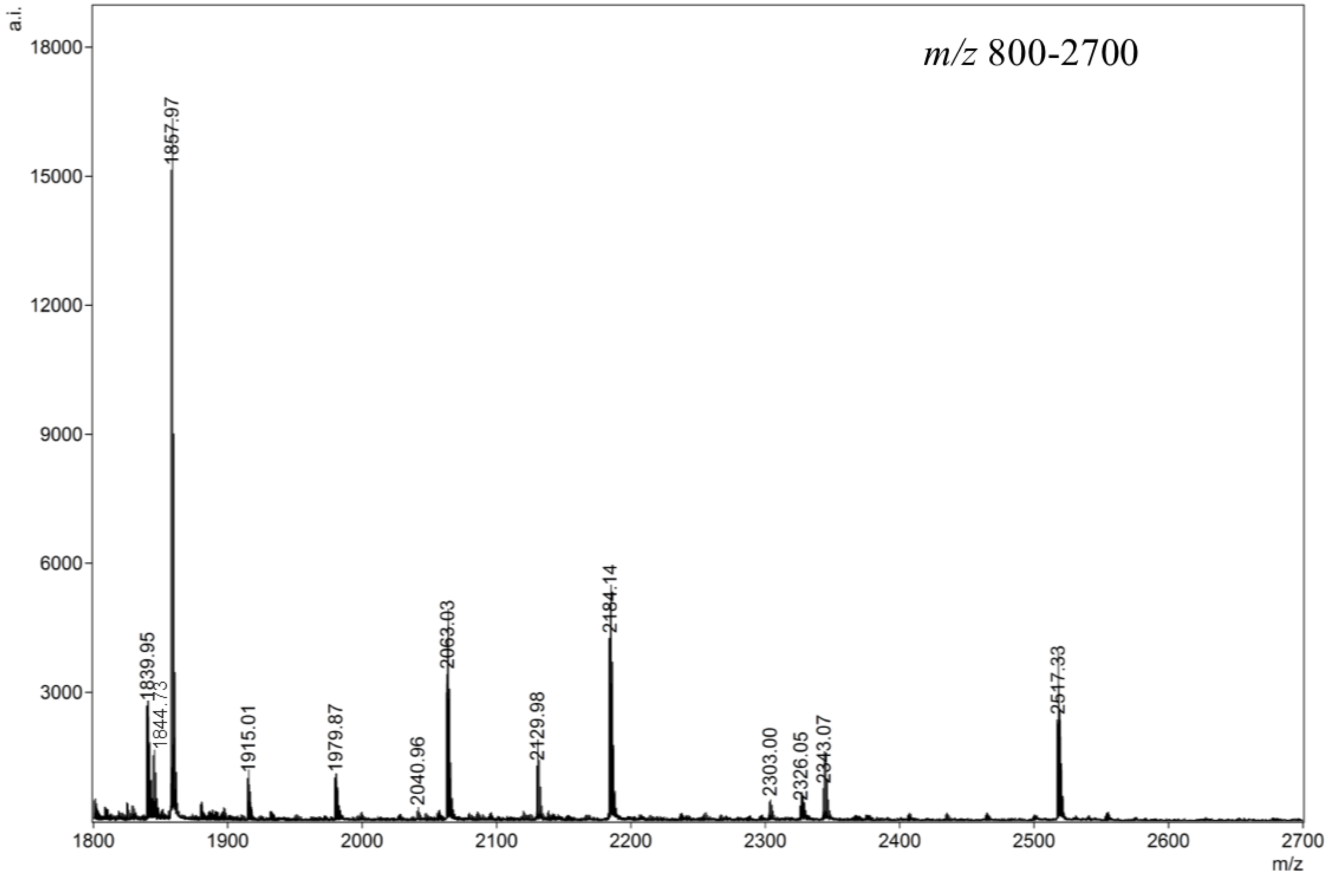
m/z 800-2700
a.i.
m/z
1839.95
1844.73
1857.97
1915.01
1979.87
2040.96
2063.03
2129.98
2184.14
2303.00
2326.05
2343.07
2517.33

YSSQLAQIQGLISNVEAQLSEIR: *m/z* 2547.34

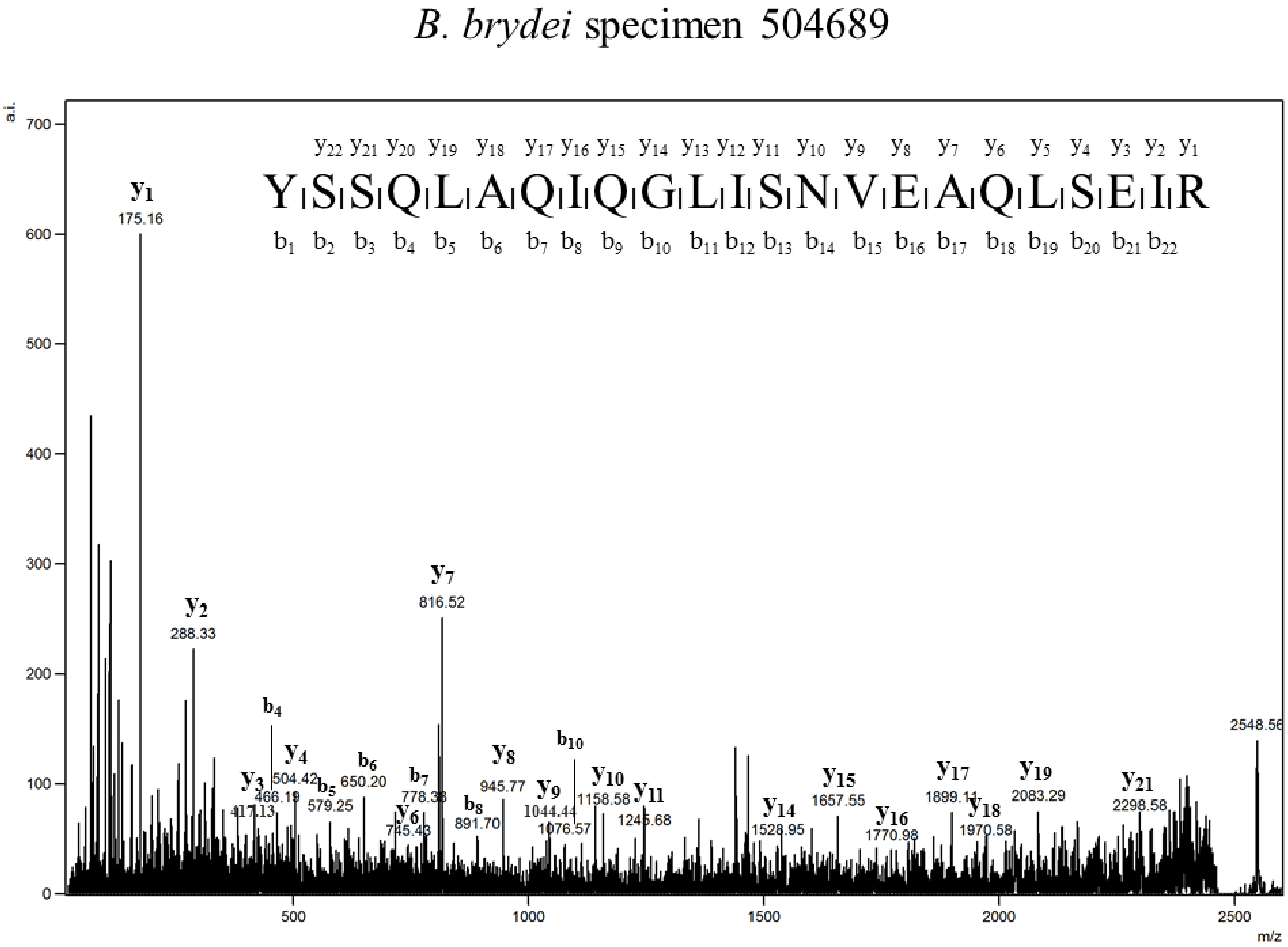

YSSQLAQIQGLIGNVEAQLSEIR: *m/z* 2517.33

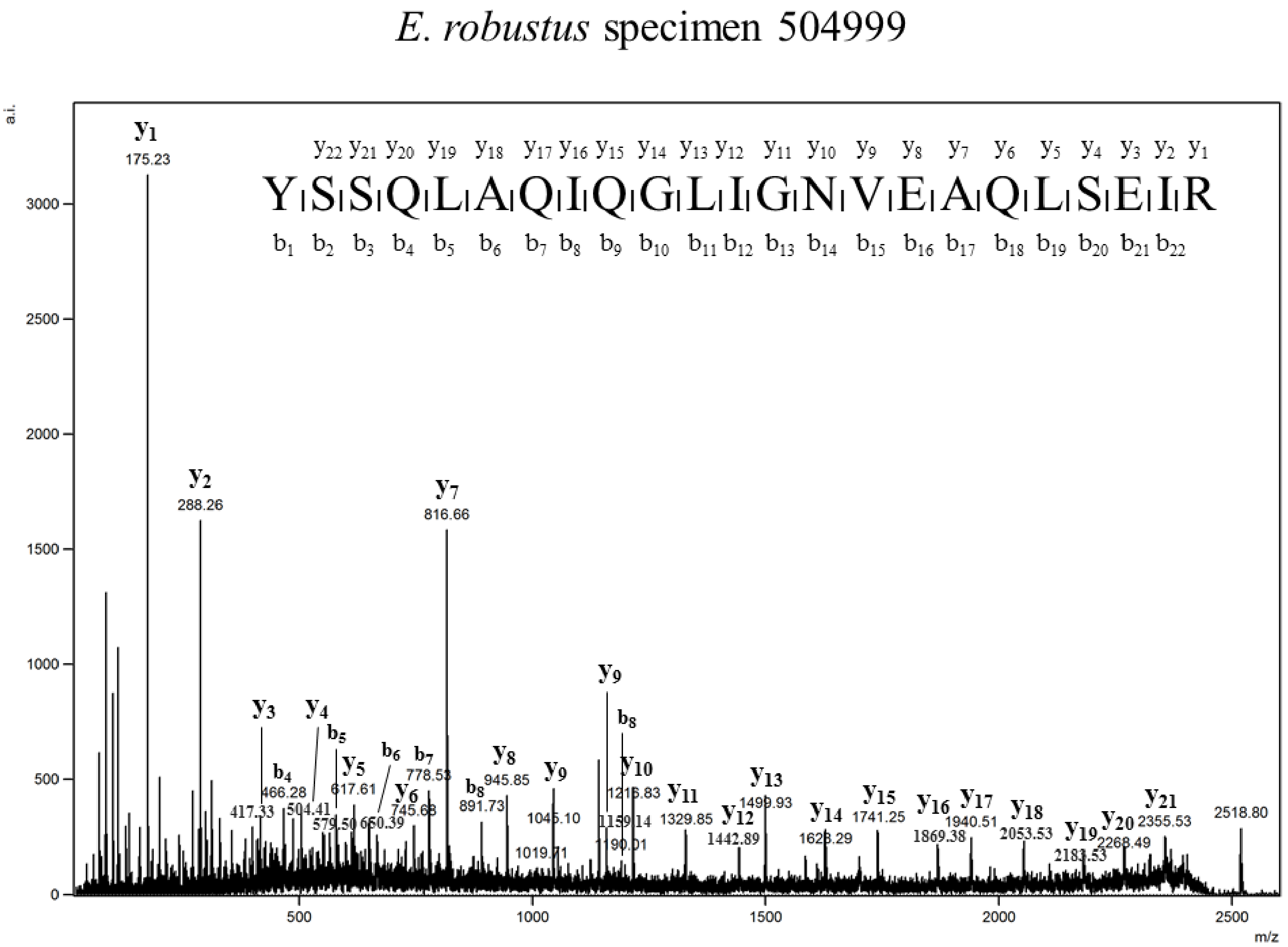

YTSQLAQIQCLISNVEAQLSEIR: *m/z* 2664.37

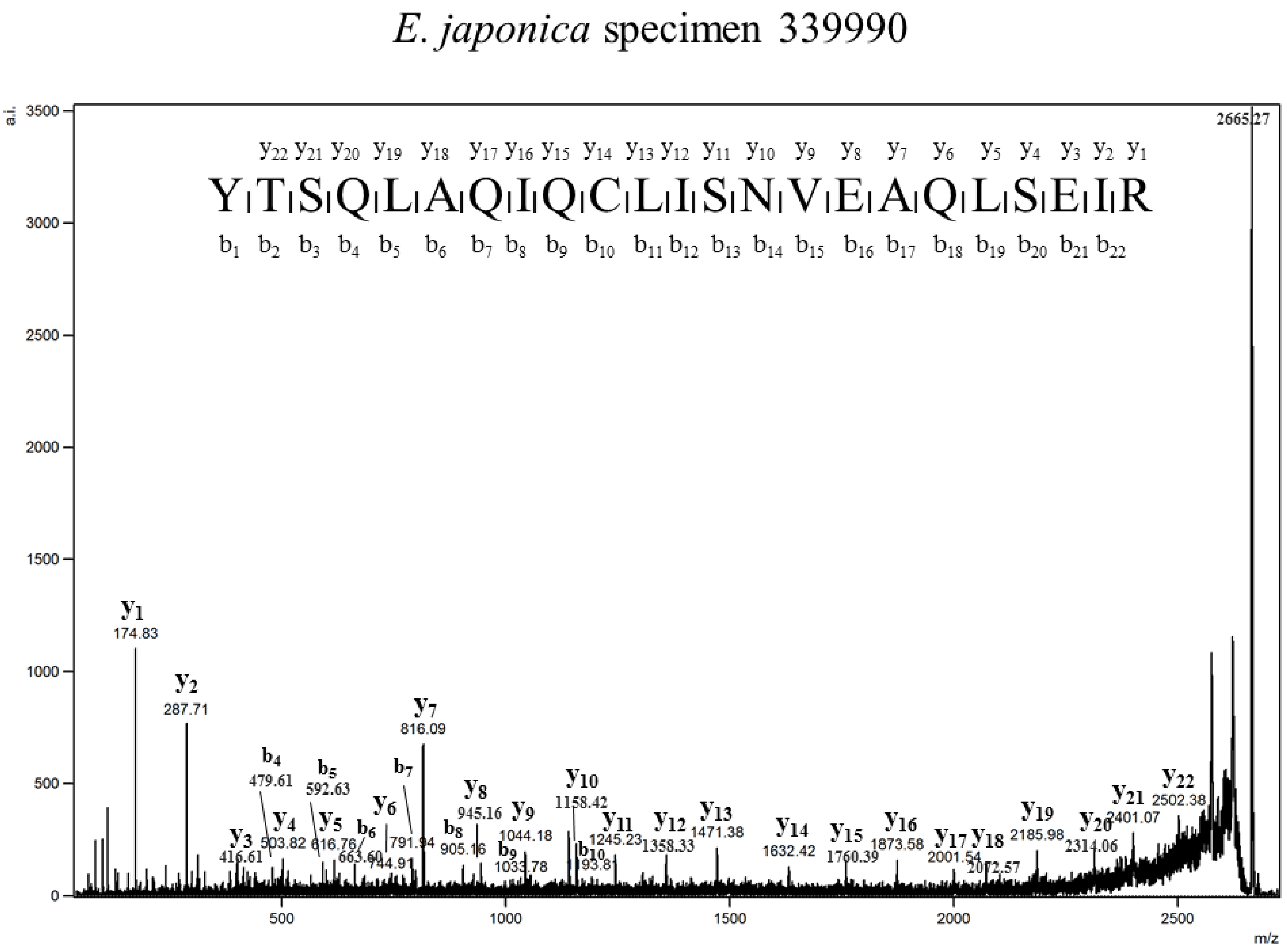

APYISSVPCAPAPQLSTQIR (with Carbamidomethylation C): *m/z* 2156.12

B. brydei specimen 504074

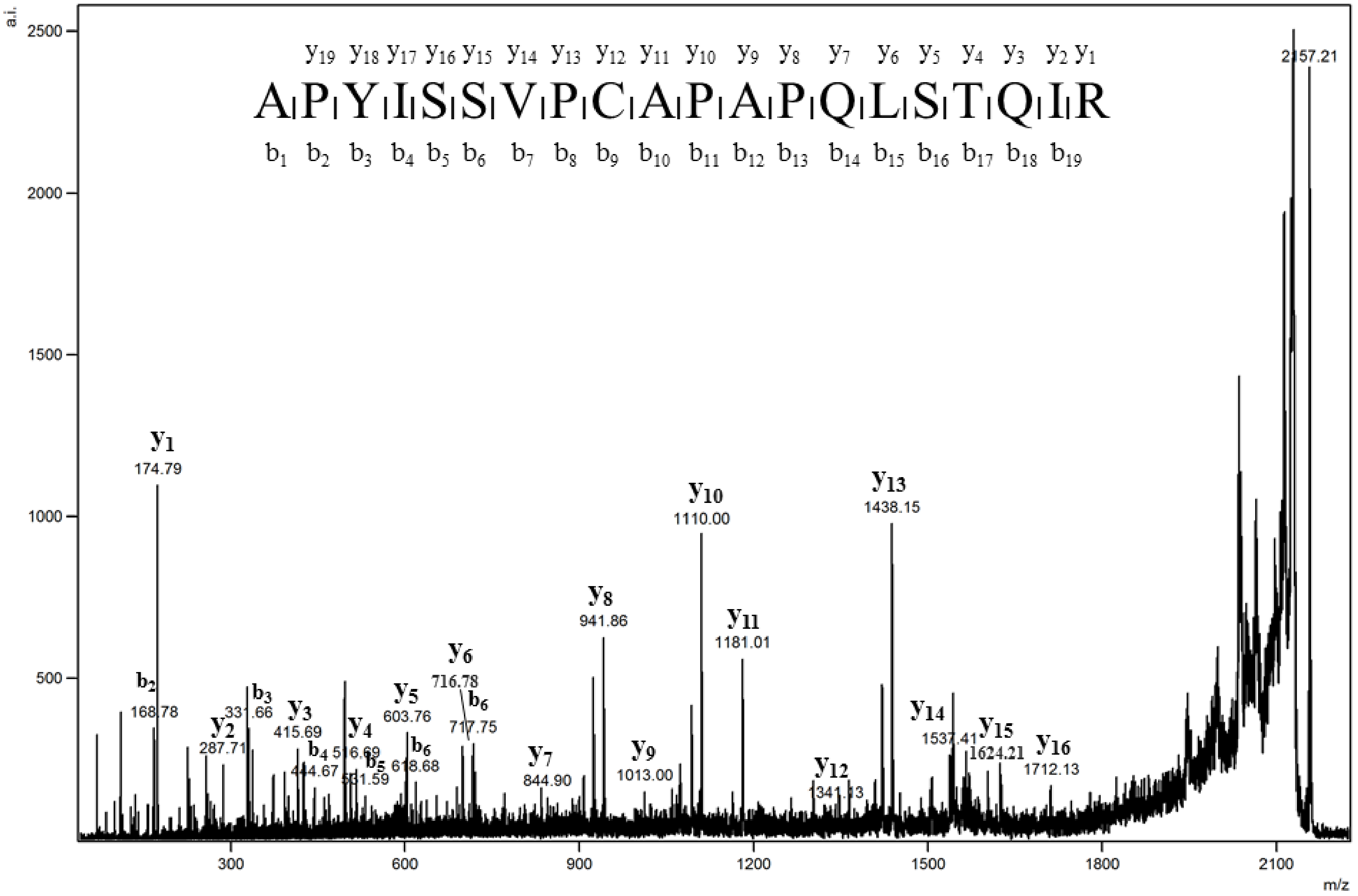

VPYISSVPCAPAPQLSTQIR (with Carbamidomethylation C): *m/z* 2184.15

E. japonica specimen 339990

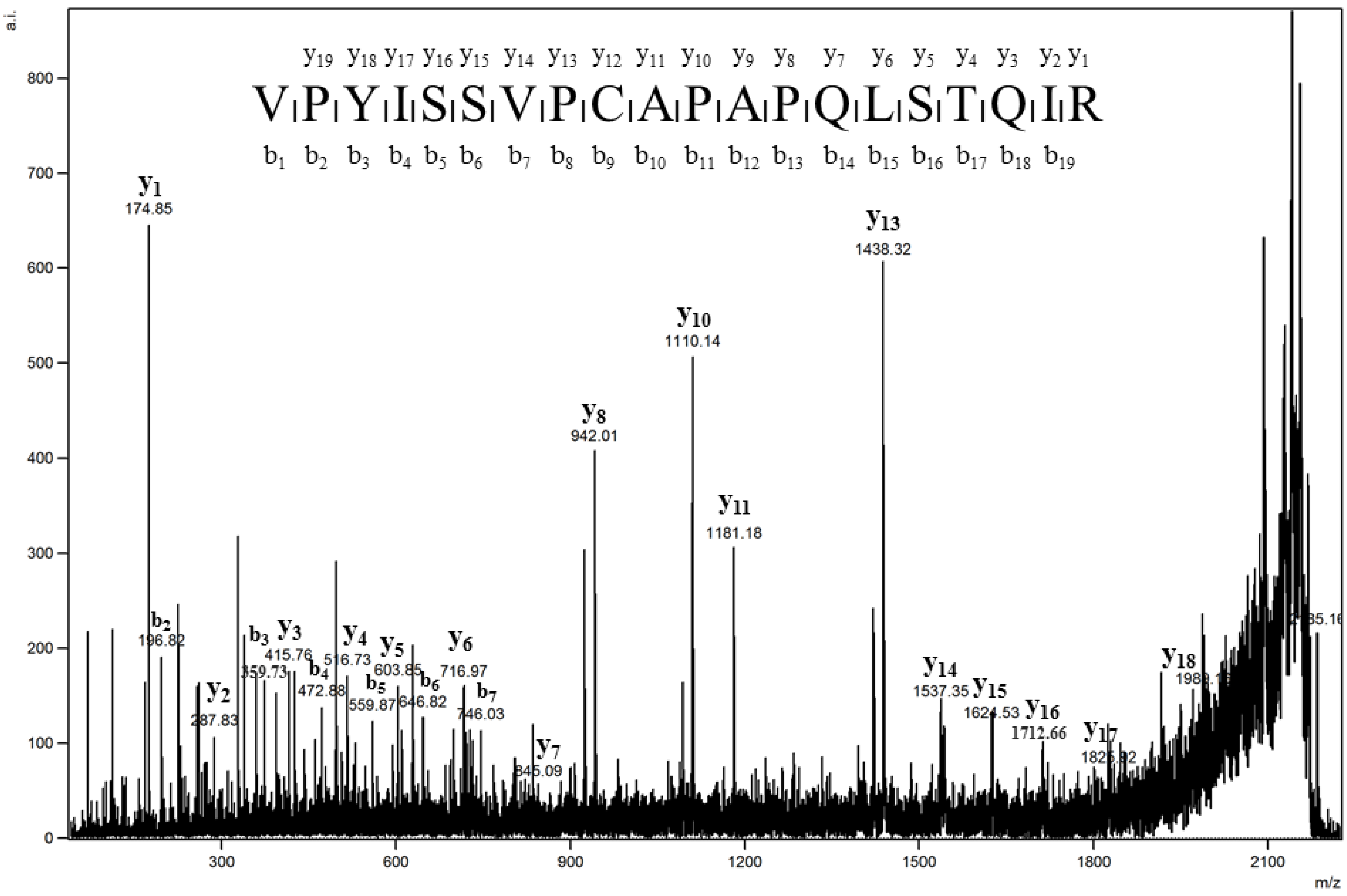

Supplement: S4 File — (PDF) [file pone.0183053.s004.pdf]
